# Microviscosity-gated excited-state partitioning in a NIR-II molecular rotor for disease imaging and imaging-guided photothermal therapy

**DOI:** 10.1016/j.mtbio.2026.103342

**Published:** 2026-06-10

**Authors:** Yufei Qin, Yaru You, Yishu Yu, Yiling Xie, Yating Sha, Jinxin Feng, Yingnan Zeng, Jiaqi Zhang, Ziyi Lei, Caicai Lu, Mingxi Fang, Mengchao Cui, Kaixiang Zhou

**Affiliations:** a Center for Advanced Materials Research & Faculty of Arts and Sciences, Beijing Normal University, Zhuhai, 519087, PR China; b School of Medical Imaging, Xuzhou Medical University, Xuzhou, Jiangsu, 221006, PR China; c Experimental Teaching Platform, Beijing Normal University, Zhuhai, 519087, PR China; d Key Laboratory of Radiopharmaceuticals, Ministry of Education, College of Chemistry, Beijing Normal University, Beijing, 100875, PR China

**Keywords:** Microviscosity, Near-infrared II, Molecular rotors, Photothermal therapy, Acceptor engineering, Liver disease imaging

## Abstract

Microviscosity is an underexploited pathological cue that can be harnessed to regulate excited-state energy dissipation in organic near-infrared II (NIR-II, 1000-1700 nm) fluorophores. Here we introduce a twisted intramolecular charge transfer molecular rotor that uses microviscosity to gate radiative versus nonradiative decay, delivering a fluorescence “off” state in low-viscosity environments and a weak-but-sufficient NIR-II fluorescence turn-on in viscosity-elevated pathology while keeping nonradiative dissipation dominant for photothermal heating. A tunable donor-π-acceptor library was synthesized to drive absorption/emission toward the NIR-II window and calibrate a “dim-but-hot” photophysical profile. Molecularly dispersed **FMR-1105-PEG** enables *in vivo* NIR-II imaging in nonalcoholic fatty liver disease and acetaminophen-induced liver injury models. For tumor translation, micellization retains a measurable viscosity dependence (Förster-Hoffmann-type) while markedly increasing the apparent molar extinction coefficient, thereby enabling efficient 1064 nm photothermal activation, immunogenic cell death hallmarks *in vitro*, and imaging-guided tumor ablation even under tissue coverage, with favorable biosafety.

## Highlights

• Microviscosity-gated NIR-II fluorophore modulates excited-state dissipation.
• TICT rotor design enables viscosity-responsive NIR-II imaging *in vivo*.
• Micellar formulation enhances NIR-II absorption and photothermal conversion.
• Theranostic platform integrates viscosity sensing with photothermal therapy.

## Graphical abstract

**Figure:**
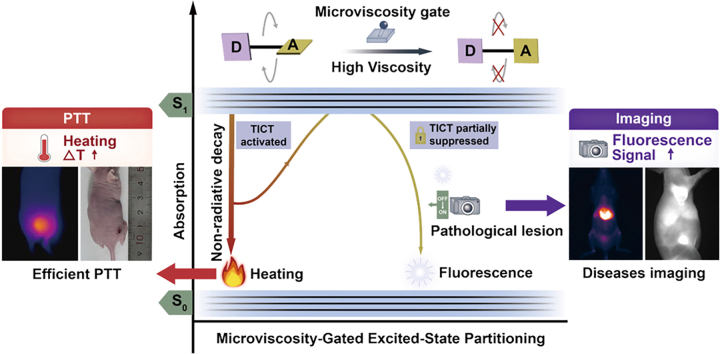

## 1 Introduction

Theranostics, which integrates disease diagnosis and therapy into a single platform, offers significant advantages over traditional approaches by enabling real-time disease visualization and treatment, improving precision and reducing side effects [1,2]. For malignant tumors, conventional treatments such as surgery, chemotherapy, radiotherapy, and immunotherapy have improved clinical outcomes but are still limited by invasiveness, systemic toxicity, damage to surrounding normal tissues, drug resistance, or variable patient responses [3]. In this context, photothermal therapy (PTT) provides a complementary therapeutic route because it enables localized, minimally invasive, and externally controllable tumor ablation [4]. Among various modalities, PTT converts light into localized heat to induce tumor cell death with high spatiotemporal controllability [5,6]. When combined with fluorescence imaging, near-infrared II (NIR-II, 1000–1700 nm) fluorescence-guided PTT provides deeper tissue penetration, reduced scattering, and enhanced therapeutic precision, making it especially useful for deep-seated lesions [[7], [8], [9]]. The use of NIR-II absorption further improves tissue penetration and allows for higher energy delivery with reduced superficial heating, making it particularly beneficial for safe and effective PTT in deeper tissues [[10], [11], [12], [13], [14], [15]].

Organic NIR-II small molecules offer advantages such as tunable photophysical properties, biocompatibility, and straightforward metabolism [[16], [17], [18], [19]]. However, most existing NIR-II theranostics systems either use multi-component platforms or focus on either fluorescence (high quantum yield, QY; low nonradiative decay) [[20], [21], [22], [23], [24]] or photothermal conversion (dominant nonradiative decay, quenched fluorescence) [[25], [26], [27], [28], [29], [30], [31]], leading to a design conflict: improving fluorescence typically reduces heat generation, while optimizing photothermal efficiency compromises imaging brightness. Several strategies have been proposed to address this balance, including controlling molecular motions [[32], [33], [34], [35], [36]], structure size-driven intermolecular interactions [37], forming luminescent liquid crystals [38], regulation of backbone distortion [39], human serum albumin binding [40], nitrilation strategy [41]. While these approaches show promise, further exploration is needed to optimize their performance in complex biological environments. These methods primarily focus on regulating radiative and non-radiative decay pathways but often overlook the dynamic nature of the physiological microenvironment, which is crucial in determining molecular behavior *in vivo*.

To reconcile the conflict between emission and heat generation within a single organic NIR-II small molecule, it is essential to not only fine-tune the radiative versus nonradiative decay channels but also to couple this balance with specific pathological cues. In this regard, microviscosity has recently been recognized as an important biophysical hallmark of various diseases. This feature is particularly relevant to liver diseases, where current clinical evaluation mainly relies on serum biochemical markers, histopathological examination, and conventional imaging. These methods are useful but often provide limited information on early molecular-level microenvironmental changes and are not ideal for real-time longitudinal monitoring [42,43]. Excessive lipid accumulation and organelle remodeling in nonalcoholic fatty liver disease (NAFLD), as well as acute hepatocellular stress in drug-induced liver injury (DILI), can both reshape the hepatic intracellular microenvironment and perturb local microviscosity [[43], [44], [45], [46]]. Solid tumors, on the other hand, represent pathological microenvironments with abnormal crowding, elevated interstitial pressure, and altered rheology [[47], [48], [49], [50]]. Twisted intramolecular charge transfer (TICT)-based fluorescent molecular rotors are particularly suitable for sensing viscosity: in low-viscosity media, free intramolecular rotation opens a fast nonradiative decay pathway and leads to weak emission; as viscosity increases, rotational motion is progressively restricted, the TICT channel is partially suppressed, and fluorescence is “turned on” [[51], [52], [53], [54]].

**Fig. 1:**
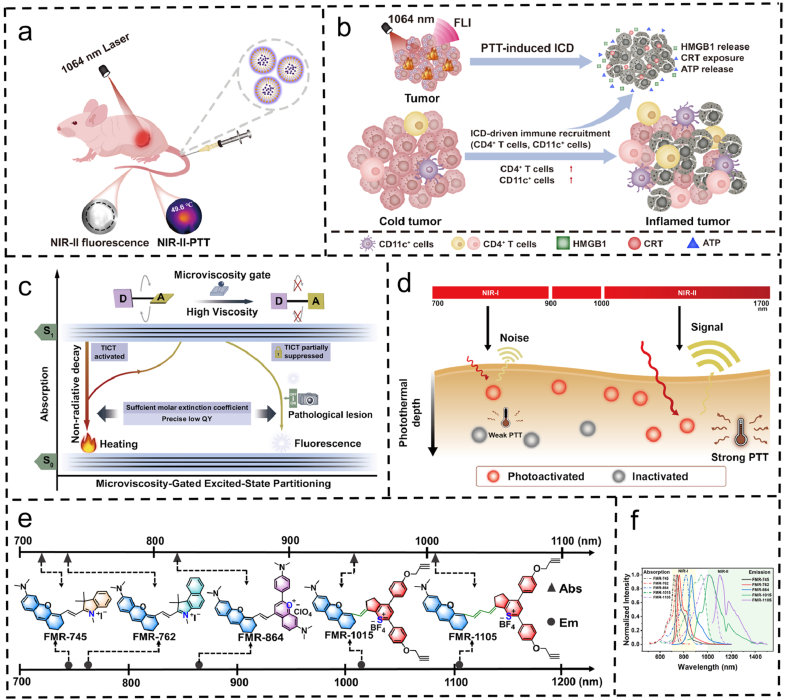
Microviscosity-gated “dim-but-hot” NIR-II molecular-rotor theranostics and molecular design of the **FMRs** library. (a) Schematic illustration of the stage-wise theranostic workflow using the same fluorophore: microviscosity-linked NIR-II fluorescence imaging for lesion localization, followed by 1064 nm-triggered PTT. (b) Conceptual mechanism of PTT-induced ICD and the associated tumor microenvironment remodeling, including representative ICD hallmarks (HMGB1 release, CRT exposure, and ATP-related signals) and immune-cell-related cues. (c) Working principle of microviscosity-gated excited-state partitioning in a TICT molecular rotor: low viscosity permits free rotation to favor TICT-mediated nonradiative decay (fluorescence off), whereas elevated viscosity restricts rotation to partially suppress TICT, turning on weak but sufficient NIR-II fluorescence while keeping nonradiative heating dominant. (d) Illustration of reduced scattering and deeper tissue penetration under NIR-II excitation for imaging and photothermal activation. (e) Molecular structures of the **FMRs** fluorophore library showing stepwise tuning of conjugation length and heteroatom-modulated (N, O, S) acceptors to shift absorption/emission toward the NIR-II window. (f) Representative normalized absorption and emission spectra of the **FMRs**.

We envisioned that, by using microviscosity to gate the competition between radiative and nonradiative decay, it should be possible to construct a molecular system that (i) remains essentially non-emissive in normal, low-viscosity tissues to provide a silent background, (ii) produces a distinct NIR-II fluorescence turn-on in viscosity-elevated pathological microenvironments for disease monitoring and imaging-guided precision therapy, and (iii) still dissipates the majority of the excited-state energy through nonradiative pathways to enable efficient PTT (Fig. 1c). NAFLD serves as a prototypical viscosity-associated metabolic disease, whereas the acetaminophen-induced DILI model provides an acute and clinically relevant liver-injury setting to evaluate the probe response to microenvironmental remodeling. Solid tumors further offer a therapeutically important context where rheological abnormalities can elevate effective microviscosity and benefit high-contrast NIR-II delineation. Increasing microviscosity is expected to partially suppress TICT relaxation to provide a modest NIR-II readout, while nonradiative-dominant dissipation is intentionally retained to support photothermal heating. Bridging these distinct disease contexts through a shared viscosity-sensitive readout provides a unified theranostic rationale across diverse pathologies. In addition, maximizing the molar extinction coefficient (MEC) and strengthening absorption in the NIR-II region expand the photon-harvesting budget, benefiting both NIR-II signal generation and photothermal heating under practical excitation conditions (Fig. 1d) [55,56].

Herein, we report **FMR-1105**, a NIR-IIb activable TICT molecular-rotor fluorophore, and its water-soluble derivative **FMR-1105-PEG**, which collectively enable a stage-wise NIR-II theranostic workflow (Fig. 1a). In the molecularly dispersed form, **FMR-1105-PEG** acts as a microenvironment-coupled probe: it remains effectively silent in low-viscosity settings yet exhibits a viscosity-activated NIR-II fluorescence enhancement for lesion localization and monitoring in hepatic pathology, as exemplified by NAFLD and drug-induced DILI models. For cancer-oriented imaging and therapy, **FMR-1105-PEG** was further formulated into DSPE-PEG2000 micelles to amplify effective light harvesting and enable tissue-tolerant 1064 nm PTT; notably, micellization retains a clear and measurable (albeit attenuated) viscosity-dependent NIR-II fluorescence readout, while nonradiative dissipation remains dominant to support therapeutic heat generation for efficient PTT. Collectively, these results establish an integrated, stage-wise strategy that couples microviscosity-linked NIR-II readout with formulation-amplified PTT across distinct pathological contexts, with PTT-associated immunogenic cell death (ICD) hallmarks and immunostaining-consistent immune-cell presence serving as supportive biological correlates (Fig. 1b).

## 2 Methods

General experimental information, synthetic procedures, and characterization; detailed *in vivo* NIR-II imaging protocols; QY calculations; viscosity sensitivity tests; theoretical calculations; cell culture and cytotoxicity evaluation; preparation of **FMR-1105-PEG** micelles; photothermal performance and PCE calculations; live/dead cell staining; immunostaining; ATP release measurements; *in vivo* photothermal therapy; immunohistochemistry; animal handling; and establishment of animal models of NAFLD, acetaminophen-induced DILI, and 4T1 tumor-bearing nude mice are provided in the Supporting **Information**. All animal procedures were conducted in strict accordance with institutional guidelines and were approved by the Animal Ethics Committee of Beijing Normal University, Zhuhai.

## 3 Results and discussion

**Fluorescent molecular rotor design and engineering.** To translate the viscosity-gated theranostic concept into a realizable molecular platform, we constructed a five-member donor–π–acceptor (D–π–A) chromophore library (**FMRs**) in which the photophysical landscape can be tuned through two orthogonal, structure-encoded knobs. As shown in Fig. 1e, firstly, we systematically varied the acceptor heteroatom to modulate intrinsic radiative branching and establish a causal structure–QY relationship: **FMR-745** and **FMR-762** are two N-containing (indolium-type) acceptor [45,57] variants that differ only in the nitrogen-based acceptor structure, included to rule out motif-specific idiosyncrasies and to generalize the heteroatom effect within the same framework; **FMR-864** features an O-containing (flavylium-type) [23,58] acceptor as a desulfurized reference; and **FMR-1015** and **FMR-1105** employ S-containing (thiopyrylium-type) [22,24,59] acceptors designed to access an intentionally low-QY regime compatible with photothermal operation. This N/O/S heteroatom series provides an internal control set to decouple “brightness optimization” from “thermally favorable deactivation,” and to clarify how acceptor identity biases the radiative/nonradiative balance without altering the overall donor–acceptor architecture. Second, within the thiopyrylium subset we introduced a D-π-A coupling-length gradient by varying the number of conjugated double-bond linkers, providing a direct structural handle to strengthen intramolecular charge transfer, drive the absorption into the NIR-II window, and tune conformational flexibility relevant to TICT-associated relaxation. Together, acceptor heteroatom modulation and conjugation-length adjustment define a compact yet informative design matrix for mapping structure to NIR-II optical characteristics, calibrated low-QY behavior, and photothermal suitability.

**Fig. 2:**
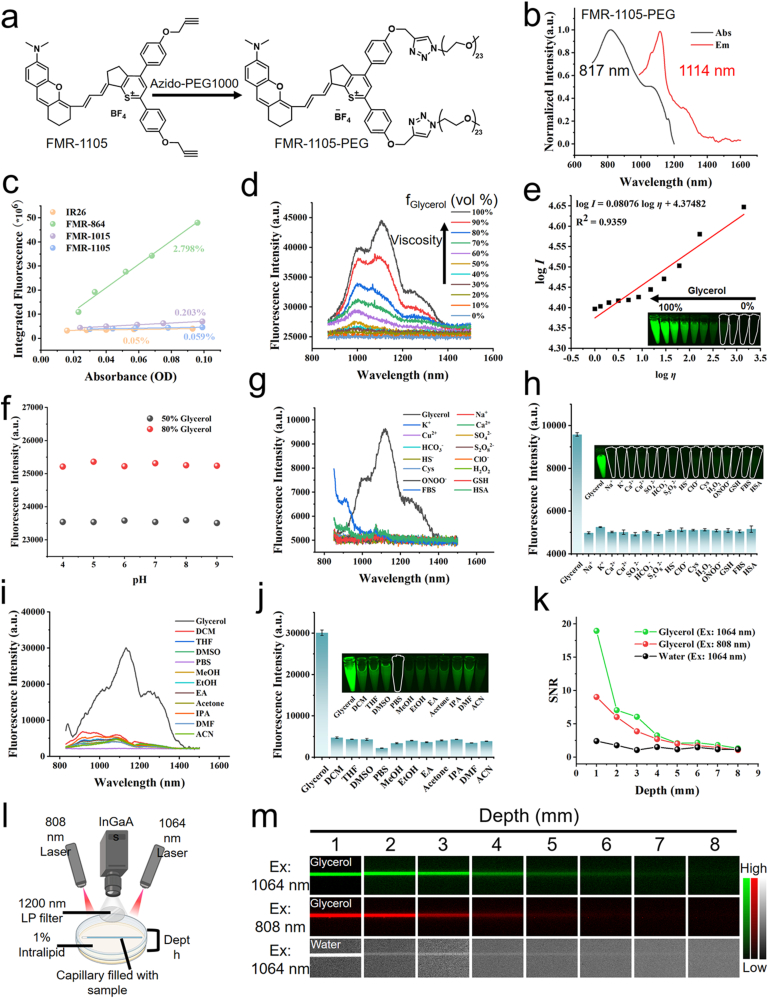
Photophysics, and viscosity-responsive imaging performance of **FMR-1105-PEG**. (a) PEGylation of **FMR-1105** to afford the water-solubility. (b) The absorption and fluorescence emission spectra of **FMR-1105-PEG** in water. (c) Relative QY determination of the **FMRs** using **IR26** as the reference (integrated fluorescence emission vs absorbance). (d) NIR-II fluorescence spectra in glycerol/water mixtures with increasing glycerol content (increasing viscosity), showing a viscosity-dependent fluorescence turn-on. (e) Förster–Hoffmann analysis of fluorescence enhancement as a function of microviscosity, with inset NIR-II images. (f) Fluorescence intensity of **FMR-1105-PEG** versus pH in different glycerol fractions. (g-h) Anti-interference evaluation of the fluorescence response of **FMR-1105-PEG** toward representative biologically relevant species and ions, with inset NIR-II fluorescence images in (h). (i-j) NIR-II emission spectra (i) and fluorescence intensity (j) of **FMR-1105-PEG** in media of diverse polarity, with inset fluorescence images in (j). Data in (h, j) are shown as mean ± SD, n = 3 independent experiments. (k) SNR as a function of imaging depth for capillary samples under 808 nm or 1064 nm excitation in low-versus high-viscosity media. (l) Schematic of the depth-imaging setup (capillary filled with sample embedded in intralipid; detection with a 1200 nm LP filter). (m) Representative NIR-II images collected at increasing depths (1 - 8 mm) under different excitation conditions.

**Synthesis and Photophysical characterization.** We first constructed the thiopyrylium-based electron-acceptor module through a multistep synthesis (Scheme S1). Then the five target molecular rotors were subsequently assembled in a modular fashion via efficient Knoevenagel condensations, enabling systematic variation of the acceptor heteroatom (N/O/S) and the conjugation length (Scheme S2-3). Full experimental details, purification, and structural characterization are provided in the Supporting Information. We carried out a rapid photophysical screening of the **FMRs** library to establish an overall structure-property landscape and to identify a lead dye for further studies. As summarized in Fig. 1f and Table S1, the five FMR dyes collectively cover a broad NIR optical window, with λ_abs_ spanning 716-1007 nm and λ_em_ spanning 745-1105 nm, together with systematically tunable MEC (5.31 × 10^4^ - 16.00 × 10^4^ M^−1^ cm^−1^, Fig. S1), QY (0.06 - 20.67%, Fig. 2c–S3-5), and Brightness (44 - 29118 M^−1^ cm^−1^). In particular, **FMR-1015** and **FMR-1105**, extend emission beyond 1000 nm, and extending the π-conjugation from **FMR-1015** to **FMR-1105** further drives the absorption across the NIR-II threshold (from 948 nm to 1007 nm), enabling true NIR-II excitation compatibility. Within this context, **FMR-1105** provides the deepest NIR-II positioning (λ_abs_/λ_em_ = 1007/1105 nm) together with the highest MEC (7.54 × 10^4^ M^−1^ cm^−1^), ensuring a strong photon-harvesting budget under NIR-II excitation. Meanwhile, its deliberately low QY (0.06%; Brightness = 44 M^−1^ cm^−1^) indicates a strongly suppressed radiative branch, consistent with a nonradiative-dominant energy partitioning that is compatible with photothermal operation. Therefore, **FMR-1105** was selected as the lead scaffold for downstream modification and bio-relevant evaluations.

**Theoretical calculations**. To further support the molecular design rationale, density functional theory (DFT) and time-dependent DFT (TD-DFT) calculations were performed for the **FMR** series. As shown in Fig. S7, the optimized geometries confirmed their twisted D-π-A molecular-rotor structures, while the frontier molecular orbital analysis revealed a gradual decrease in the Highest Occupied Molecular Orbital and Lowest Unoccupied Molecular Orbital (HOMO-LUMO) energy gap from **FMR-745** to **FMR-1105**. The calculated gaps decreased from 2.24 to 1.69 eV, consistent with the progressive red-shift of optical transitions induced by acceptor heteroatom modulation and π-conjugation extension. TD-DFT calculations further showed a decreasing trend in the first excited state (S_1_) transition energy across the **FMR** series. Natural transition orbital (NTO) analysis indicated that the ground state (S_0_) to S_1_ excitation was dominated by the first NTO pair, with major contributions close to 100%, and displayed clear ICT character (Fig. S8). These results demonstrate that acceptor engineering and π-conjugation extension narrow the electronic bandgap and promote ICT-dominated long-wavelength transitions. In particular, the sulfur-containing thiopyrylium acceptor in **FMR-1105** exhibits the strongest excited-state charge separation and the lowest transition energy among the series, which facilitates excited-state relaxation and favors nonradiative energy dissipation over radiative decay compared with the N- and O-containing analogues. Together with the twisted rotor geometry, this ICT/TICT-prone excited state supports nonradiative deactivation in low-viscosity environments and viscosity-activated fluorescence upon restricted intramolecular motion, providing a theoretical basis for the NIR-II fluorescence and photothermal behavior of the **FMR** members.

**Microviscosity-responsive NIR-II fluorescence of FMR-1105-PEG.** To enable aqueous compatibility for biological studies, **FMR-1105** was further PEGylated to afford the water-dispersible probe **FMR-1105-PEG** (Fig. 2a), which improves solubility in physiological media and facilitates downstream bioevaluation [18,60]. In water, **FMR-1105-PEG** retains NIR-II emission, whereas its main absorption band shows an apparent blue shift due to the polarity-associated quenching [22,61]. Importantly, despite the blue-shifted maximum, **FMR-1105-PEG** still exhibits a long-wavelength absorption tail extending into the NIR-II region, preserving compatibility with long-wavelength excitation (Fig. 2b). We then evaluated the microviscosity response of **FMR-1105-PEG** using glycerol/water mixtures as a controllable model system. As illustrated in Fig. 2d and e, **FMR-1105-PEG** shows a pronounced viscosity-dependent NIR-II fluorescence turn-on, and the enhancement follows a Förster–Hoffmann-type correlation, consistent with viscosity-restricted intramolecular motion that redistributes radiative versus nonradiative decay. Meanwhile, the fluorescence signal remains stable across the tested pH range and anti-interference performance in the presence of representative biologically relevant species, supporting its robustness in complex environments (Fig. 2f–h). Moreover, across solvents with varied polarities, **FMR-1105-PEG** exhibits a strongly viscosity-specific NIR-II fluorescence enhancement with negligible influence from solvatochromism, indicating that microviscosity, rather than bulk solvent polarity, predominantly governs its emission output under these conditions (Fig. 2i and j). Similar viscosity-dependent fluorescence enhancements and viscosity-specificity were observed for other **FMR** members (Fig S9-11, 13). Capillary samples containing **FMR-1105-PEG** in low-versus high-viscosity media were imaged at increasing depths under 808 nm and 1064 nm excitation, with detection beyond 1200 nm (Fig. 2l and m). The depth-dependent profiles and quantitative analysis reveal consistently improved signal-to-noise ratio (SNR, Fig. 2k) and better-resolved spot profiles (narrower full widths at half-maximum, FWHM; Fig. S14) in the high-viscosity condition, and these advantages are further amplified under 1064 nm excitation compared with 808 nm, consistent with reduced scattering and more favorable photon propagation at longer wavelengths. Together, these results establish **FMR-1105-PEG** as a water-dispersible, microviscosity-responsive NIR-II probe that not only reports viscosity variations with high specificity but also translates this turn-on behavior into enhanced penetration and contrast in tissue-mimicking media, supporting its suitability for subsequent biological imaging and imaging-guided therapy studies.

**Fig. 3:**
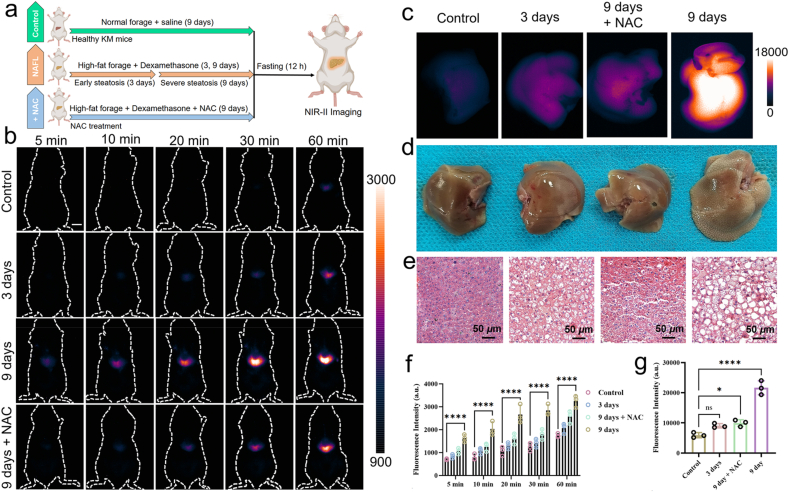
*In vivo* NIR-II imaging of a dexamethasone-induced NAFLD model using molecularly dispersed **FMR-1105-PEG**. (a) Schematic of the NAFLD model building and experimental groups (Control; NAFLD induced by high-fat forage + dexamethasone treatment; NAFLD + NAC intervention) and the NIR-II imaging workflow. (b) Time-course (5 - 60 min) whole-body NIR-II fluorescence images after *i.v.* administration of **FMR-1105-PEG**, showing progressive signal accumulation in the liver region (dashed outline) with markedly enhanced fluorescence in NAFLD mice and signal attenuation upon **NAC** treatment. (c-d) *Ex vivo* NIR-II fluorescence images (c) and photographs (d) of excised livers from the indicated groups. (e) Representative H&E staining of liver sections (scale bar: 50 μm) confirming pathological changes and the mitigation effect of **NAC**. (f) Quantification of liver-region fluorescence intensity from the *in vivo* time-course images (panel b). (g) Quantification of *ex vivo* liver fluorescence intensity (panel c). Data are presented as mean ± SD., ∗p < 0.05; ∗∗∗∗p < 0.0001, n = 3.

***In vivo* viscosity measurement in NAFLD model and therapy intervention.** We next evaluated whether molecularly dispersed **FMR-1105-PEG** could deliver a microenvironment-coupled, rotor-like NIR-II readout *in vivo* in a viscosity-relevant liver pathology. A dexamethasone-assisted NAFLD model was established together with an N-acetylcysteine (NAC) intervention arm (Fig. 3a). After intravenous (*i.v.*) administration of **FMR-1105-PEG**, time-resolved NIR-II imaging revealed rapid liver-localized fluorescence that increased over time, with NAFLD mice showing markedly higher liver-region signal than healthy controls (Fig. 3b–S15-17). Notably, NAC treatment substantially attenuated the hepatic NIR-II signal relative to untreated NAFLD animals (Figs. 3b, 3f, S18), supporting that the imaging contrast tracks NAFLD-associated microenvironmental changes rather than arising solely from nonspecific biodistribution. *Ex vivo* NIR-II imaging of excised livers reproduced the same group-wise trend (Control < NAFLD; NAFLD + NAC attenuation) (Fig. 3c and g) and was consistent with the gross liver appearance (Fig. 3d). Histological examination further corroborated NAFLD-associated hepatic alterations and their mitigation upon NAC treatment (Fig. 3e). Altogether, these results establish **FMR-1105-PEG** as a microenvironment-coupled NIR-II probe capable of reporting NAFLD progression and intervention response *in vivo*, consistent with our viscosity-gated sensing rationale.

**Fig. 4:**
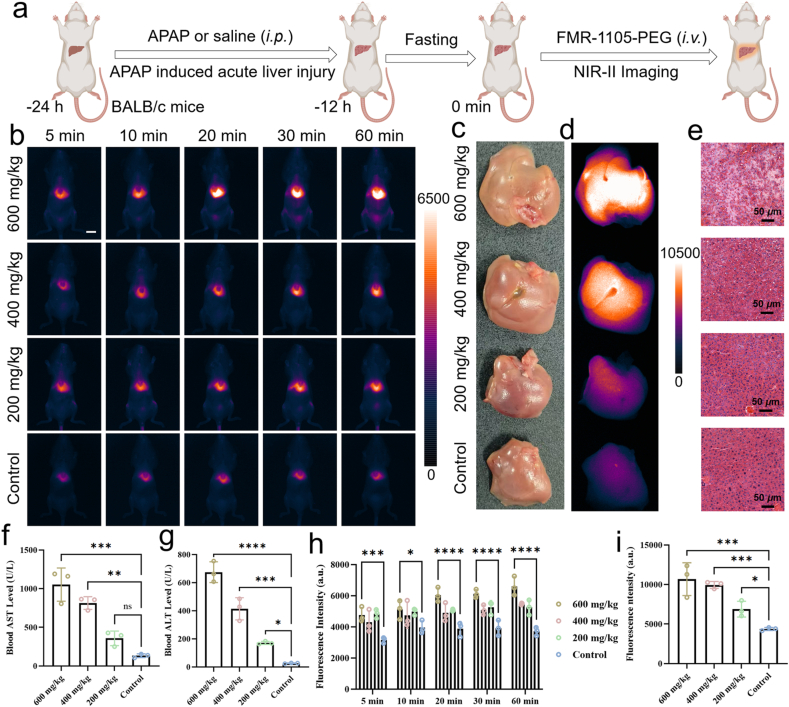
*In vivo* NIR-II imaging of acetaminophen-induced DILI using **FMR-1105-PEG**. (a) Schematic of the acetaminophen-induced acute liver injury model and the NIR-II imaging workflow (APAP or saline *i.p.*, fasting, followed by *i.v.* injection of **FMR-1105-PEG** and NIR-II imaging). (b) Real-time NIR-II fluorescence images after *i.v.* administration of **FMR-1105-PEG** (5 - 60 min) in mice treated with different acetaminophen doses (600, 400, and 200 mg kg^−1^) and saline control, showing dose-dependent enhancement of liver-region fluorescence. (c) Photographs of excised livers from the indicated groups. (d) *Ex vivo* NIR-II fluorescence images of excised livers, confirming elevated hepatic signal in acetaminophen-treated groups. (e) Representative histological detection of liver sections showing acetaminophen-induced pathological changes. (f, g) Serum biochemical markers (AST and ALT) confirming the varying degrees of hepatic injury. (h) Quantification of liver-region fluorescence intensity from the *in vivo* imaging (panel b). (i) Quantification of *ex vivo* liver fluorescence intensity (panel d). Data are presented as mean ± SD., ∗p < 0.05; ∗∗p < 0.01; ∗∗∗p < 0.001; ∗∗∗∗p < 0.0001), n = 3.

**Viscosity activation imaging in acetaminophen-induced DILI model.** To evaluate the generality of this hepatic NIR-II readout beyond a lipid-driven NAFLD context, we further tested an acetaminophen-induced acute DILI model with graded injury severity (Fig. 4a). Following acetaminophen administration at different doses, NIR-II imaging after *i.v.* injection of **FMR-1105-PEG** exhibited a clear, dose-dependent enhancement of liver-region fluorescence in the time-course images (Fig. 4b–S19-22, 4h). *Ex vivo* imaging of harvested livers confirmed the same dose-dependent trend (Fig. 4d and i) and agreed with the gross organ appearance (Fig. 4c). In parallel, serum Aspartate Aminotransferase (AST) and Alanine Aminotransferase (ALT) levels increased with acetaminophen dose (Fig. 4f and g), and histological analysis corroborated acetaminophen-induced hepatic damage (Fig. 4e), together indicating that the NIR-II signal scales with biochemical and histopathological injury severity. While acute DILI is multifactorial, these results suggest that acetaminophen-triggered hepatic injury is accompanied by microenvironmental alterations that modulate the rotor-like NIR-II output of **FMR-1105-PEG** [45,46]. Taken together with the NAFLD study, this second model supports the applicability of molecularly dispersed **FMR-1105-PEG** for NIR-II imaging across distinct liver pathology contexts.

**Fig. 5:**
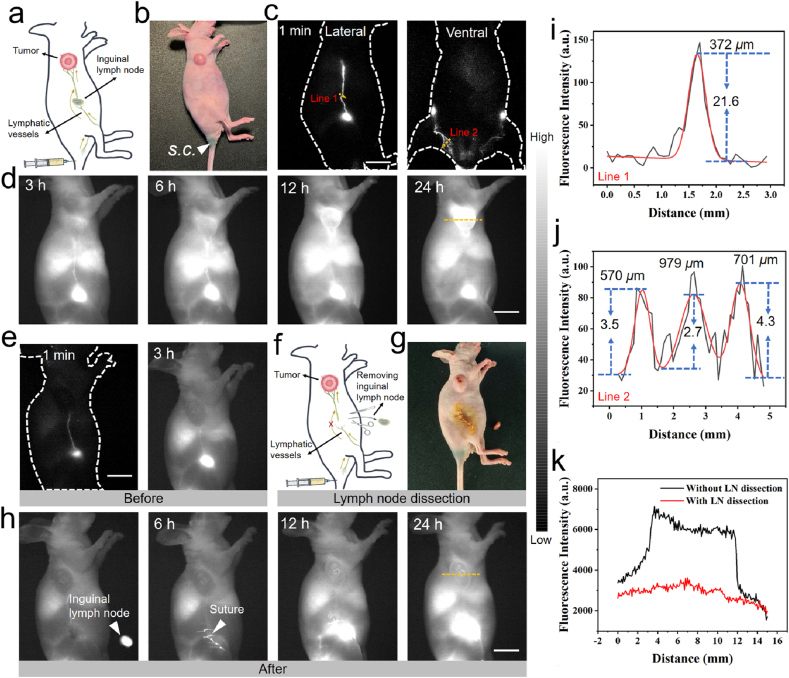
NIR-II SLN-relevant lymphatic mapping with **FMR-1105-PEG** and evaluation of surgery-intervention. (a) Schematic of *s.c*. injection at the tail base and subsequent lymphatic drainage toward the ipsilateral inguinal LN and downstream axillary tumor bed. (b) Photograph showing the tail-base *s.c.* injection site. (c) Representative lateral and ventral whole-body NIR-II images at 1 min post-injection, highlighting early visualization of collecting lymphatic vessels and the ipsilateral inguinal LN. (d) Longitudinal NIR-II imaging at 3, 6, 12, and 24 h revealed stepwise tracer trafficking and a sustained, progressively intensified fluorescence signal in the axillary tumor region. (e) Representative images used for tumor-region intensity comparison before intervention. (f) Schematic of ipsilateral inguinal LN dissection performed at 3 h post-injection. (g) Photograph of the mouse after inguinal LN dissection. (h) Post-dissection NIR-II images at 6, 12, and 24 h (suture location indicated). (i, j) Distance-intensity line profiles extracted from representative lymphatic structures in panel c (line 1, line 2), with FWHM values. (k) Quantification of tumor-region fluorescence intensity demonstrating a pronounced reduction in signal following inguinal LN dissection, consistent with a relay-node–dependent component of lymphatic transport in this model.

**SLN-Relevant NIR-II Lymphatic Mapping and Intervention.** Having established the *in vivo* performance of molecularly dispersed **FMR-1105-PEG** as a microenvironment-coupled NIR-II probe in two liver pathology models, we next explored its broader utility as a water-dispersible NIR-II imaging agent. An emerging fluorescence-guided surgical application is sentinel lymph node (SLN) mapping, where lymphatic tracers delineate drainage pathways and identify first-echelon nodes to support nodal management while minimizing procedure-related morbidity [62,63]. We next translated **FMR-1105-PEG** into a biologically relevant NIR-II imaging application under an SLN-relevant lymphatic mapping scenario (Fig. 5a). After subcutaneous (*s.c.*) administration near the tail-base (Fig. 5b), longitudinal whole-body NIR-II imaging captured stepwise tracer trafficking, with early signals delineating collecting lymphatic vessels and the ipsilateral inguinal lymph node, followed by a delayed yet sustained appearance of fluorescence in the axillary tumor region in this model (Fig. 5c and d). As calculated in Fig. 5i and j, distance-intensity line profiles extracted from representative lymphatic structures enabled quantitative characterization of lymphatic signal geometry using the FWHM, yielding sub-millimeter values (372–979 μm). To incorporate an intervention-mimicking element inspired by node-management concepts in SLN-guided practice, the ipsilateral inguinal lymph node was surgically dissected at 3 h post-injection and imaging was continued thereafter (Fig. 5e–h). Importantly, comparison of tumor-region fluorescence intensity between cohorts demonstrated a pronounced reduction upon inguinal lymph node (LN) dissection, supporting a relay-node–dependent component of lymphatic transport in this experimental setting (Fig. 5k). While not intended to replicate clinical injection protocols or the full complexity of human lymphatic anatomy, this scenario-based model provides a practical preclinical analogue to assess lymphatic route mapping and the sensitivity of downstream readouts to node-level intervention, which is conceptually relevant to SLN-guided lymphatic imaging and decision-making concepts.

**Fig. 6:**
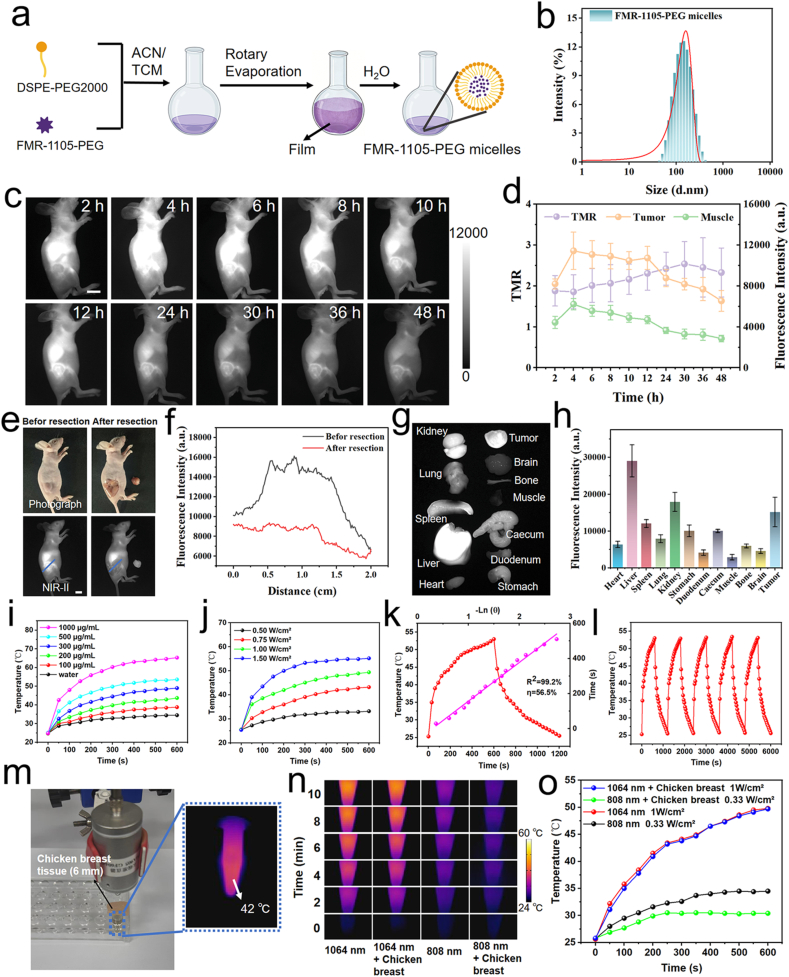
Preparation, tumor NIR-II imaging, biodistribution, and 1064 nm *in vitro* photothermal performance of **FMR-1105-PEG** micelles. (a) Schematic illustration of **DSPE-PEG2000**-assisted micellization of **FMR-1105-PEG**. (b) Hydrodynamic size distribution of the micelles: ∼133 nm. (c) Time-course (2 - 48 h) *in vivo* NIR-II fluorescence imaging of 4T1 tumor-bearing mice after *i.v.* injection of **FMR-1105-PEG** micelles. (d) Quantification of fluorescence intensities in tumor bed and tumor-to-muscle ratio analysis as a function of time post-injection. (e) Representative photographs and corresponding NIR-II images before and after tumor resection, showing tumor delineation of NIR-II fluorescence. (f) Line-scan fluorescence intensity profiles across the tumor region before and after surgery. (g-h) *Ex vivo* NIR-II fluorescence images (g) and quantified *ex vivo* fluorescence intensities (h) of excised tumor and major organs. (i, j) Photothermal heating curves of **FMR-1105-PEG** micelles under 1064 nm laser irradiation including concentration-dependent (i) and power-density-dependent (j) temperature elevation. (k) PCE determination, giving η = 56.5%. (l) Photothermal stability evaluation over multiple laser on/off cycles. (m) Representative thermal imaging with 6 mm chicken breast-covered layer. (n) Infrared thermal images acquired under 808 or 1064 nm laser excitation, in the presence and absence of a chicken layer. Power densities correspond to the MPE-allowed maximum for each wavelength, 808 nm (0.33 W cm^−2^) and 1064 nm (1.0 W cm^−2^). (o) The heating performance of 808 nm and 1064 nm irradiation was compared with and without overlying chicken tissue, highlighting that long-wavelength excitation enables more efficient heat delivery to depth.

**FMR-1105-PEG Micelles for Tumor NIR-II Imaging and Photothermal Performance.** To advance tumor-oriented imaging and PTT, **FMR-1105-PEG** was formulated into DSPE-PEG2000 micelles via solvent evaporation/hydration (Fig. 6a), yielding nanoparticles of ∼133 nm with a narrow size distribution (Fig. 6b). Micellization markedly enhances photon harvesting while preserving the long-wavelength optical window: compared with molecularly dispersed **FMR-1105-PEG** in water (λ_abs_/λ_em_ = 817/1114 nm; MEC = 0.55 × 10^4^ M^−1^ cm^−1^), the micelles maintain λ_abs_/λ_em_ = 815/1090 nm (Fig. S23) and increase the apparent MEC to 10.93 × 10^4^ M^−1^ cm^−1^ (≈20-fold enhancement; Table S1, Fig. S2) [55]. The micellar state remains extremely dim (QY = 0.002%), consistent with a strongly nonradiative-dominant deactivation profile favorable for photothermal operation (Fig. S6). Notably, micellization attenuates but does not abolish microviscosity responsiveness: the NIR-II fluorescence retains a measurable, Förster–Hoffmann-type dependence on viscosity, consistent with nanoconfinement that moderates coupling to microviscosity (Figure S24a-b, S25). This attenuation is likely caused by the confined hydrophobic microenvironment of the micelles, where intramolecular rotation of **FMR-1105-PEG** is partially restricted even when the surrounding bulk medium is of low viscosity. Such pre-restriction can elevate the basal NIR-II fluorescence and reduce the apparent off-on dynamic range compared with the molecularly dispersed rotor. Therefore, for *in vivo* tumor imaging, the micellar formulation may have a weaker viscosity-gated contribution to the SNR than dispersed **FMR-1105-PEG**, especially before clearance of circulating background. Nevertheless, tumor contrast is still supported by micellar tumor accumulation, the low-autofluorescence NIR-II window, while the major advantage of micellization lies in its markedly enhanced photon harvesting and 1064 nm photothermal performance. Accordingly, we adopted a stage-wise strategy: microviscosity-gated NIR-II imaging was used for lesion localization, followed by 1064 nm photothermal ablation using the same fluorophore in a therapeutically optimized micellar formulation.

Using a 4T1 tumor-bearing mouse model, *in vivo* NIR-II fluorescence imaging of **FMR-1105-PEG** micelles revealed clear and time-dependent tumor accumulation after *i.v.* administration (Fig. 6c and S26). Quantitative analysis showed that the tumor fluorescence intensity reached its maximum at 4 h post-injection and then decreased only slowly, remaining relatively stable over 4 - 12 h, indicating sustained tumor retention and a practically wide therapy time window. Importantly, the tumor-to-muscle ratio peaked at 30 h, providing the highest imaging contrast for tumor delineation (Fig. 6d). *Ex vivo* imaging of excised tumors and major organs corroborated the *in vivo* readout and confirmed effective tumor enrichment relative to most normal tissues (Fig. 6e–h, S27-28).

We next evaluated the *in vitro* photothermal performance under 1064 nm irradiation, motivated by improved energy delivery at longer wavelengths for deep-tissue heating. **FMR-1105-PEG** micelles exhibited strong and controllable temperature elevation with concentration- and power-dependent heating behaviors (Fig. 6i–j, S29) and a high photothermal conversion efficiency (PCE) of **η_PT = 56.5%** (Fig. 6k), together with good photothermal stability over repeated laser on/off cycles (Fig. 6l). Additionally, the temperature-elevation profiles of the micelles under identical irradiation conditions were essentially indistinguishable across glycerol/water mixtures spanning various viscosity range (Fig. S24c–d). This viscosity-insensitive heating is consistent with the micellar state remaining strongly nonradiative-dominant, such that viscosity-driven modulation of the radiative branch has minimal impact on the thermal output. Practically, this feature is advantageous for therapy, as it provides a stable photothermal dose while retaining a microviscosity-linked optical signal for imaging. Compared with **ICG**, **FMR-1105-PEG** exhibits more stable viscosity-associated photophysical behavior under biologically relevant conditions, while retaining efficient NIR-II photothermal conversion (Fig. S12 and 30, Table S2), collectively supporting the central “dim-but-hot” concept of **FMR-1105-PEG**. In addition, to assess heat delivery under tissue attenuation, we evaluated the photothermal heating of **FMR-1105-PEG** micelles under 808 and 1064 nm irradiation with a 6 mm chicken-breast layer placed above the sample as a tissue-mimicking barrier (Fig. 6m–o). Photothermal heating was evaluated at each wavelength's maximum permissible exposure (MPE)-allowed maximum power density while maintaining an identical beam spot size (808 nm: 0.33 W cm^−2^; 1064 nm: 1.0 W cm^−2^) [14,64]. Notably, 1064 nm irradiation produced nearly identical heating profiles before and after 6 mm tissue coverage, whereas 808 nm heating was markedly attenuated. This comparison underscores the practical NIR-II advantage that higher MPE-allowed irradiance and more tissue-tolerant heating, and supports **FMR-1105-PEG** micelles as an efficient 1064 nm photothermal formulation enabled by enhanced light harvesting. Collectively, these data establish **FMR-1105-PEG** micelles as a practically deployable NIR-II tumor-imaging agent with sustained tumor uptake and high-contrast delineation, and as an efficient 1064 nm photothermal formulation enabled by markedly enhanced effective light harvesting.

**Fig. 7:**
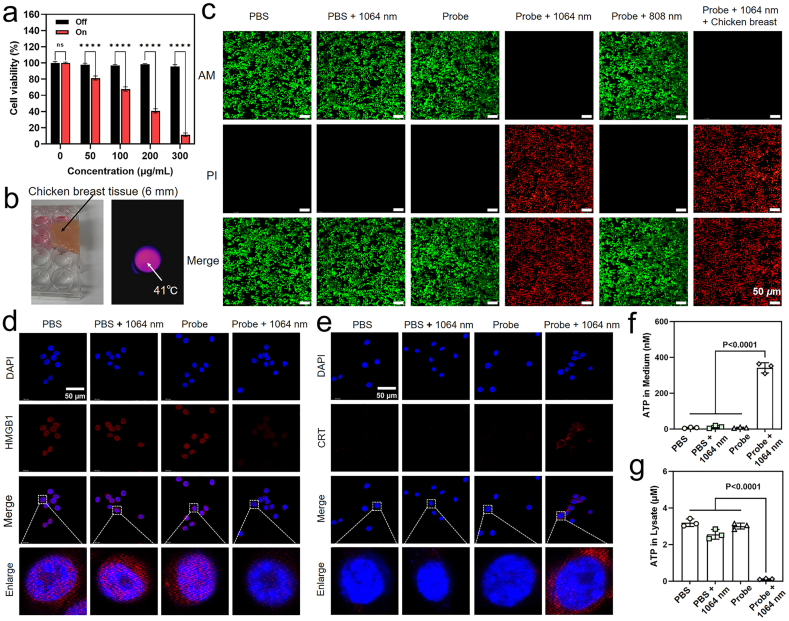
Photothermal cytotoxicity and ICD-associated responses in 4T1 cells. (a) CCK-8 cell viability of 4T1 cells incubated with **FMR-1105-PEG** micelles at the indicated concentrations with or without laser irradiation. (b) Schematic illustration of chicken breast-covered irradiation using a 6 mm chicken breast overlayer. (c) Live/dead staining (calcein-AM/PI) of 4T1 cells after different treatments, including irradiation with 808 or 1064 nm lasers, with and without the tissue overlayer. (d) Immunofluorescence images of HMGB1 showing nuclear-to-cytoplasmic translocation or release after treatment. (e) Immunofluorescence images of CRT exposure on the cell surface. (f) Extracellular ATP levels in culture supernatants after treatment. (g) Intracellular ATP levels in cell lysates after treatment. Nuclei were counterstained with DAPI. Data are presented as mean ± SD (n = 3). Statistical significance: ∗P < 0.05, ∗∗P < 0.01, ∗∗∗P < 0.001, ∗∗∗∗P < 0.0001.

**NIR-II Photothermal Cytotoxicity and ICD Induction in living Cells**. Building on these attributes, we next assessed whether the efficient 1064 nm photothermal performance of **FMR-1105-PEG** micelles could be directed into effective photothermal cytotoxicity in live 4T1 cells. As shown by the CCK-8 assay (Fig. 7a), the micelles exhibited negligible dark cytotoxicity over the tested concentration range, while laser irradiation induced a pronounced, concentration-dependent loss of viability. For subsequent experiments, 4T1 cells were incubated with **FMR-1105-PEG** micelles (300 μg mL^−1^) and irradiated with 808 or 1064 nm lasers for 10 min using an identical beam spot size; a 6 mm chicken breast overlayer was further introduced to mimic tissue-covered conditions (Fig. 7b). As shown in Fig. 7c, live/dead staining exhibited minimal staining changes in the PBS, PBS + laser, and micelle-only controls, whereas micelles +1064 nm led to extensive cell ablation that remained evident even under tissue coverage; in contrast, micelles +808 nm produced negligible killing with or without the overlayer. Beyond direct cytotoxicity, the 1064 nm–triggered treatment elicited hallmark signatures of ICD, including high mobility group protein 1 (HMGB1) release and calreticulin (CRT) exposure (Fig. 7d and e), together with a significant increase in extracellular ATP and depletion of intracellular ATP (Fig. 7f and g), supporting the potential of this NIR-II photothermal modality to couple efficient tumor ablation with immunostimulatory cell death pathways.

**Fig. 8:**
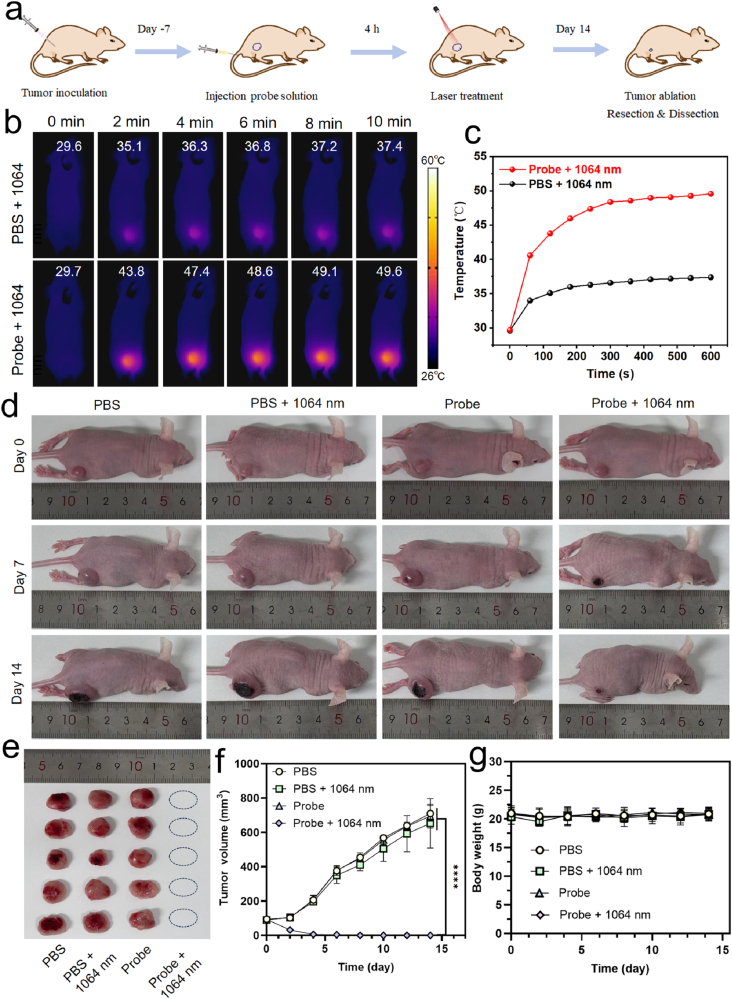
*In vivo* NIR-II PTT using **FMR-1105-PEG** micelles under 1064 nm irradiation. (a) Treatment scheme: tumor inoculation, *i.v.* injection, 1064 nm laser irradiation (1.0 W cm^−2^, 10 min) at 4 h post-injection, and endpoint analysis at Day 14 after PTT. (b) Representative infrared thermal images of tumor-bearing mice during irradiation. (c) Temperature-time profiles at the tumor location. (d) Photographs of 4T1 tumor-bearing mice in different groups at Day 0, 7, and 14. (e) Excised tumors at Day 14. (f) Tumor growth curves (n = 5). (g) Body weight changes during treatment (n = 5). Data are presented as mean ± SD. Statistical significance: ∗∗∗∗P < 0.0001.

**Tumor-Localized NIR-II Photothermal Heating by Probe Drives Efficient Tumor Ablation *In Vivo*.** Building on **FMR-1105-PEG** micelles effectively induces ICD in 4T1 cells, we next sought to determine whether this ICD-prone cytotoxic modality could translate into a therapeutically meaningful outcome *in vivo*. Following *i.v.* administration, Probe enabled efficient NIR-II photothermal activation at the tumor site under 1064 nm irradiation (1.0 W cm^−2^, 10 min, Fig. 8a). Infrared thermography revealed rapid, tumor-localized heating in the probe-treated mice, with the intratumoral temperature rising from ∼29.7 to 49.6 °C within 10 min, whereas the PBS control exhibited only modest warming (29.6 to 37.4 °C), indicating that the temperature elevation was dominated by Probe-mediated photothermal conversion rather than nonspecific laser heating (Fig. 8b and c). Consistent with this pronounced thermal contrast, the combination treatment (Probe +1064 nm) produced a marked therapeutic benefit: serial photographs and endpoint tumor images showed substantial tumor regression/ablation compared with all control groups, and the tumor growth curves (n = 5) demonstrated near-complete suppression of tumor progression through Day 14 with high statistical significance. In contrast, Probe alone and laser alone (PBS + 1064 nm) failed to prevent continuous tumor enlargement, underscoring the requirement for both Probe accumulation and laser irradiation to achieve effective tumor control (Fig. 8d–f). Importantly, body weight remained stable across all groups during the treatment period (n = 5), suggesting good acute tolerability of the therapeutic regimen (Fig. 8g).

**Fig. 9:**
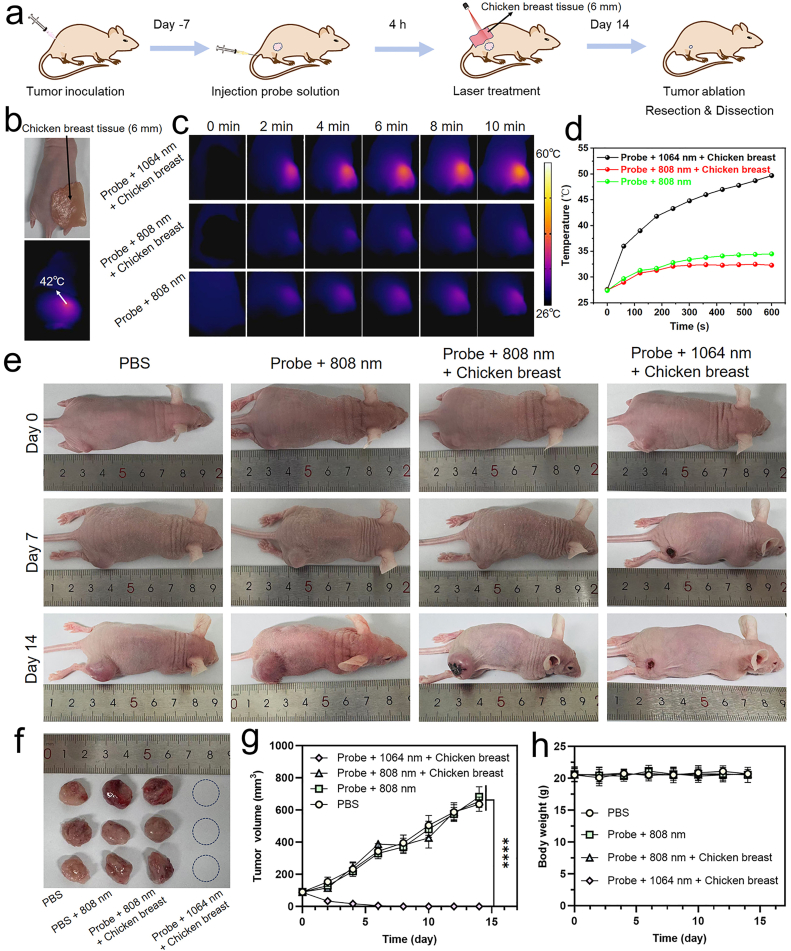
NIR-II excitation affords improved deep-tissue photothermal activation and tumor ablation under MPE-limited irradiation. (a) Treatment scheme. (b) Experimental setup for tissue-coverage evaluation using chicken breast (6 mm) placed over the tumor region. (c) Representative infrared thermal images acquired during irradiation (0 - 10 min) under 808 nm (0.33 W cm^−2^) or 1064 nm (1.0 W cm^−2^), with or without tissue coverage. (d) Temperature-time profiles at the tumor region under the indicated conditions. (e) Representative photographs of mice in different groups at Day 0, 7, and 14, showing the tumor response to varying treatment. (f) Excised tumors at Day 14. (g) Tumor growth curves (n = 3). (h) Body weight changes during treatment (n = 3). Data are presented as mean ± SD. Statistical significance: ∗∗∗∗P < 0.0001.

**Tissue-Barrier Evaluation Validates the Deep-Penetration Benefit of NIR-II Photothermal Activation *in vivo*.** To highlight the deep-tissue advantage of NIR-II excitation, we compared 808 and 1064 nm irradiation using a chicken-breast tissue barrier (6 mm) placed over the tumor region (Fig. 9a and b). Importantly, the two wavelengths were evaluated at their respective MPE-limited power densities (808 nm: 0.33 W cm^−2^; 1064 nm: 1.0 W cm^−2^) with an identical beam spot size, enabling a safety-relevant comparison. Infrared thermography showed that tissue coverage markedly attenuated tumor heating under 808 nm irradiation, whereas 1064 nm irradiation preserved pronounced tumor-localized temperature elevation (Fig. 9c and d). Notably, under the MPE constraint, 808 nm irradiation provided an insufficient thermal dose to yield measurable tumor inhibition even without tissue coverage, while 1064 nm maintained therapeutic performance under a 6 mm tissue barrier. Consistent with the improved deep-tissue photothermal activation, the Probe +1064 nm + chicken-breast group achieved substantially enhanced tumor control over the 14-day observation period relative to the 808 nm counterparts, while body weights remained stable across groups (Fig. 9e–h). These results collectively validate that intravenously administered Probe enables tumor-confined NIR-II photothermal conversion under 1064 nm irradiation, thereby achieving effective tumor control with good acute tolerability.

**Fig. 10:**
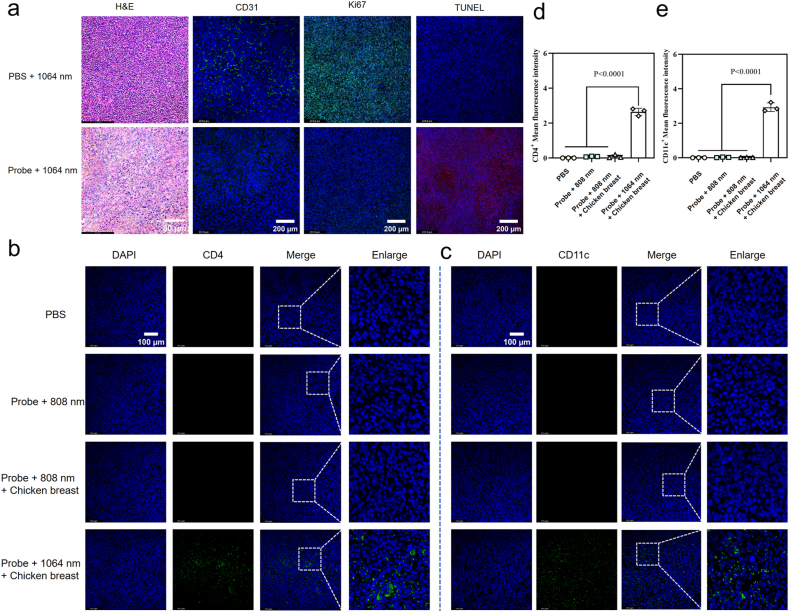
Histological and immunofluorescence analyses of tumor tissues after PTT. (a) Representative H&E staining and immunofluorescence images of CD31, Ki67, and TUNEL in tumor sections from PBS + 1064 nm and Probe +1064 nm groups. (b-c) Representative immunofluorescence images of tumor-infiltrating CD4^+^ T cells (b) and CD11c^+^ cells (c)in PBS, Probe +808 nm, Probe +808 nm + chicken-breast, and Probe +1064 nm + chicken-breast groups. Nuclei were counterstained with DAPI. Scale bars as indicated. Images are representative of independent tumor sections. (d-e) Quantitative analysis of the mean fluorescence intensity of CD4^+^ T cells (d) and CD11c^+^ cells (e). Mean fluorescence intensity was quantified from three randomly selected fields for each group. Data are presented as mean ± SD. Statistical significance: ∗∗∗∗P < 0.0001.

**Immunostaining Suggests Treatment-Associated Immune-Cell Presence in the Tumor Microenvironment.** To further connect the trans-tissue photothermal performance with tissue-level biological outcomes, and in light of the ICD-prone response observed *in vitro*, we analyzed tumor sections by histological and immunofluorescence staining to qualitatively assess cell-death features and immune-cell presence within the tumor microenvironment. As shown in Fig. 10a–S31-32, histological analyses indicated that Probe +1064 nm induced marked tumor injury, as evidenced by obvious pathological alterations in hematoxylin-eosin (H&E) staining, reduced proliferative activity (lower Ki67 signal), and increased DNA fragmentation (enhanced TUNEL staining) relative to PBS + 1064 nm. Immunofluorescence staining further suggested a treatment-associated shift in the local immune contexture: compared with PBS and the 808 nm counterparts, the Probe +1064 nm + chicken-breast group displayed more apparent intratumoral signals of CD4^+^ T cells and CD11c^+^ cells, consistent with enhanced immune-cell infiltration into the tumor bed (Fig. 10b–e). While these observations are qualitative in nature, they collectively support that Probe-mediated NIR-II photothermal injury can elicit immunologically relevant cues in situ; we therefore proceeded to evaluate the systemic biosafety of the treatment *in vivo*.

**Biosafety**. At the Day 14 endpoint, systemic readouts were collected to evaluate the biosafety of the Probe-mediated photothermal regimen. As shown in Fig. S33a, serum biochemistry showed no detectable between-group differences in liver-associated enzymes (ALT. AST, and alkaline phosphatase [ALP]) or renal indices (creatinine [CREA], uric acid [UA], and urea [UREA]) among the PBS, PBS + 1064 nm, Probe, and Probe +1064 nm groups (Fig. S33b–c). Hematological parameters (white blood cell count [WBC], hemoglobin [HGB], mean corpuscular hemoglobin [MCH], and red blood cell count [RBC]) likewise remained comparable across groups (Fig. S33d–g). Consistent with these blood-based assessments, H&E staining of major organs (heart, liver, spleen, lung, and kidney) at Day 14 did not reveal overt treatment-associated pathological alterations (Fig. S33h). Collectively, these Day-14 endpoint evaluations demonstrate good systemic biocompatibility and tolerability of the Probe-mediated photothermal regimen under the current dosing and irradiation conditions.

## 4 Conclusion

We present a microviscosity-gated solution to the fluorescence-heating compromise in a single organic NIR-II fluorophore by redirecting excited-state energy dissipation. Guided by extensive structural design, we built a tunable D-π-A molecular-rotor family in which heteroatom-tailored acceptors and conjugation-length control shifted optical transitions into the NIR-II region while intentionally maintaining low emissive efficiency to favor nonradiative decay. Using a TICT-based rotor architecture, the water-soluble probe **FMR-1105-PEG** delivered a microenvironment-responsive NIR-II output that stayed largely off in low-viscosity media but switched on under viscosity-elevated pathology, enabling lesion identification and longitudinal monitoring; the same mechanism differentiated NAFLD and DILI *in vivo* with strong contrast and supported real-time evaluation of disease status and intervention effects. For tumor therapy, a therapeutically tuned micellar formulation enhanced photon capture and enabled efficient 1064 nm photothermal activation. Although encapsulation weakened viscosity gating, the micelles still showed a measurable Förster-Hoffmann-type viscosity dependence in NIR-II fluorescence, whereas heat generation remained essentially viscosity-independent, consistent with dominant nonradiative relaxation in the confined core. Under 1064 nm irradiation, the formulation produced potent photothermal cytotoxicity and ICD features *in vitro*, achieved localized intratumoral heating and pronounced tumor suppression *in vivo*, and maintained efficacy through a tissue barrier under MPE-relevant conditions, with pathology and immunostaining and systemic safety readouts indicating favorable tolerability. In summary, this work delineates a mechanism-unified theranostic route that couples microviscosity-linked NIR-II reporting with formulation-amplified photothermal ablation, offering transferable design guidelines for microenvironment-coupled NIR-II molecular theranostics.

## CRediT authorship contribution statement

**Yufei Qin:** Data curation, Formal analysis, Investigation, Methodology, Validation. **Yaru You:** Data curation, Investigation, Methodology. **Yishu Yu:** Formal analysis, Investigation. **Yiling Xie:** Investigation. **Yating Sha:** Investigation. **Jinxin Feng:** Investigation. **Yingnan Zeng:** Investigation. **Jiaqi Zhang:** Investigation. **Ziyi Lei:** Investigation. **Caicai Lu:** Investigation, Resources. **Mingxi Fang:** Conceptualization, Funding acquisition, Resources, Supervision, Validation, Writing – review & editing. **Mengchao Cui:** Supervision, Writing – review & editing. **Kaixiang Zhou:** Conceptualization, Funding acquisition, Methodology, Resources, Supervision, Writing – original draft, Writing – review & editing.

## Declaration of competing interest

The authors declare that they have no known competing financial interests or personal relationships that could have appeared to influence the work reported in this paper.

## Data Availability

Data will be made available on request.

## Appendix A Supplementary data

The Supporting Information includes general information; synthetic procedures and characterization; detailed *in vivo* imaging/therapy and cellular protocols; additional figures and tables; supporting data and statistical analysis; and NMR and MS spectra.
