# Supplementary material for: Microviscosity-gated excited-state partitioning in a NIR-II molecular rotor for disease imaging and imaging-guided photothermal therapy
Source: Mater Today Bio. 2026 Jun 10;39:103342. doi: 10.1016/j.mtbio.2026.103342 (PMC13276588; doi:10.1016/j.mtbio.2026.103342)
Supplement: Multimedia component 1 [file mmc1.pdf]

Electronic Supplementary Information (ESI)

Microviscosity-Gated Excited-State Partitioning in a NIR-II Molecular Rotor for Disease Imaging and Imaging-Guided Photothermal Therapy

Yufei Qin[1, 5], Yaru You[2, 5], Yishu Yu[2], Yiling Xie[1], Yating Sha[2], Jinxin Feng[1], Yingnan Zeng[1], Jiaqi Zhang[1], Ziyi Lei[1], Caicai Lu[3], Mingxi Fang[2*], Mengchao Cui[1, 4], Kaixiang Zhou[1, 4*]

[1]Center for Advanced Materials Research & Faculty of Arts and Sciences, Beijing Normal University, Zhuhai 519087, P. R. China

[2]School of Medical Imaging, Xuzhou Medical University, Xuzhou, Jiangsu 221006, P. R. China.

[3]Experimental Teaching Platform, Beijing Normal University, Zhuhai 519087, P. R. China

[4]Key Laboratory of Radiopharmaceuticals, Ministry of Education, College of Chemistry, Beijing Normal University, Beijing 100875, P. R. China

[5]These authors contributed equally

[*]Correspondence: zkx@bnu.edu.cn, mfang@xzhmu.edu.cn

Table of Contents

General Information....................3

Chemistry....................4

In vivo Imaging Methods....................10

Molar Extinction Coefficients....................16

Quantum yields calculations....................18

Theoretical Calculations....................21

Viscosity Sensitivity Test....................24

Penetration Depth Experiment....................30

NIR-II Imaging of the Non-Alcoholic Fatty Liver Mice Model....................31

NIR-II Imaging of the Acute Liver Injury Mice Model....................35

NIR-II Imaging of 4T1 Tumor-bearing Mice....................38

Photothermal Performance and Photothermal Conversion Efficiency Calculations....................43

Biological Safety Evaluation....................46

NMR and MS Spectra....................47

References....................56

General Information

All solvents and chemicals were purchased from commercial products and used without further purification unless otherwise stated. Reactions were monitored by thin layer chromatography: Silica gel 60 F_{254} plates (Merck). Flash column chromatography was carried out on a Oriend a Medium Pressure Preparative Liquid Chromatography system (Mega series, China) with Santai SEPAFLASH silica gel column (40 - 63 μm, 25/40/80 g, China). ^{1}H NMR and ^{13}C NMR spectra were recorded on a Bruker Avance III (400 MHz or 100 MHz) NMR spectrometer in DMSO-d_6 solutions at room temperature. Chemical shift (δ) is reported in ppm downfield from tetramethylsilane and coupling constants (J) are reported in Hertz (Hz), and the multiplicity is defined by s (singlet), d (doublet), t (triplet), or m (multiplet). High-resolution mass spectra (HRMS) were recorded on Thermo scientific Q-Ecactive (ESI) mass spectrometer (USA). Quartz cuvettes (1 cm) were used for absorbance and emission measurements. UV-visible spectra were carried out on the SHIMADZU UV-Vis spectrophotometer (UV-3600i Plus, Japan). Molar absorptivity of all the dyes were determined by linear regression of the absorbance *vs* the concentration of the dyes' solution. Fluorescence spectra were measured on an Edinburgh Steady State and Transient State Fluorescence Spectrometer (FLS1000, UK). Ultrapure water (over 18 MΩ·cm) from a Milli-Q reference system (Millipore) was used throughout. Cell fluorescence images were acquired using a confocal laser scanning microscope (LSM 800) equipped with Zeiss Airyscan. Cell viability was evaluated using a CCK-8 assay kit, and the absorbance of each sample was measured at 450 nm with a microplate reader (SpectraMax i3X, Thermo Fisher Scientific, USA). The ATP assay kit was purchased from Beyotime Biotechnology Co., Ltd. Photothermal experiments were performed with a 1064 nm infrared semiconductor laser

(Changchun New Industries Optoelectronics Technology Co., Ltd.). Temperature variations and photothermal images were recorded using a FLIR thermal imaging camera.

Chemistry

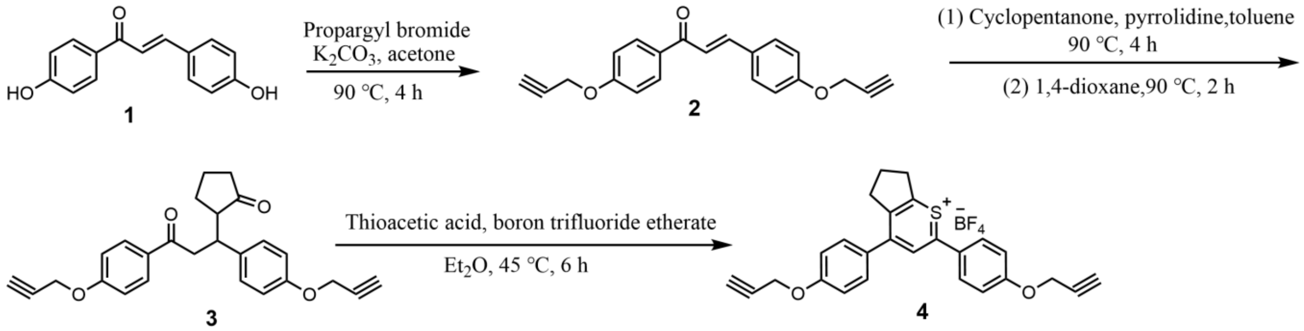

Scheme S1. Synthetic route for Intermediate **4**.

Synthesis of Intermediate **2**

A mixture of intermediate **1** (2.4 g, 10.0 mmol), 3-bromopropyne (3.6 g, 30.0 mmol), and K_2CO_3 (4.1 g, 30.0 mmol) in acetone (140 mL) was refluxed at 90 °C for 5 h under a nitrogen atmosphere. After cooling to room temperature, the reaction mixture was filtered and the filtrate was concentrated under reduced pressure. The residue was treated with a dichloromethane/petroleum ether mixture to induce precipitation. The resulting solid was collected by filtration and dried to afford intermediate **2** as a yellow solid (2.56 g, 88.8%). ^{1}H NMR (400 MHz, DMSO-d_6) δ 8.18 (d, J = 8.8 Hz, 2H), 7.89 (s, 1H), 7.85 (d, J = 15.0 Hz, 2H), 7.70 (d, J = 15.5 Hz, 1H), 7.15 (d, J = 8.8 Hz, 2H), 7.08 (d, J = 8.7 Hz, 2H), 4.95 (d, J = 2.2 Hz, 2H), 4.90 (d, J = 2.2 Hz, 2H), 3.66 (t, J = 2.2 Hz, 1H), 3.63 (t, J = 2.2 Hz, 1H). HRMS: m/z calcd for $C_{21}H_{17}O_3$ 317.1172; found 317.1170, $[M+H]^+$.

Synthesis of Intermediate **3**

Cyclopentanone (644.9 mg, 7.7 mmol) and tetrahydropyrrole (568.2 mg, 7.9 mmol) were dissolved in

toluene (20 mL), and the mixture was refluxed at 90 °C for 4 h under nitrogen. After removal of the solvent under reduced pressure, the residue was dissolved in 1,4-dioxane, followed by addition of intermediate **2** (1.15 g, 3.6 mmol). The reaction mixture was further refluxed at 90 °C for 2 h. After cooling in an ice–water bath, the reaction was quenched with water and extracted with CH_2Cl_2 (3 × 50 mL). The combined organic layers were dried over anhydrous Na_2SO_4, filtered, and concentrated. The crude product was purified by silica gel column chromatography (hexane/CH_2Cl_2/EtOAc = 20:5:1) to afford intermediate **3** as a brownish-yellow oily solid (595.1 mg, 41.14%).^{1}H NMR (400 MHz, DMSO-d_6) δ 7.95 (d, J = 8.8 Hz, 2H), 7.15 (d, J = 8.5 Hz, 2H), 7.07 (d, J = 8.8 Hz, 2H), 6.85 (dd, J = 8.6, 3.5 Hz, 2H), 4.91 (d, J = 2.2 Hz, 2H), 4.73 (d, J = 2.1 Hz, 2H), 3.74 – 3.56 (m, 3H), 3.55 (t, J = 2.2 Hz, 1H), 3.52 – 3.40 (m, 1H), 2.49 – 2.35 (m, 2H), 2.19 – 2.13 (m, 1H), 2.08 –2.00 (m, 1H), 1.96 – 1.78 (m, 2H), 1.73 – 1.61 (m, 2H), 1.43 – 1.39 (m, 1H). HRMS: *m/z* calcd for $C_{26}H_{25}O_4$ 401.1747; found 401.1749, $[M+H]^+$.

Synthesis of Intermediate **4**

Intermediate **3** (595.1 mg, 1.5 mmol) and thioacetic acid (25.3 μL, 3.3 mmol) were dissolved in diethyl ether (20 mL). Boron trifluoride diethyl etherate (1.3 g, 8.9 mmol) was added dropwise under nitrogen, and the reaction mixture was refluxed at 45 °C for 6 h. After cooling to room temperature, the reaction was quenched with water, and excess diethyl ether was added to induce precipitation. The precipitate was collected by filtration, washed with diethyl ether, and dried to give intermediate **4** as a yellow solid (31.3 mg, 43.5%). The resulting solid was used directly in the subsequent reaction without further purification. Caution: Attempts to purify the product by column chromatography were unsuccessful, and the compound could not be isolated. The solid obtained by filtration could be used directly in the subsequent reaction without any issues.

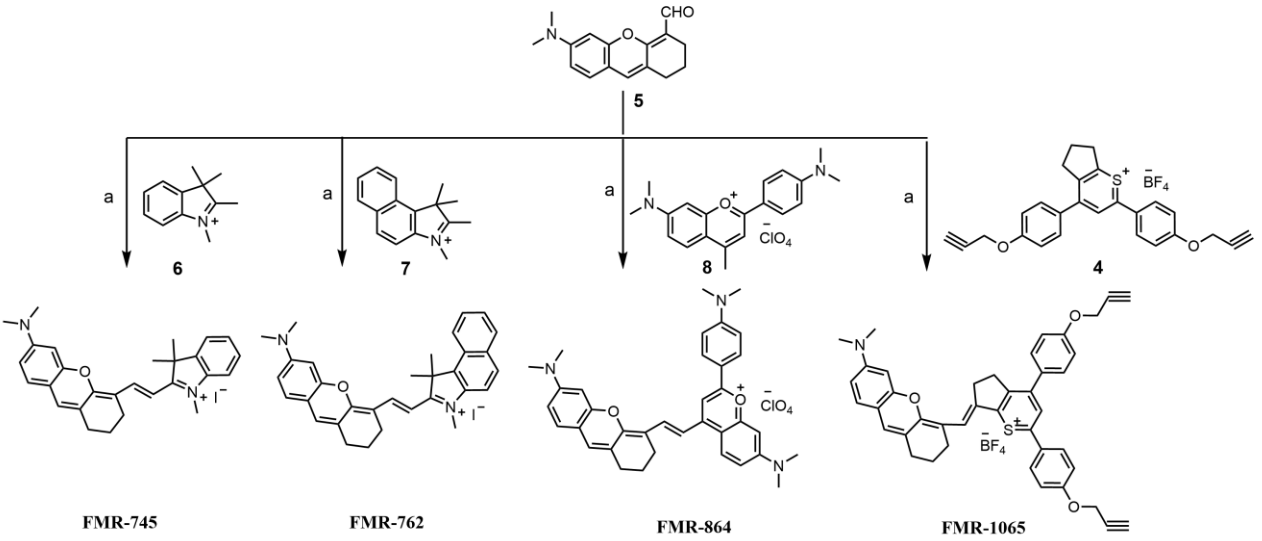

Scheme S2. Synthetic route for **FMRs**. Reagents and conditions: (a) EtOH/ACN, 100 °C, 12 h.

Compound **5** and Compound **9** were synthesized according to the previous literatures.[1]

The synthesis of compound **8**.

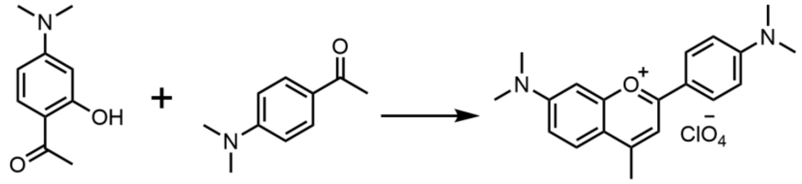

To a solution of -*N*,*N*-dimethylamino-2-hydroxyacetophenone (540 mg, 3 mmol) in acetic acid (15 mL), 1-(4-(dimethylamino)phenyl)ethanone (652 mg, 4 mmol) and perchloric acid (7.5 mL) were added. The reaction mixture was heated under reflux for 13 hours. Upon cooling to ambient temperature, 100 mL of ethyl acetate was added, and the resulting precipitate was isolated by filtration. The solid was washed successively with ethyl acetate and petroleum ether, then dried to yield a red solid (337.8 mg, 29.9%). The product was used directly in the subsequent step without further purification.

Synthesis of **FMR-745**

Intermediate **5** (100.0 mg, 0.39 mmol) and intermediate **6** (176.0 mg, 0.39 mmol) were dissolved in anhydrous acetonitrile/ethanol (v/v = 1:1), and the mixture was refluxed overnight. The reaction

mixture was concentrated, and the crude product was purified by column chromatography (CH_2Cl_2/MeOH = 30:1, v/v) to give the desired product **FMR-745** as a reddish brown solid (63.1 mg, 30%). ^{1}H NMR (400 MHz, DMSO-d_6) δ 8.47 (d, *J* = 14.5 Hz, 1H), 7.67 (d, *J* = 7.4 Hz, 1H), 7.65 (s, 1H), 7.55 – 7.44 (m, 3H), 7.34 (t, *J* = 7.3 Hz, 1H), 6.92 (dd, *J* = 8.9, 2.3 Hz, 1H), 6.67 (d, *J* = 2.1 Hz, 1H), 6.29 (d, *J* = 14.5 Hz, 1H), 3.75 (s, 3H), 3.14 (s, 6H), 2.77 – 2.70 (m, 2H), 2.67 (t, *J* = 5.8 Hz, 2H), 1.88 – 1.79 (m, 2H), 1.74 (s, 6H). ^{13}C NMR (100 MHz, DMSO-d_6) δ 174.05, 161.42, 154.52, 152.98, 142.05, 141.42, 140.55, 136.59, 128.56, 127.96, 124.98, 122.53, 121.81, 113.40, 111.85, 111.39, 111.22, 100.63, 95.55, 48.68, 39.40, 31.22, 27.49, 27.10, 23.25, 19.59. HRMS: *m/z* calcd for $C_{28}H_{31}N_2O^+$ 411.2431; found 411.2426, M^+.

Synthesis of **FMR-762**

Intermediate **5** (100.0 mg, 0.39 mmol) and intermediate **7** (206.0 mg, 0.59 mmol) were dissolved in anhydrous acetonitrile/ethanol (v/v = 1:1), and the mixture was refluxed overnight. The reaction mixture was concentrated, and the crude product was purified by column chromatography (CH_2Cl_2/MeOH = 30:1, v/v) to give the desired product **FMR-762** as a black solid (92.1 mg, 44%). ^{1}H NMR (400 MHz, DMSO-d_6) δ 8.59 (d, *J* = 14.7 Hz, 1H), 8.34 (d, *J* = 8.6 Hz, 1H), 8.15 (d, *J* = 8.9 Hz, 1H), 8.11 (d, *J* = 8.2 Hz, 1H), 7.85 (d, *J* = 8.9 Hz, 1H), 7.70 (t, *J* = 7.7 Hz, 1H), 7.61 (s, 1H), 7.57 (t, *J* = 7.5 Hz, 1H), 7.50 (d, *J* = 8.9 Hz, 1H), 6.91 (dd, *J* = 8.9, 2.4 Hz, 1H), 6.73 (d, *J* = 2.1 Hz, 1H), 6.36 (d, *J* = 14.7 Hz, 1H), 3.89 (s, 3H), 3.17 (s, 6H), 2.79 – 2.65 (m, 6H), 2.00 (s, 6H), 1.89 – 1.80 (m, 2H). ^{13}C NMR (100 MHz, DMSO-d_6) δ 175.78, 161.05, 154.50, 152.92, 140.95, 139.63, 135.93, 133.46, 131.12, 129.77, 129.38, 128.50, 127.23, 126.66, 124.69, 122.68, 121.74, 113.19, 111.74, 111.39, 111.18, 100.60, 95.64, 50.47, 39.43, 31.73, 27.55, 26.64, 23.24, 19.62. HRMS: *m/z* calcd for $C_{33}H_{33}N_2O^+$ 461.2587; found 461.2581, M^+.

Synthesis of **FMR-864**

Intermediate **5** (100.0 mg, 0.39 mmol) and intermediate **8** (160.0 mg, 0.39 mmol) were dissolved in anhydrous acetonitrile/ethanol (v/v = 1:1), and the mixture was refluxed overnight. The reaction mixture was concentrated, and the crude product was purified by column chromatography (CH_2Cl_2/MeOH = 30:1, v/v) to give the desired product **FMR-864** as a black solid (15.9 mg, 6%). ^{1}H NMR (400 MHz, DMSO-d_6) δ 8.45 (d, J = 14.3 Hz, 1H), 8.08 (d, J = 8.8 Hz, 2H), 8.04 (d, J = 9.2 Hz, 1H), 7.67 (s, 1H), 7.29 (d, J = 8.8 Hz, 1H), 7.22 (s, 1H), 6.91 (d, J = 9.3 Hz, 1H), 6.85 – 6.81 (m, 2H), 6.76 (d, J = 8.8 Hz, 3H), 6.73 (dd, J = 8.9, 2.0 Hz, 1H), 3.14 (s, 6H), 3.10 (s, 6H), 3.08 (s, 6H), 2.63 – 2.62 (m, 4H), 1.84 – 1.73 (m, 2H). ^{13}C NMR (100 MHz, DMSO-d_6) δ 159.85, 158.60, 155.12, 154.26, 153.76, 152.34, 150.07, 148.85, 138.46, 132.85, 128.33, 127.74, 125.68, 125.64, 123.20, 115.97, 113.79, 113.57, 112.41, 111.55, 111.53, 111.03, 110.60, 110.33, 110.08, 99.01, 96.74, 96.60, 96.57, 27.78, 23.89, 19.84, 14.40. HRMS: m/z calcd for $C_{36}H_{38}N_3O_2^+$ 544.2958; found 544.2952, M^+.

Synthesis of **FMR-1015**

Intermediate **5** (50.0 mg, 0.19 mmol) and intermediate **4** (136.1 mg, 0.29 mmol) were dissolved in anhydrous acetonitrile/ethanol (v/v = 2:1), and the mixture was refluxed overnight. The reaction mixture was concentrated, and the crude product was purified by column chromatography (CH_2Cl_2/EtOAc = 2:1, v/v) to give the desired product **FMR-1015** as a reddish brown solid (55.0 mg, 40%). ^{1}H NMR (400 MHz, DMSO-d_6) δ 7.92 (d, J = 8.4 Hz, 3H), 7.77 (s, 1H), 7.68 (d, J = 8.6 Hz, 2H), 7.57 – 7.54 (m, 1H), 7.48 (s, 1H), 7.15 (d, J = 8.7 Hz, 4H), 7.04 – 6.98 (m, 2H), 4.93 (dd, J = 5.7, 1.9 Hz, 4H), 3.66 (s, 2H), 3.19 – 3.15 (m, 10H), 2.90 (s, 2H), 2.71 – 2.68 (m, 2H), 1.80 – 1.78 (m, 2H). ^{13}C NMR (100 MHz, DMSO-d_6) δ 162.13, 159.71, 159.62, 158.48, 156.58, 154.93, 144.22, 143.97, 143.11, 139.37, 134.94, 131.64, 131.28, 131.11, 130.52, 130.04, 128.67, 125.72, 124.71, 119.61,

116.12, 115.91, 115.48, 114.77, 113.94, 96.84, 79.50, 79.31, 79.22, 79.10, 56.22, 56.07, 31.12, 31.07, 28.37, 26.81, 21.33, 14.38. HRMS: *m/z* calcd for $C_{42}H_{36}NO_3S^+$ 634.2410; found 634.2402, M^+.

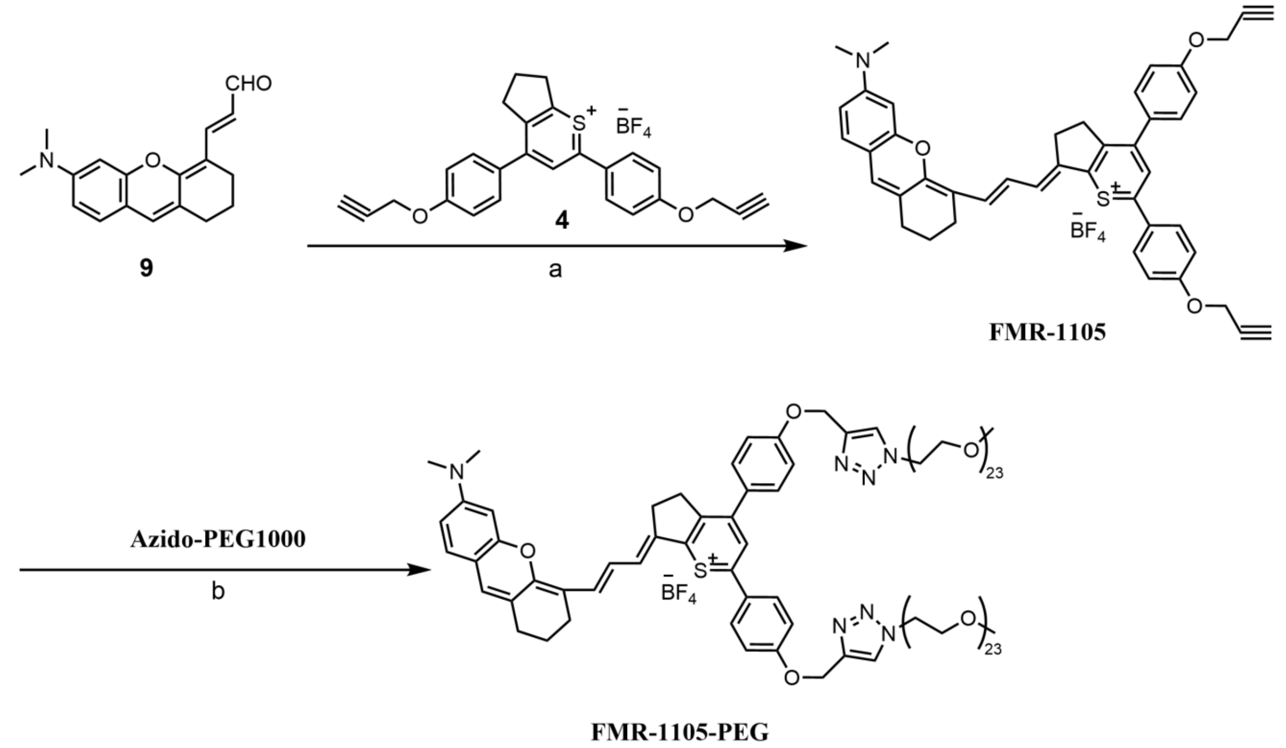

Scheme S3. Synthetic route for **FMR-1105-PEG**. Reagents and conditions: (a) EtOH/ACN, 100 °C, 12 h. (b) $CuSO_4$, sodium ascorbate, DMF/H_2O, r.t., 3 h.

Synthesis of **FMR-1105**

Intermediate **9** (281.3 mg, 0.34 mmol) and intermediate **4** (165.3 mg, 0.34 mmol) were dissolved in anhydrous acetonitrile/ethanol (v/v = 2:1), and the mixture was refluxed overnight. The reaction mixture was concentrated, and the crude product was purified by column chromatography (CH_2Cl_2/EtOAc = 3:1, v/v) to give the desired product **FMR-1015** as a reddish brown solid (161.0 mg, 63%). ^1H NMR (400 MHz, DMSO-d_6) δ 7.80 (s, 1H), 7.71 (d, *J* = 12.6 Hz, 1H), 7.65 (d, *J* = 8.7 Hz, 2H), 7.56 (d, *J* = 8.6 Hz, 2H), 7.43 (d, *J* = 9.1 Hz, 1H), 7.21 – 7.17 (m, 2H), 7.10 (d, *J* = 8.8 Hz, 2H), 7.06 (d, *J* = 8.9 Hz, 2H), 6.90 (dd, *J* = 9.0, 2.0 Hz, 1H), 6.77 (s, 1H), 6.38 (t, *J* = 13.1 Hz, 1H), 4.90 (d, *J* = 2.1 Hz, 2H), 4.86 (d, *J* = 2.1 Hz, 2H), 3.66 (d, *J* = 1.7 Hz, 2H), 3.04 (s, 6H), 2.95 – 2.89 (m, 2H),

2.66 (s, 4H), 2.54 (s, 2H), 1.76 – 1.74 (m, 2H). ^{13}C NMR (100 MHz, DMSO-d_6) δ 160.16, 158.20, 157.01, 155.54, 154.04, 150.84, 143.14, 140.18, 139.56, 139.25, 138.28, 136.77, 134.27, 130.83, 129.13, 127.57, 126.60, 123.34, 122.13, 120.00, 114.83, 114.42, 114.28, 113.34, 95.26, 78.47, 78.21, 78.10, 77.98, 55.07, 54.95, 28.71, 27.50, 27.14, 23.87, 19.75. HRMS: *m/z* calcd for $C_{44}H_{38}NO_3S^+$ 660.2567; found 660.2561, M^+.

Synthesis of **FMR-1105-PEG**

FMR-1105 (27.0 mg, 0.04 mmol) and **Azido-PEG1000** (100.0 mg, 0.10 mmol) were dissolved in DMF (2 mL). To this solution, an aqueous mixture of $CuSO_4·5H_2O$ (24.3 mg, 0.15 mmol) and sodium ascorbate (50.6 mg, 0.25 mmol) in 2 mL of deionized water was added. The resulting reaction mixture was stirred at room temperature for 3 h. The crude product was purified by dialysis and subsequently lyophilized to yield JMR-1002-PEG as a gray solid (97.4 mg, 98.1% yield). The product was further characterized by MALDI-TOF-MS.

In vivo Imaging Methods

Animal Handling. All animal procedures were conducted in strict accordance with the institutional guidelines approved by the Animal Ethics Committee of Beijing Normal University. Female Balb/c nude mice (6 weeks old, 18 - 20 g), female Balb/c mice (6 weeks old, 24 - 28 g) and female KM mice (6 weeks old, 36 - 40 g) were obtained from Zhuhai BesTest Bio-Tech Co., Ltd. and housed under standard laboratory conditions.

During the study, animals were monitored daily for ulceration, impaired mobility, body-weight loss, and other signs of distress. Although dark discoloration of the skin overlying some tumors was observed during rapid tumor expansion, no open wounds, ulceration, bleeding, or infection developed

before the predetermined study endpoint. No animals met the predefined humane endpoint criteria prior to euthanasia.

Preparation of FMR-1105-PEG micelles. Weigh 5 mg of **FMR-1105-PEG** and dissolve it in 3 mL chloroform and 3 mL acetonitrile. Separately, weigh 20 mg of **DSPE-PEG2000** and dissolve it in 6 mL acetonitrile. After mixing the two stock solutions, remove the organic solvents by rotary evaporation under reduced pressure to obtain a lipid thin film. Re-dissolve the film in 10 mL of water and sonicate for 5 min to obtain a clear micellar solution. The solution was then concentrated using a 30 KDa molecular weight cut-off filter (Millipore), centrifuged at 4000 × g for 20 min, and washed with water three times to complete purification.

Establishment of the Acute Liver Injury Mouse Model. To establish an acetaminophen (APAP)-induced acute liver injury model, 6–8-week-old female BALB/c mice were used. Acute liver injury was induced by intraperitoneal injection of varying doses of APAP (dissolved in PBS), while the control group received an equal volume of PBS. Mice were then administered **FMR-1105-PEG** (5 mg/kg, 200 μL) via tail vein injection, followed by NIR-II *in vivo* fluorescence imaging 5 minutes post-injection.

Establishment of the Non-Alcoholic Fatty Liver Mouse Model. KM mice were randomly assigned to three groups: control, NAFL, and NAC + NAFL. The NAFL model was induced by feeding a 60 kcal% high-fat diet and administering daily dexamethasone injections (0.25 mmol/kg/day) for 3 to 9 days. Control mice were fed standard chow and received saline injections of equal volume. The treatment group was given N-acetylcysteine (NAC, 10 mg/kg) by oral gavage every other day while receiving the high-fat diet and dexamethasone injections.

All mice underwent tail vein injection of **FMR-1105-PEG** (5 mg/kg, 200 μL), followed by NIR-II *in*

vivo fluorescence imaging 5 minutes post-injection. At the end of the experiment, major organs were harvested for ex vivo fluorescence imaging, and liver tissues were sectioned at 4 μm for hematoxylin–eosin (HE) staining. The NIR-II imaging parameters were as follows: excitation at 980 nm with a laser power of 74 mW/cm^2, an exposure time of 350 ms, and detection using a 1100 nm LP filter.

Cell Culture. 4T1 murine breast cancer cells were cultured in RPMI-1640 medium supplemented with 10% fetal bovine serum (FBS) and 1% penicillin–streptomycin in a humidified incubator at 37 °C under 5% CO_2. Cells were routinely passaged at 70 – 80% confluence using 0.25% trypsin–EDTA, and only cells in the exponential growth phase were used for subsequent experiments.

Cytotoxicity Evaluation. 4T1 cells were cultured in RPMI-1640 medium supplemented with 10% FBS and 1% penicillin–streptomycin at 37 °C in a humidified incubator with 5% CO_2. Cells were seeded in 96-well plates at 5 × 10^3 cells per well and allowed to adhere for 24 h. The medium was then replaced with fresh medium containing **FMR-1105-PEG** micelles at different concentrations (0, 50, 100, 200, and 300 μg/mL). After incubation for 6 h, cells were irradiated with a 1064 nm laser (1.0 W/cm^2, 10 min); an identical set without laser exposure was included to assess dark cytotoxicity. Cells were further incubated for 18 h, washed three times with PBS, and then treated with serum-free medium containing 10% CCK-8 reagent for 2 h in the dark. Absorbance at 450 nm was recorded using a microplate reader, and cell viability was calculated using the following equation:

$$\text{Cell viability (\%)} = \frac{(OD_{\text{sample}} - OD_{\text{blank}})}{(OD_{\text{control}} - OD_{\text{blank}})} \times 100\%$$

where "OD_{sample}" is the absorbance of **FMR-1105-PEG** micelle–treated cells (± laser), "OD_{control}" is the absorbance of untreated cells, and "OD_{blank}" is the absorbance of blank wells without cells.

Live-Dead Cell Staining. First, 4T1 cells were seeded in 96-well plates and cultured for 24 h to allow adherence. The cells were then subjected to the following treatments: (1) PBS; (2) 1064 nm laser

irradiation for 10 min; (3) incubation with **FMR-1105-PEG** micelles for 6 h; (4) incubation with **FMR-1105-PEG** micelles for 6 h followed by 1064 nm laser irradiation for 10 min; (5) incubation with **FMR-1105-PEG** micelles for 6 h followed by 808 nm laser irradiation for 10 min; and (6) incubation with **FMR-1105-PEG** micelles for 6 h, followed by 1064 nm laser irradiation for 10 min with the wells covered by 6 mm-thick chicken breast tissue. The concentration of **FMR-1105-PEG** micelles was fixed at 300 μg/mL. The power densities were 1.0 W/cm^2 for the 1064 nm laser and 0.33 W/cm^2 for the 808 nm laser. After each treatment, cells were further incubated at 37 °C for 30 min. Cells were subsequently stained with propidium iodide (PI) and calcein AM at a concentration of 60 μg/mL for 10 min, gently rinsed with PBS, and imaged using a confocal laser scanning microscope (CLSM). Imaging parameters were as follows: excitation at 488 nm for calcein AM and 561 nm for PI, with emission collected at 494 – 517 nm and 535 – 617 nm, respectively.

Immunostaining of 4T1 Cells. 4T1 cells were seeded in 3.5 cm confocal dishes at a density of 2 × 10^5 cells in 2 mL of medium per dish and cultured overnight. The PBS, PBS + 1064 nm, Probe, and Probe + 1064 nm groups were incubated with PBS or **FMR-1105-PEG** micelle solution (300 μg/mL) for 6 h, followed by treatment with or without 1064 nm laser irradiation (1 W/cm^2) for 10 min. Cells were then washed with PBS and fixed with 4% paraformaldehyde (PFA) 6 h post-treatment. Subsequently, the cells were incubated with anti-calreticulin (CRT) antibody (Proteintech, 1:200) and anti-HMGB1 antibody (Proteintech, 1:400), followed by an HRP-conjugated anti-rabbit secondary antibody (Proteintech, 1:400). After three washes with PBS, cells were imaged using a confocal laser scanning microscope (CLSM).

ATP Release Assay. 4T1 cells were seeded in confocal dishes and cultured for 24 h. The PBS, PBS + 1064 nm, Probe, and Probe + 1064 nm groups were incubated with PBS or **FMR-1105-PEG** micelle

solution (300 μg/mL) for 6 h, followed by treatment with or without 1064 nm laser irradiation (1 W/cm^2) for 10 min. After 24 h, cell lysates from each group were collected. ATP levels in the medium and lysates were determined using an ATP assay kit according to the manufacturer's instructions.

Establishment of the 4T1 Subcutaneous Tumor-Bearing Nude Mouse Model. 4T1 murine breast cancer cells at the logarithmic growth phase were harvested by trypsinization, collected by centrifugation, and resuspended in sterile PBS. The cell suspension was mixed with Matrigel at a volume ratio of 1:1 to obtain a final concentration of 1×10^6 cells per 100 μL. Female nude mice (6–8 weeks old) were subcutaneously injected with 100 μL of the cell mixture to establish the subcutaneous tumor model. When the tumor volume reached approximately 100 mm^3, subsequent *in vivo* experiments were performed.

***In Vivo* NIR-II Tumor Imaging.** For *in vivo* NIR-II fluorescence imaging, **FMR-1105-PEG** micelles solution was intravenously injected into 4T1 tumor-bearing mice. NIR-II imaging was performed at different time points post-injection under 980 nm laser excitation (power density: 52 mW/cm^2) using an 1100 nm LP filter with an exposure time of 350 ms. The NIR-II images were analyzed using the open-source software ImageJ.

***In Vivo* Photothermal Therapy.** When the tumor volume reached approximately 100 mm^3, mice were randomly assigned to seven groups (n = 3): (1) PBS; (2) PBS + 1064 nm laser irradiation; (3) Probe; (4) Probe + 1064 nm laser irradiation; (5) Probe + 808 nm laser irradiation; (6) Probe + 808 nm laser irradiation with 6 mm-thick chicken breast tissue covering; and (7) Probe + 1064 nm laser irradiation with 6 mm-thick chicken breast tissue covering. Four hours prior to photothermal therapy, PBS (150 μL) or **FMR-1105-PEG** micelle solution (150 μL, 2 mg/mL) was administered via tail-vein injection. Four hours after injection, tumors were irradiated with a 1064 nm laser (1 W/cm^2) or an 808 nm laser

(0.33 W/cm^2). Body weight and tumor volume were monitored throughout the treatment period. Tumor volume was calculated as: tumor volume = 1/2 × (length × width^2).

Immunohistochemistry. After photothermal therapy, immunofluorescence staining was performed on tumor sections from nude mice in the PBS, PBS + 1064 nm, Probe, and Probe + 1064 nm groups. Sections were fixed with 4% paraformaldehyde (PFA) for 10 min and blocked with 10% FBS at room temperature for 1 h. The sections were then incubated with primary antibodies (anti-CD4 and anti-CD11c, Abcam) at 4 °C overnight. Nuclei were counterstained with DAPI, followed by washing with PBS. In addition, Ki67, TUNEL, and CD31 staining was performed on tumor sections from all four groups. Finally, sections were mounted with mounting medium (Sangon, Shanghai) and imaged using a confocal microscope (Leica).

Statistical analysis. All experimental data are presented as the mean ± standard deviation (SD). The non-paired T-test was used for comparison between the two groups, and one-way analysis of variance (ANOVA) was used for comparison among multiple groups (* indicates statistically significant difference; * $P < 0.05$, ** $P < 0.01$, *** $P < 0.001$, **** $P < 0.0001$).

Molar Extinction Coefficients.

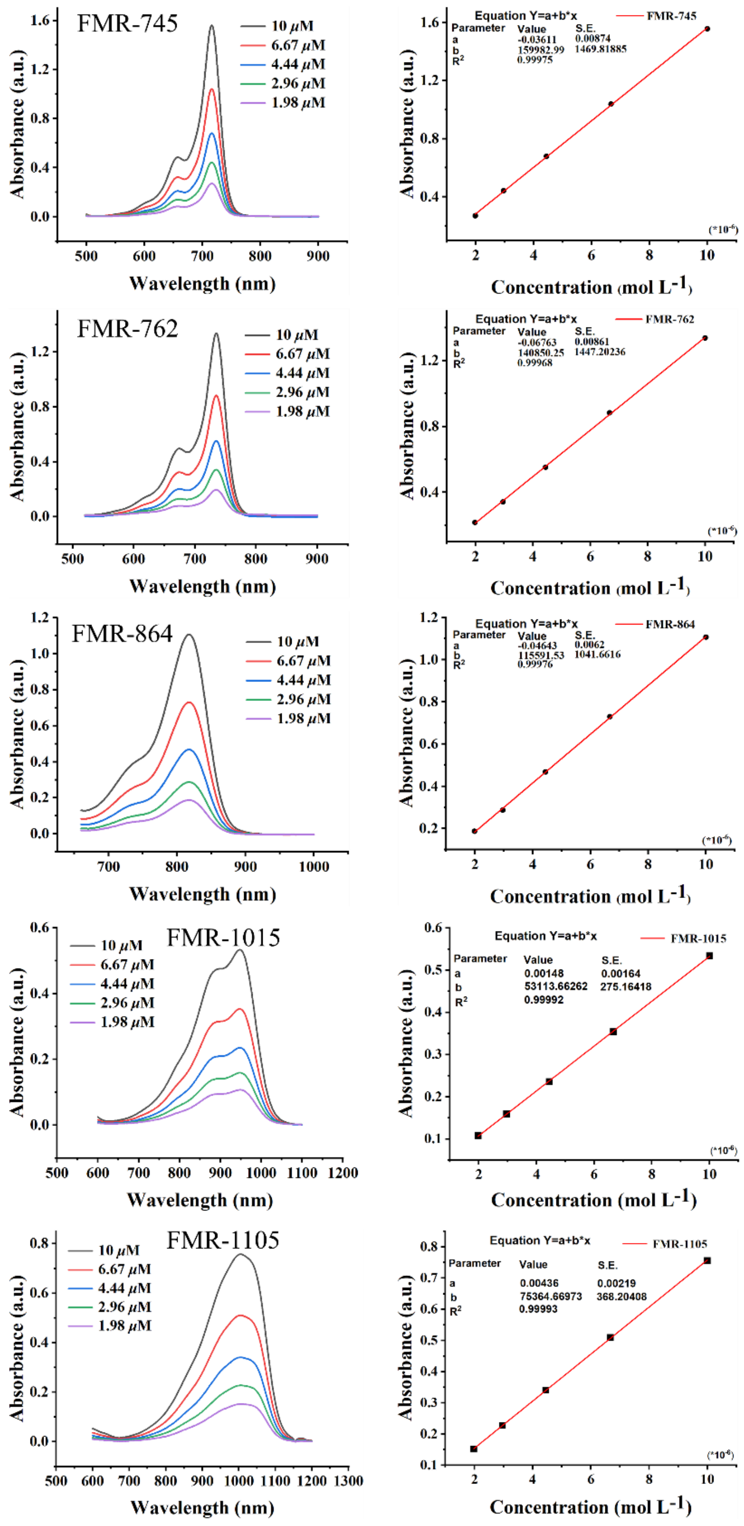

Figure S1. The left panels show the UV–vis absorption spectra of **FMR-745**, **FMR-762**, **FMR-864**, **FMR-1015**, and **FMR-1105** in dichloromethane at various concentrations. The right panels show the corresponding linear regressions of absorbance versus concentration for each probe; the fitted slope (b) corresponds to the molar extinction coefficient (ε).

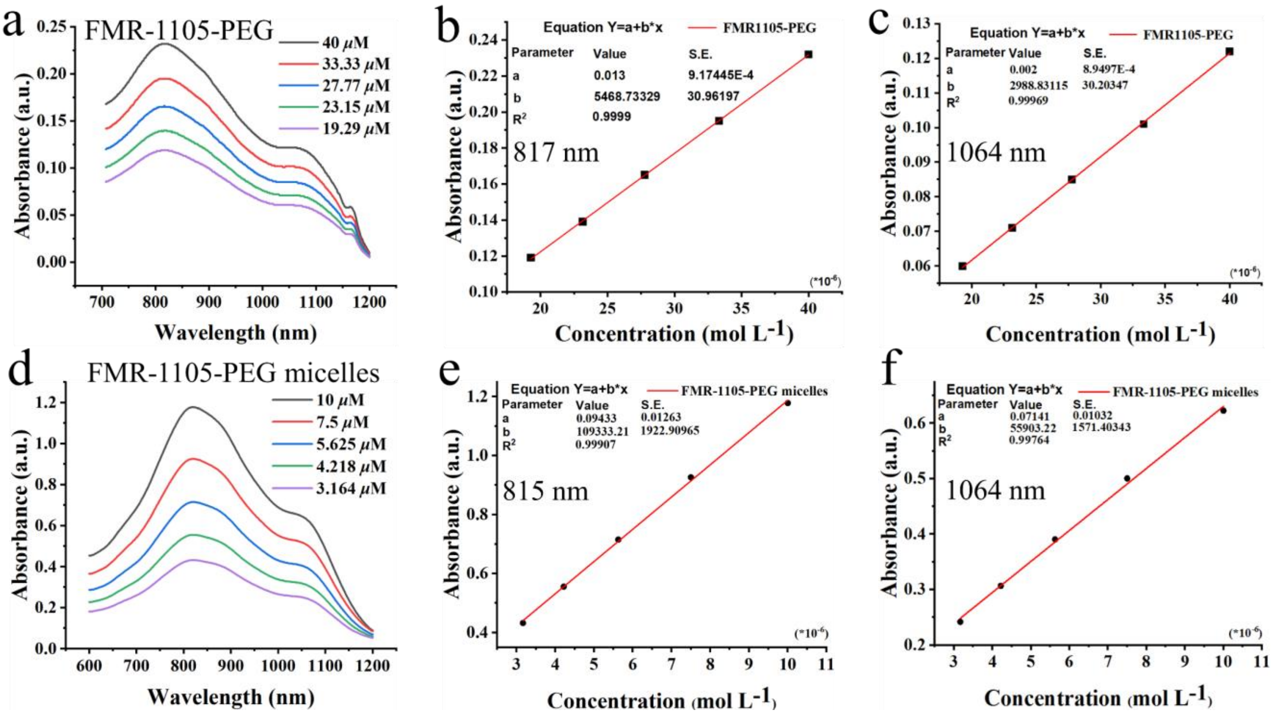

Figure S2. UV–vis absorption spectra of **FMR-1105-PEG** (a) and **FMR-1105-PEG** micelles (d) in water at different concentrations. (b,e) Linear regressions of the peak absorbance versus concentration for each probe. (c,f) Linear regressions of the absorbance at 1064 nm versus concentration for each probe. The fitted slope in (b) corresponds to the molar extinction coefficient (ε).

Table S1. Photophysical parameters of **FMRs**.

Probe	$\lambda_{max, abs}$ (nm)	$\lambda_{max, em}$ (nm)	Stokes shift (nm)	QYs[a] (%)	MEC (ε) (M^{-1} cm^{-1}) ×10^4	Brightness[b] (M^{-1} cm^{-1})
**FMR-745**	716	745	29	8.67	16.00	13862
**FMR-762**	735	762	27	20.67	14.09	29118
**FMR-864**	817	864	47	2.80	11.56	3234
**FMR-1015**	948	1015	67	0.20	5.31	108
**FMR-1105**	1007	1105	98	0.06	7.54	44
**FMR-1105-PEG**	817	1114	297	n.d.	0.55[c]/0.30[d]	n.d.
**FMR-1105-PEG micelles**	815	1090	275	0.002	10.93[c]/5.59[c]	2

[a]The QYs of **FMR-745** and **FMR-762** were determined using **ICG** (13% in dimethyl sulfoxide) as the reference, whereas those of the other probes were determined using **IR26** (0.05% in dichloroethane) as the reference. [b]Brightness equals the product of fluorescence quantum yield and molar extinction coefficient. [c]Apparent molar extinction coefficient. [d]The molar extinction coefficient at 1064 nm.

Quantum yields calculations.

For **FMR-745** and **FMR-762**, **ICG** in DMSO was used as the reference (Φ = 13%). The peak wavelength in the UV–vis absorption spectrum was selected as the excitation wavelength, and the emission spectra were collected over 700 - 1300 nm. For **FMR-864**, **FMR-1015**, **FMR-1105**, **FMR-1105-PEG**, and **FMR-1105-PEG** micelles, **IR-26** in 1,2-dichloroethane was used as the reference (Φ = 0.05%). An 808 nm laser was used as the excitation source, and the emission spectra were collected over 850–1600 nm. For all dye solutions, the absorbance at the excitation wavelength was kept below 0.1 to maximize illumination homogeneity and optical transparency. Integrate the emission spectra to obtain the total emission intensity. The quantum yield of the sample was calculated using the following equation:

$$\Phi_{sample} = \Phi_{ref} \times \frac{Slope_{sample}}{Slope_{ref}} \times \left(\frac{n_{sample}}{n_{ref}}\right)^2$$

where the subscripts “sample” and “ref” denote the sample and the reference standard, respectively; “Slope” is the slope obtained from the linear fit of the integrated fluorescence intensity versus absorbance; and “n” is the refractive index of the solvent.

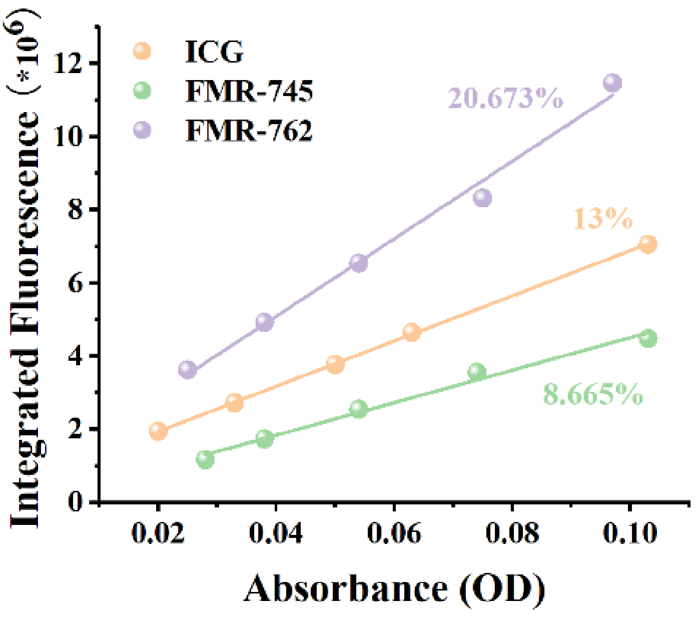

Figure S3. Integrated fluorescence intensity versus absorbance for **ICG**, **FMR-745** and **FMR-762**. **ICG** in DMSO was used as the reference (Φ = 13%), and the relative quantum yields (QYs) were determined from the slopes of the linear fits.

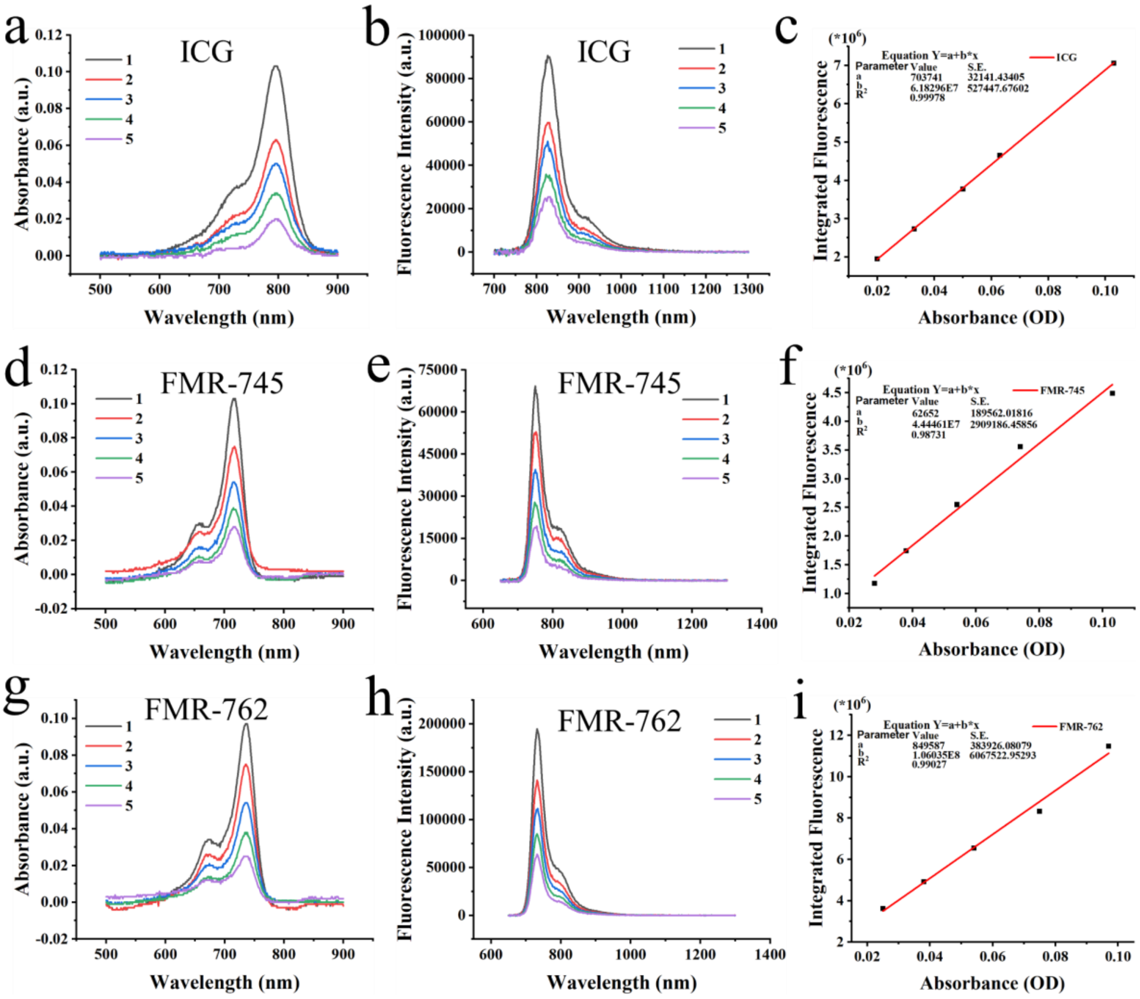

Figure S4. Quantum yields measurement of **FMR-745** and **FMR-762** in CH_2Cl_2. The absorption spectra of **ICG** (a), **FMR-745** (d) and **FMR-762** (g) at five concentrations, with all absorbance values maintained below 0.1. The corresponding fluorescent emission profiles of **ICG** (b), **FMR-745** (e) and **FMR-762** (h) after absorption measurements. The linear fitting curve between absorbance and fluorescence integral intensity of **ICG** (c), **FMR-745** (f) and **FMR-762** (i).

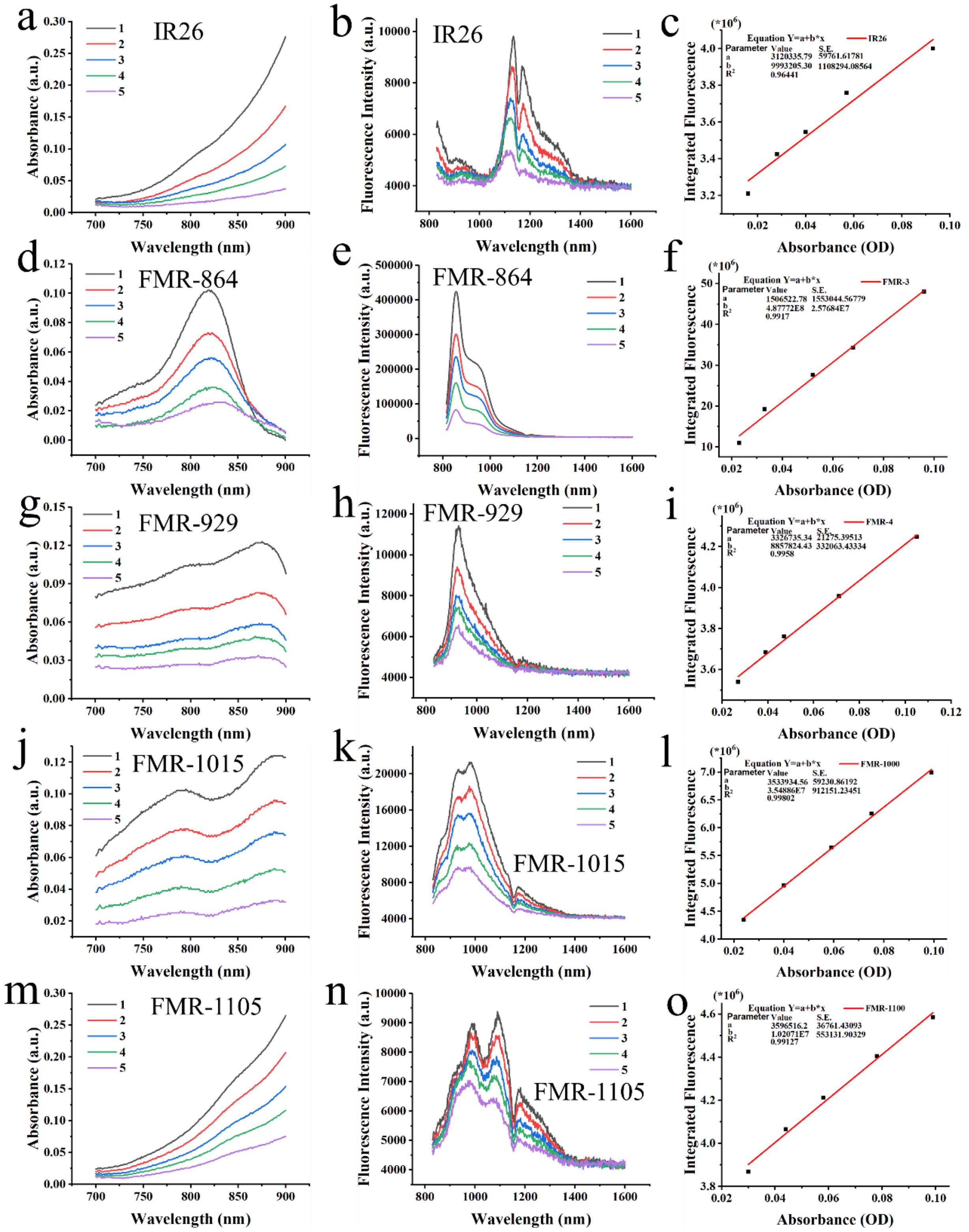

Figure S5. Quantum yields measurement of **FMR-864**, **FMR-929**, **FMR-1015** and **FMR-1105** in CH_2Cl_2. The absorption spectrum of **IR26** (a), **FMR-864** (d), **FMR-929** (g), **FMR-1015** (j) and **FMR-1105** (m) around 808 nm with five concentrations, and the absorption values were maintained below 0.1 at 808 nm. The corresponding fluorescent emission profiles of **IR26** (b), **FMR-864** (e), **FMR-929** (h), **FMR-1015** (k) and **FMR-1105** (n) after absorption measurements. The linear fitting curve between absorbance and fluorescence integral intensity of **IR26** (c), **FMR-864** (f), **FMR-929** (i), **FMR-1015** (l) and **FMR-1105** (o) in CH_2Cl_2.

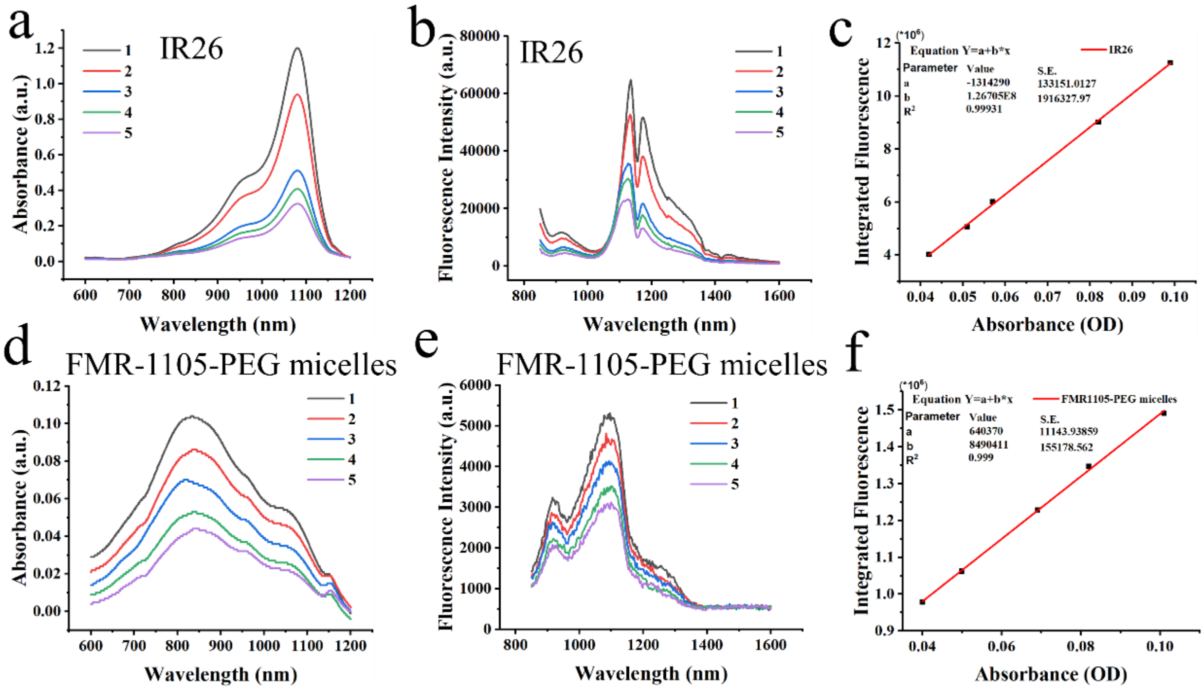

Figure S6. Quantum yields measurement of **FMR-1105-PEG** micelles in water. The absorption spectrum of **IR26** (a) and **FMR-1105-PEG** micelles (d) around 808 nm with five concentrations, and the absorption values were maintained below 0.1 at 808 nm. The corresponding fluorescent emission profiles of **IR26** (b) and **FMR-1105-PEG** micelles (e) after absorption measurements. The linear fitting curve between absorbance and fluorescence integral intensity of **IR26** (c) and **FMR-1105-PEG** micelles (f).

Theoretical Calculations.

All quantum chemical calculations were performed using Gaussian 16. First, geometry optimizations were carried out based on density functional theory (DFT) using the B3LYP hybrid functional with the 6-31G basis set until the lowest-energy stable conformations were obtained, thereby ensuring the reliability of the optimized molecular structures for subsequent calculations. Subsequently, single-point energy calculations were performed at the B3LYP/6-31G(d) level to obtain information on the electronic structures and molecular orbitals. On this basis, the singlet excited states of **FMR-745**, **FMR-762**, **FMR-864**, **FMR-1015**, and **FMR-1105** were calculated using time-dependent density functional theory (TD-DFT). Natural transition orbital (NTO) analysis was further conducted to

evaluate the contribution of each excited state to the electronic transition process. The NTO analysis revealed that the first excited state made the predominant contribution to the electronic transition.

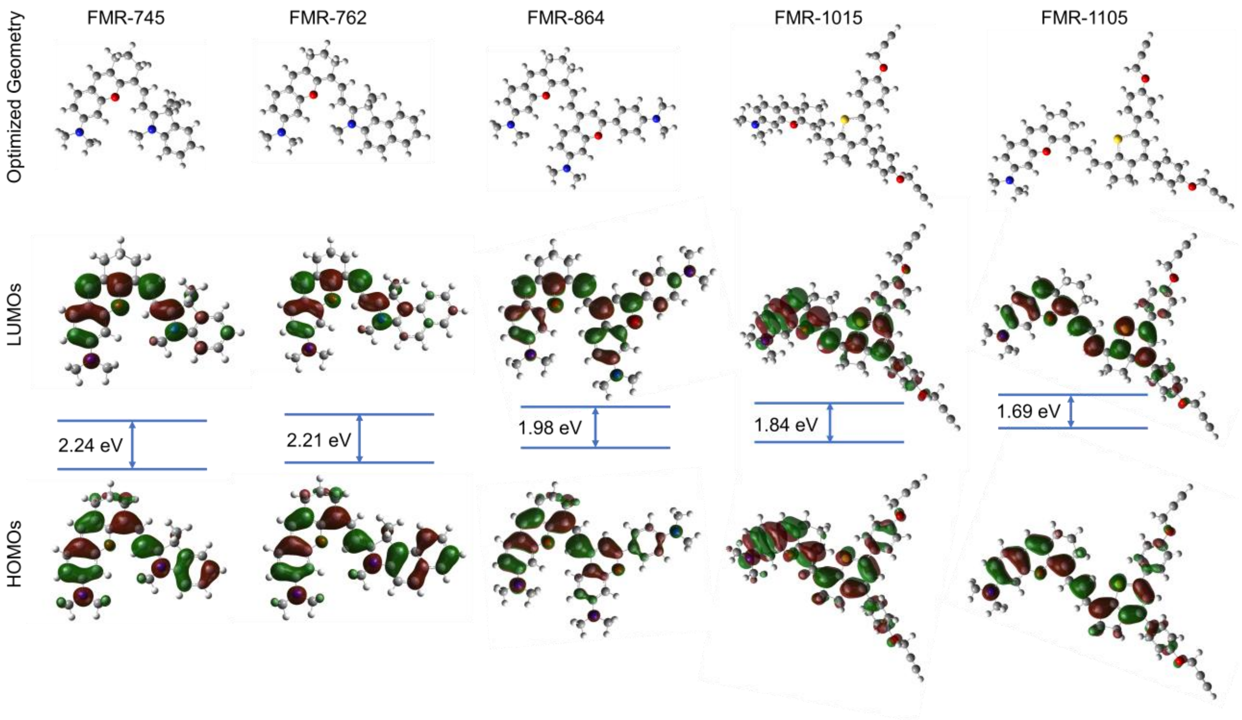

Figure S7. DFT-optimized geometries and frontier molecular orbital distributions of **FMR** family. The calculated HOMO-LUMO gaps decrease from 2.24 to 1.69 eV, supporting bandgap narrowing induced by acceptor heteroatom modulation and π-conjugation extension.

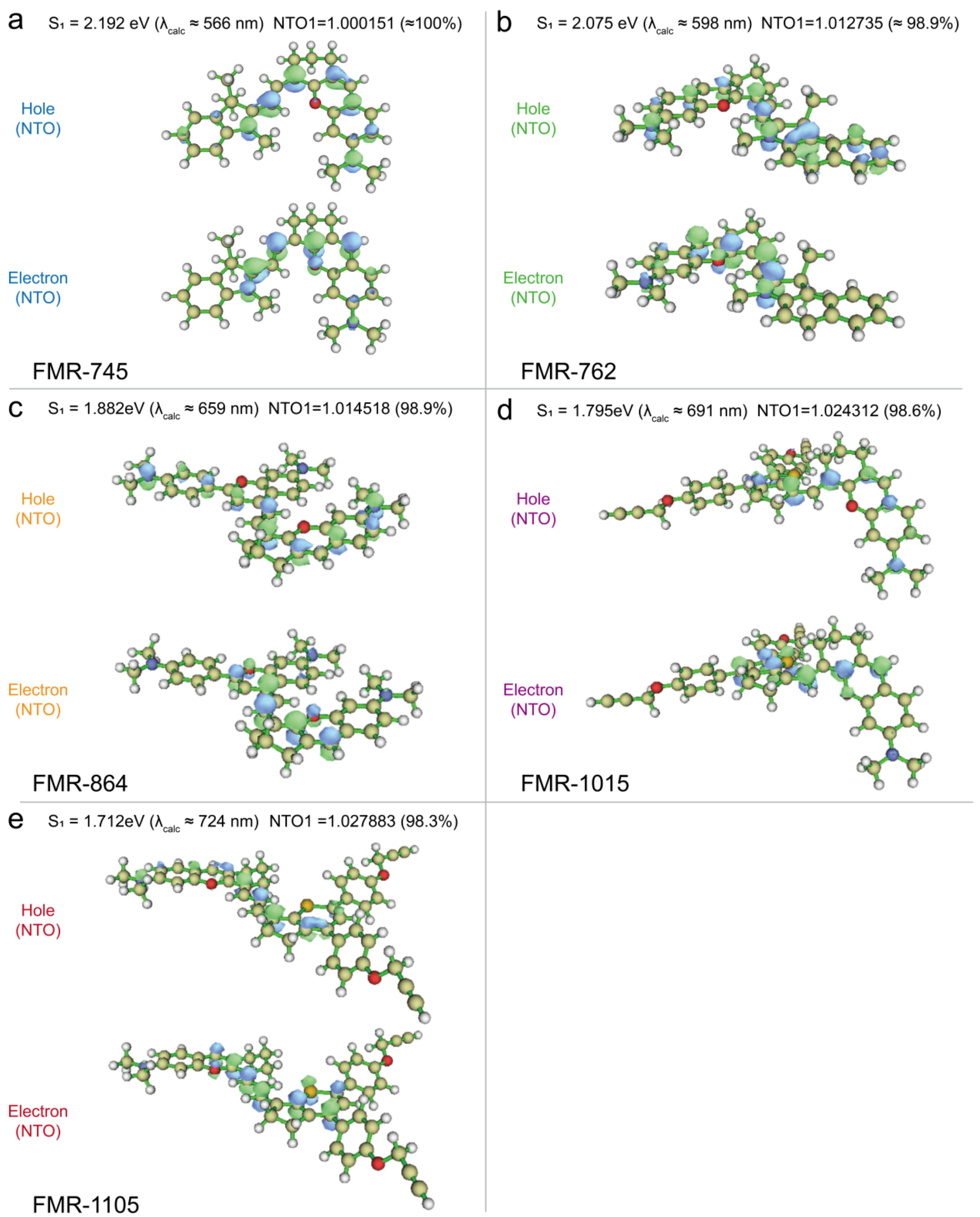

Figure S8. NTO analysis for the S_1 state of dyes including **FMR-745**, **FMR-762**, **FMR-864**, **FMR-1015**, and **FMR-1105**. For each molecule, the spatial distributions of the highest occupied NTO (Hole, top) and the lowest unoccupied NTO (Electron, bottom) are illustrated. The calculated vertical excitation energy (S_1), maximum absorption wavelength (λ_{max}), and the eigenvalue contribution percentage of the primary NTO pair (NTO1) are annotated accordingly.

Viscosity Sensitivity Test.

To evaluate the viscosity responsiveness of the fluorescent molecular rotor probe **FMRs**, a series of water/glycerol mixtures were used to provide media with different viscosities. A series of water/glycerol mixtures with varying glycerol volume fractions ($f_{glycerol}$ = 0, 10, 20, 30, 40, 50, 60, 70, 80, 90, and 100 vol%) were prepared. A stock solution of **FMRs** was then added to each mixture to give a final probe concentration of 10 μM. After thorough mixing, the samples were allowed to equilibrate at room temperature prior to fluorescence measurements. Fluorescence emission spectra were collected under identical instrumental settings to ensure data comparability across the viscosity series. The relationship between fluorescence intensity and viscosity was analyzed by linear fitting according to the Förster–Hoffmann equation.

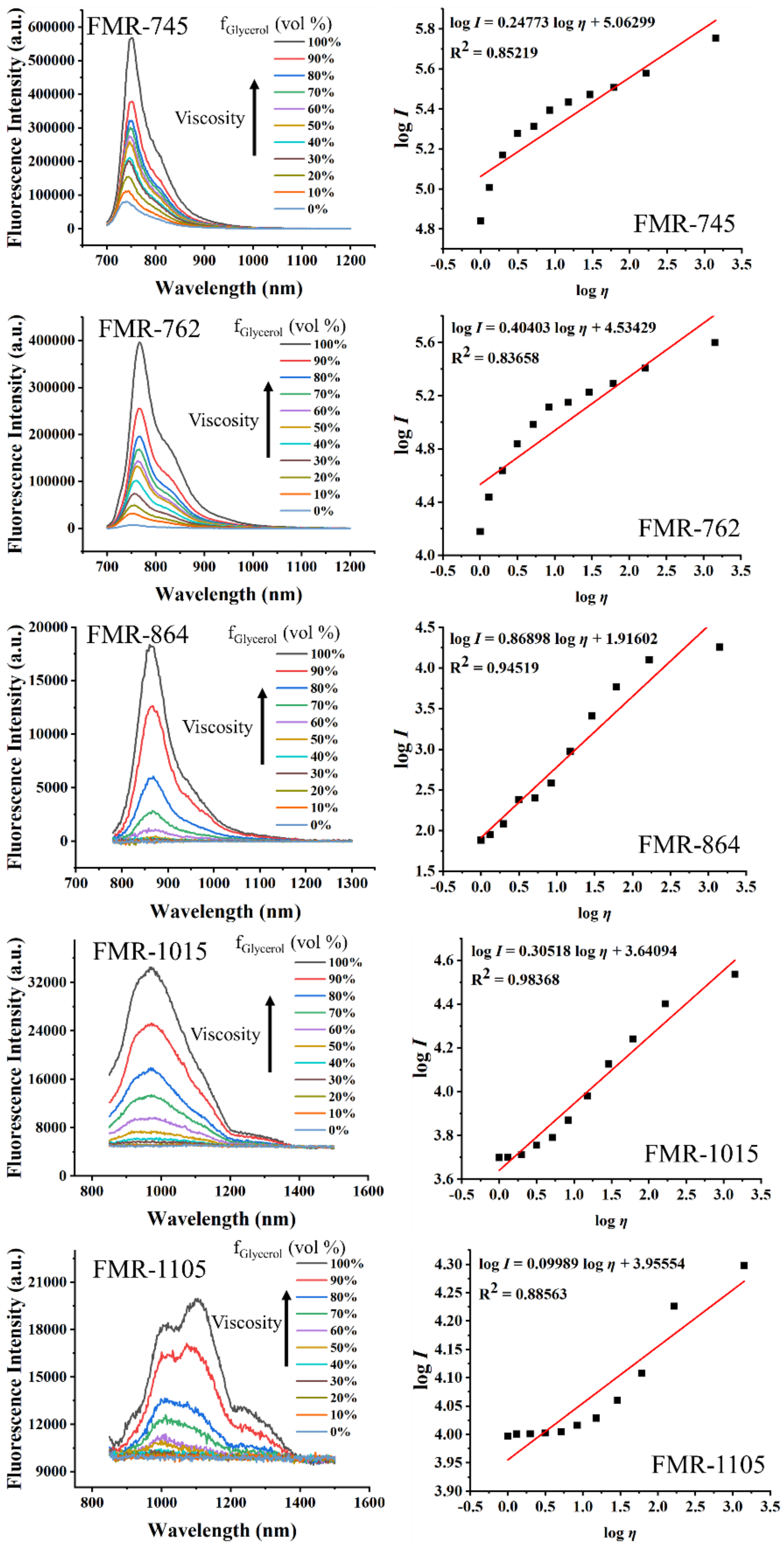

Figure S9. The viscosity was adjusted by mixing water and glycerol at different volume ratios, and the fluorescence emission spectra of **FMRs** were recorded at the corresponding viscosities. The left panel shows the fluorescence emission spectra of the probe, and the right panel shows the viscosity calibration curve obtained from a log–log plot of fluorescence intensity versus viscosity.

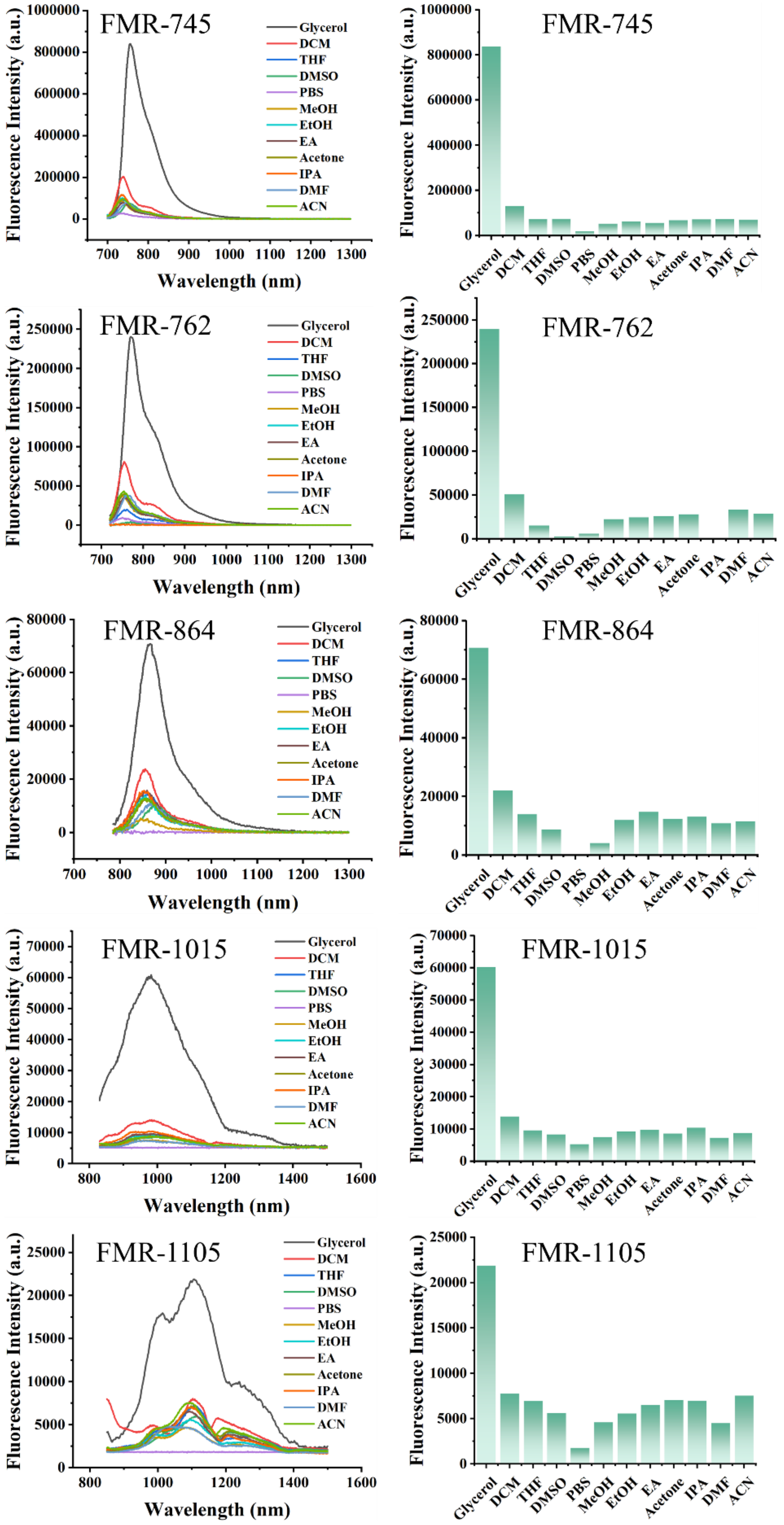

Figure S10. Fluorescence responses of **FMRs** were measured in solvents with different polarities. The left panel shows the fluorescence emission spectra of the probes, and the right panel presents the quantitative analysis of the corresponding fluorescence intensities in these solvents. Dyes concentration: 10 μM.

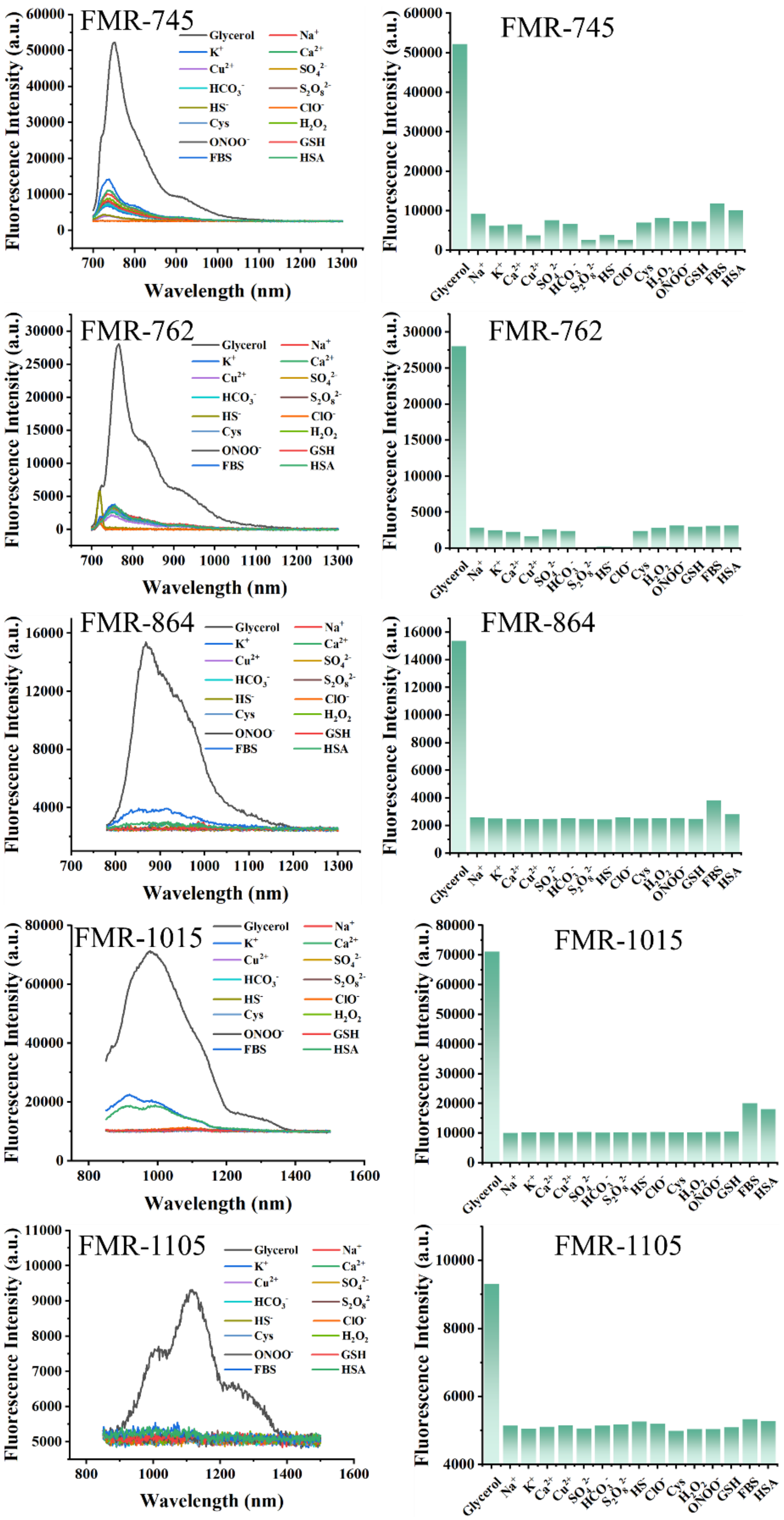

Figure S11. Fluorescence responses of **FMRs** in glycerol and in phosphate-buffered saline (PBS) containing potential interfering species. The left panel shows the emission spectra of the probe (with the added interfering species indicated in the figure). The right panel shows the corresponding fluorescence intensities of the probe in glycerol and in PBS containing the interfering species. Dye concentration: 10 μM.

Table S2. Comparison of PCE and Viscosity Sensitivity

Probe	PCE (%)	Viscosity sensitivity
**ICG**	69.1	0.15241
**FMR-1105-PEG**	n.d.	0.08076
**FMR-1105-PEG micelles**	56.5	0.04204

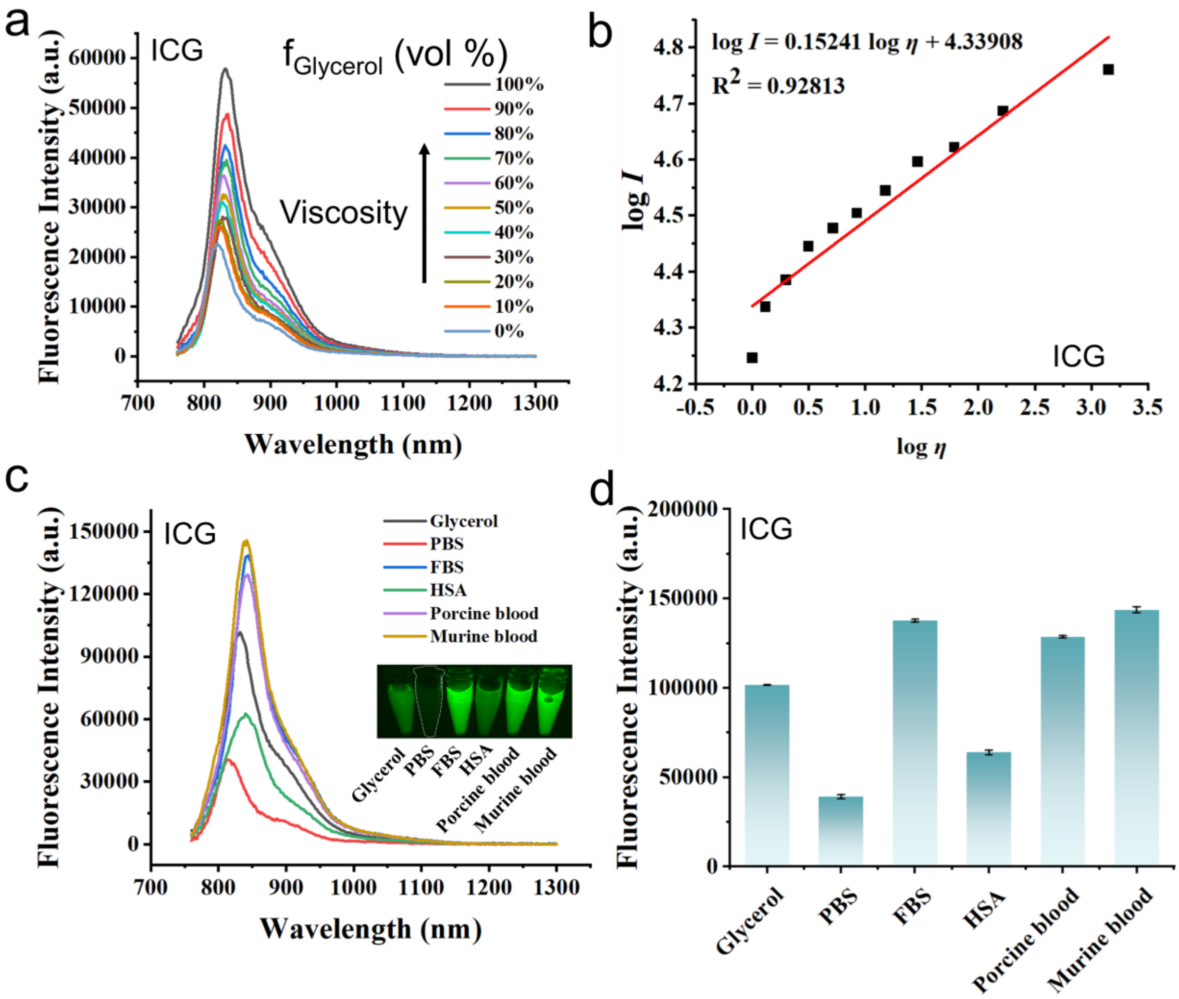

Figure S12. Fluorescence responsive properties and selectivity evaluation of **ICG**. (a) Fluorescence spectra of **ICG** in glycerol-water mixtures with varying volume fractions (0-100%), showing viscosity-dependent intensity enhancement. (b) Correlation between logarithm of fluorescence intensity and logarithm of viscosity for **ICG**, with fitted line and regression equation. (c) Fluorescence spectra of **ICG** in different biological media, including glycerol, PBS, FBS, HSA, porcine blood, and murine blood; inset shows corresponding NIR-II fluorescence images. **ICG** shows stronger fluorescence in all biological media compared to PBS, indicating that interactions with proteins and biomolecules enhance emission. (d) Quantitative comparison of fluorescence intensities from panel (c).

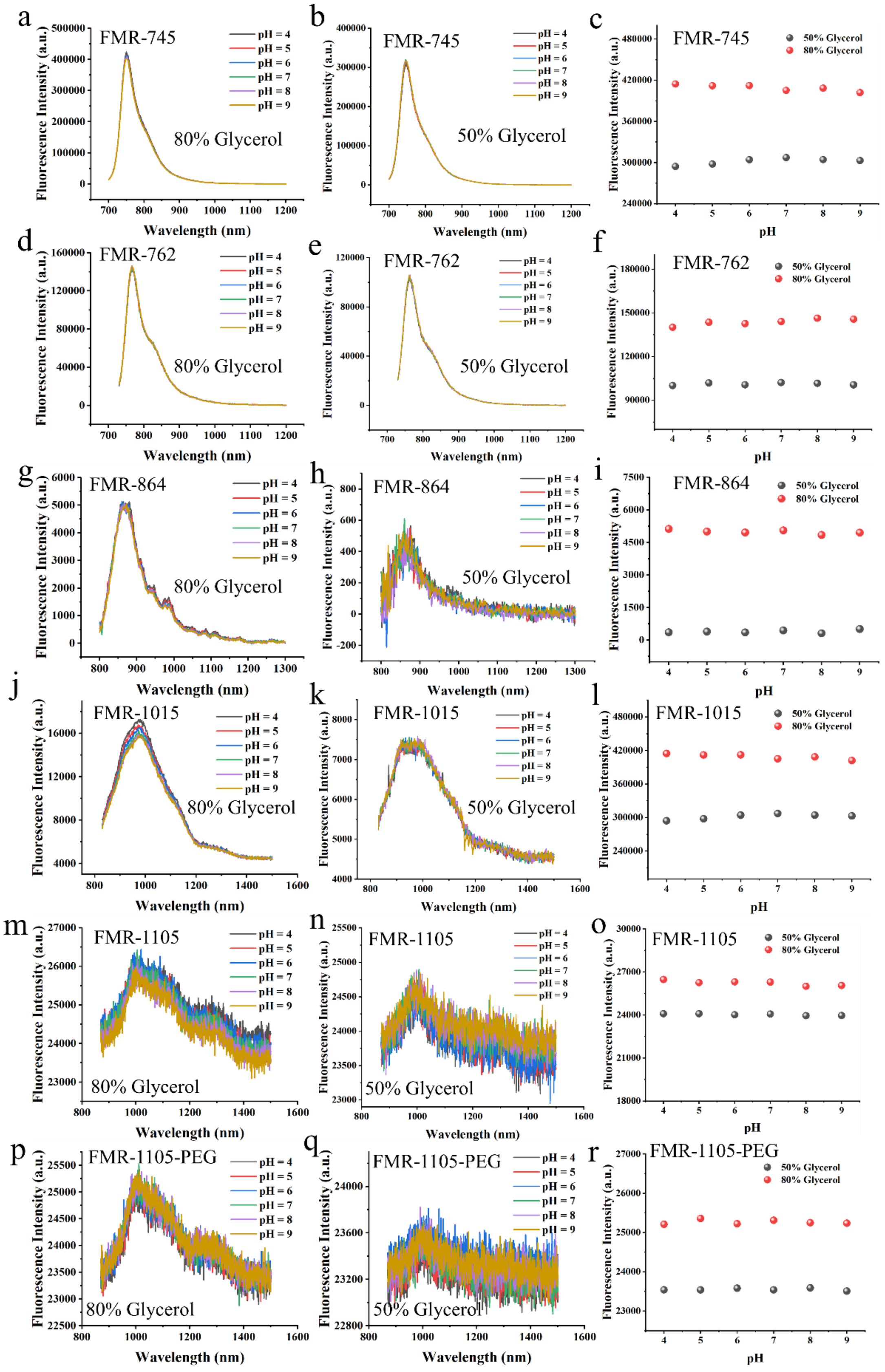

Figure S13. Fluorescence responses of **FMRs** in water/glycerol mixed solutions under different pH conditions. The glycerol volume fractions were adjusted to 50% and 80% to provide different viscosity environments, and the fluorescence emission spectra and quantitative fluorescence analyses of the dyes were obtained over a pH range of 4 - 9.

Penetration Depth Experiment.

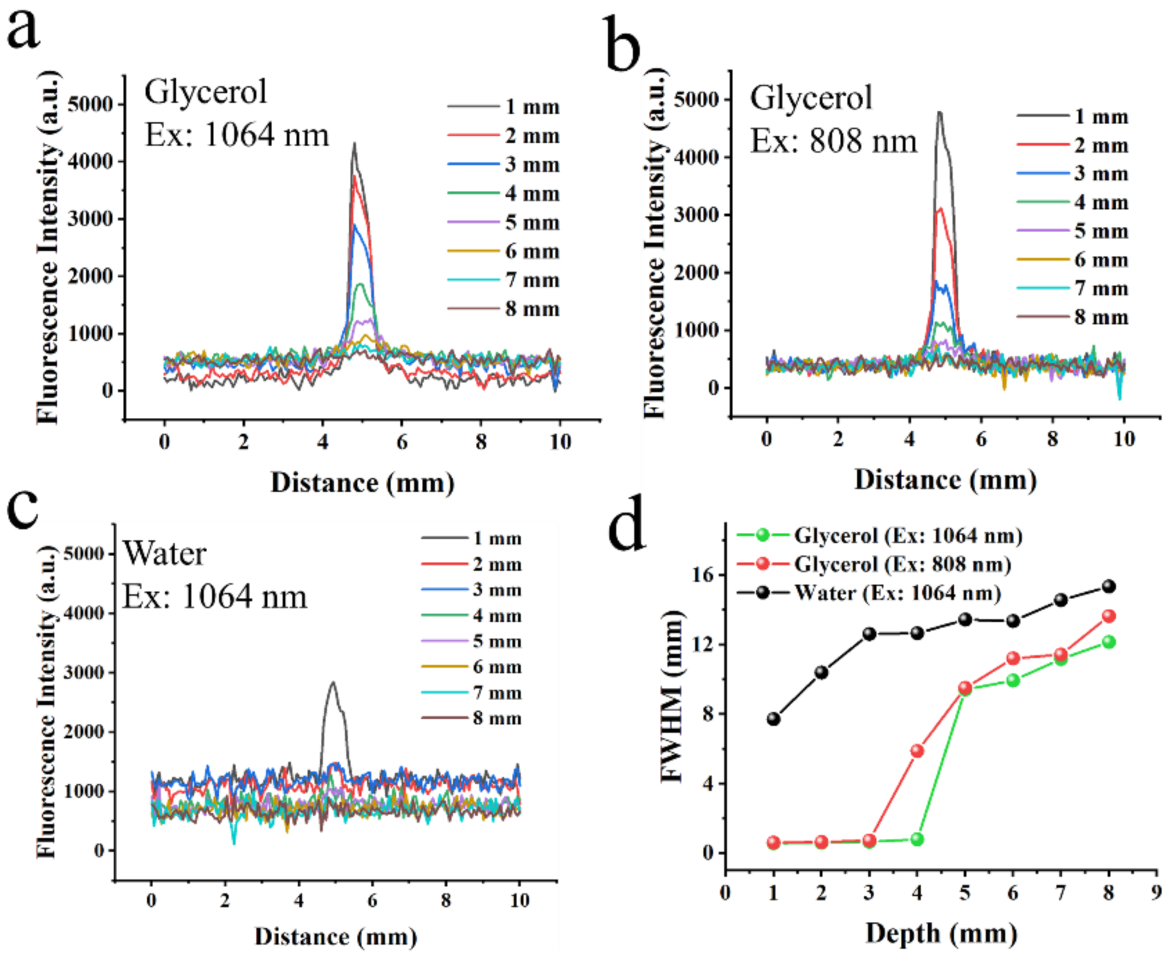

Figure S14. Cross-sectional fluorescence intensity profiles of **FMR-1105-PEG** in a capillary measured in glycerol under excitation at 1064 nm (a) and 808 nm (b), and in water under excitation at 1064 nm (c). (d) FWHMs obtained from Gaussian fits to the corresponding profiles.

NIR-II Imaging of the Non-Alcoholic Fatty Liver Mice Model.

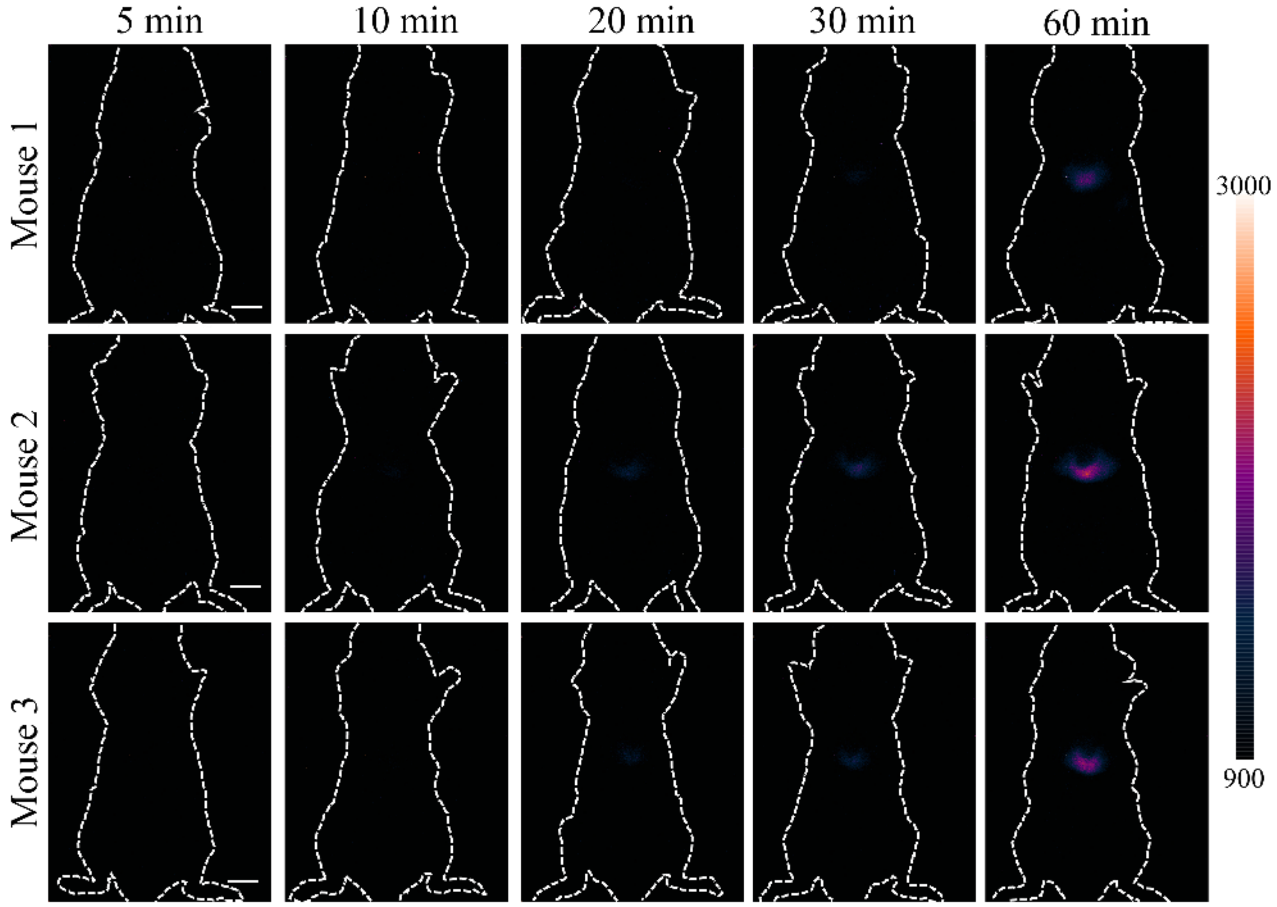

Figure S15. Time-dependent NIR-II imaging of control mice, fed a standard chow diet, following intravenous injection of **FMR-1105-PEG** (5 mg/kg, 200 μL) (n = 3). Scale bars: 1cm.

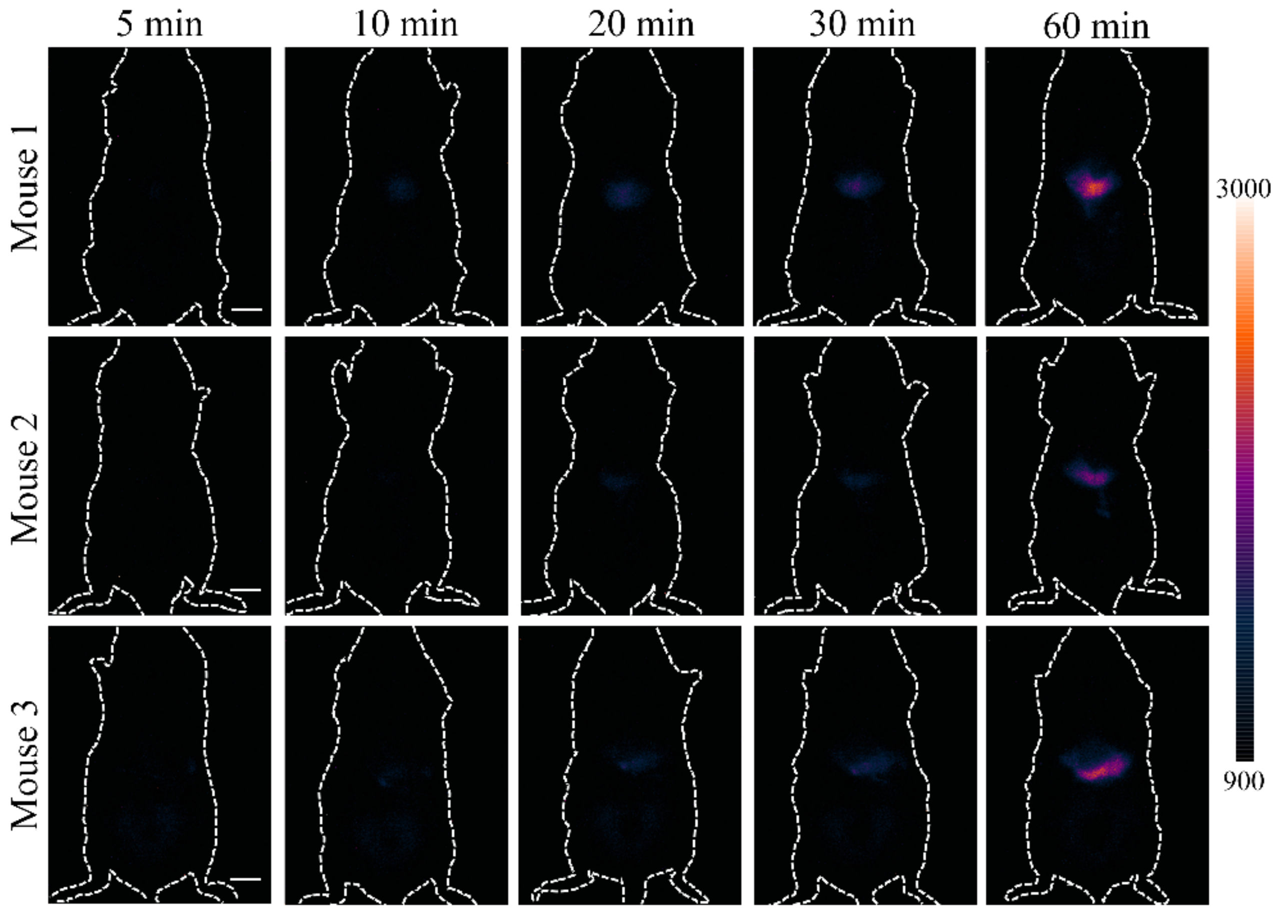

Figure S16. Time-dependent NIR-II imaging of NAFL mice, induced by a 60 kcal% high-fat diet plus daily dexamethasone injections for 3 consecutive days, following intravenous injection of **FMR-1105-PEG** (5 mg/kg, 200 μL) (n = 3). Scale bars: 1cm.

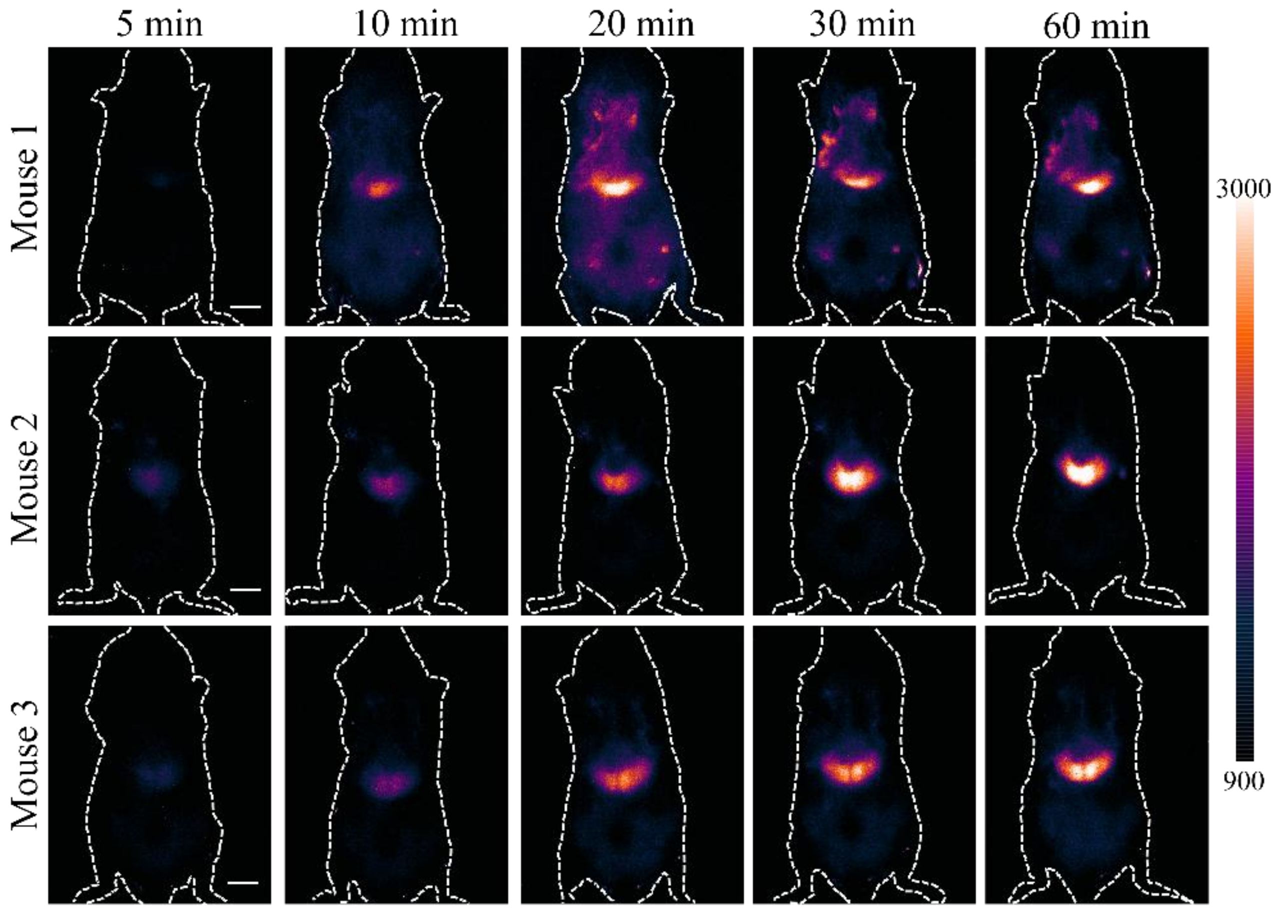

Figure S17. Time-dependent NIR-II imaging of NAFL mice, induced by a 60 kcal% high-fat diet plus daily dexamethasone injections for 9 consecutive days, following intravenous injection of **FMR-1105-PEG** (5 mg/kg, 200 μL) (n = 3). Scale bars: 1cm.

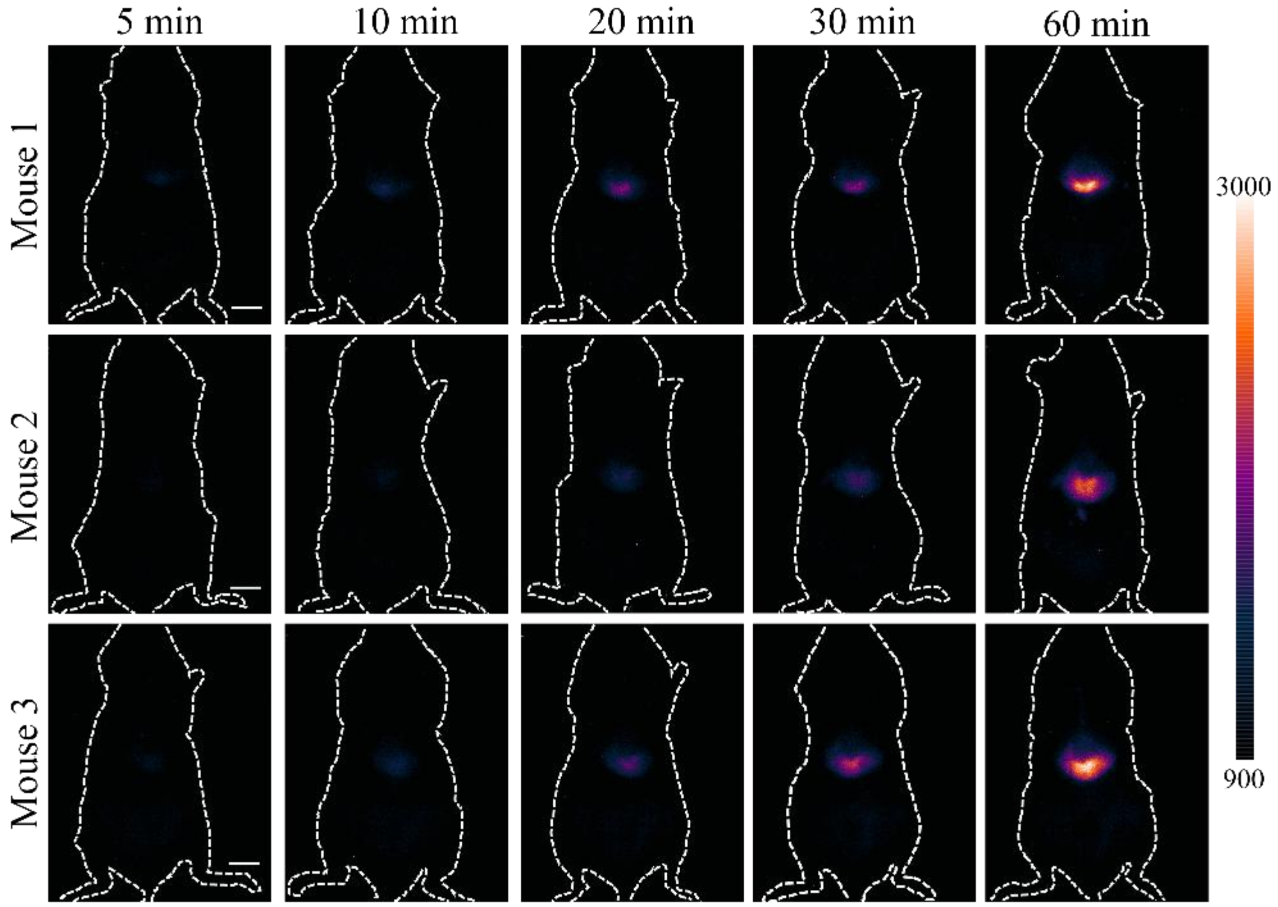

Figure S18. Time-dependent NIR-II imaging of NAFL+NAC mice, induced by a 60 kcal% high-fat diet plus daily dexamethasone injections for 9 consecutive days with NAC administered by oral gavage every other day, following intravenous injection of **FMR-1105-PEG** (5 mg/kg, 200 μL) (n = 3). Scale bars: 1cm.

NIR-II Imaging of the Acute Liver Injury Mice Model.

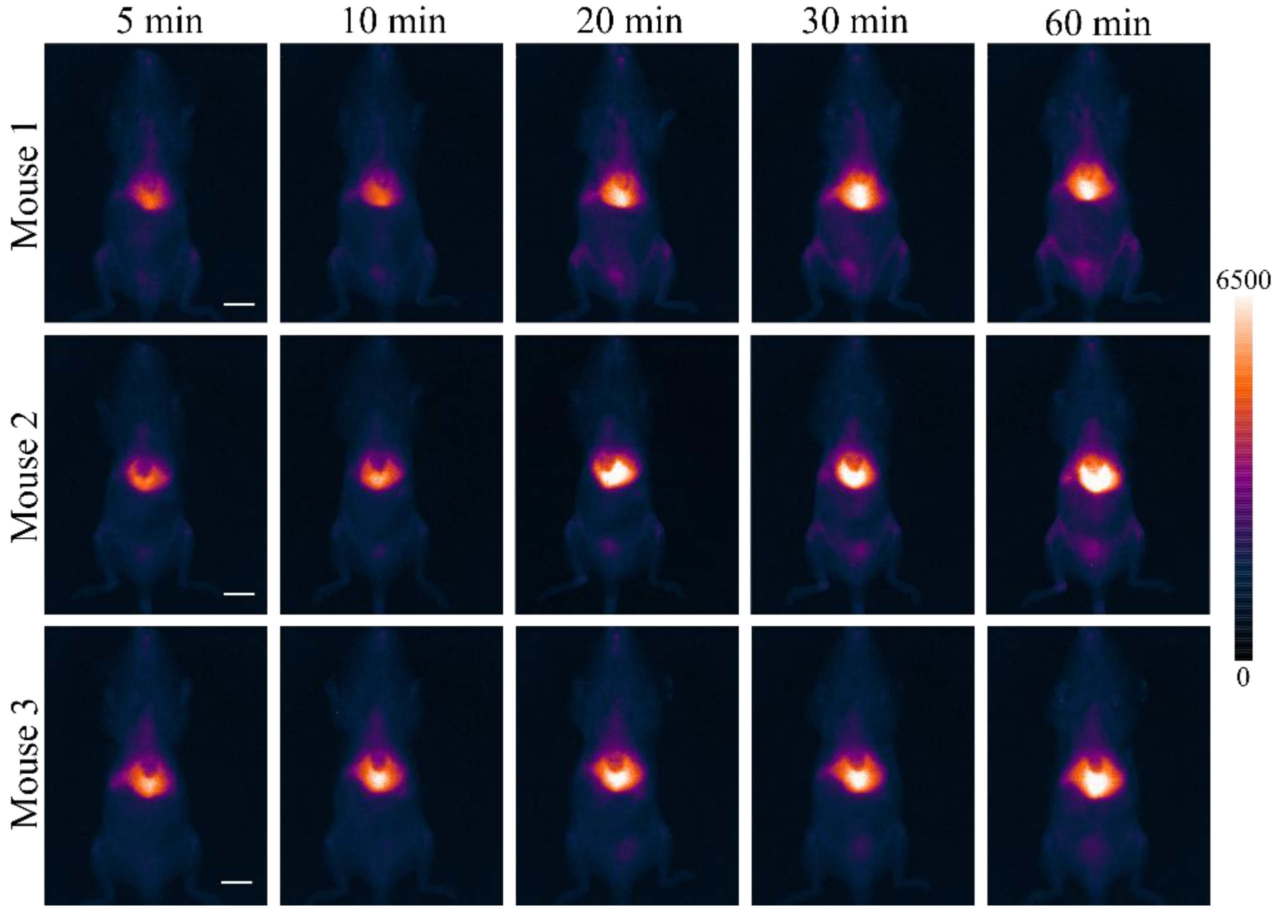

Figure S19. Time-dependent NIR-II imaging of acute liver injury mice (acetaminophen dose: 600 mg/kg) following intravenous injection of **FMR-1105-PEG** (5 mg/kg, 200 μL) (n = 3). Scale bars: 1cm.

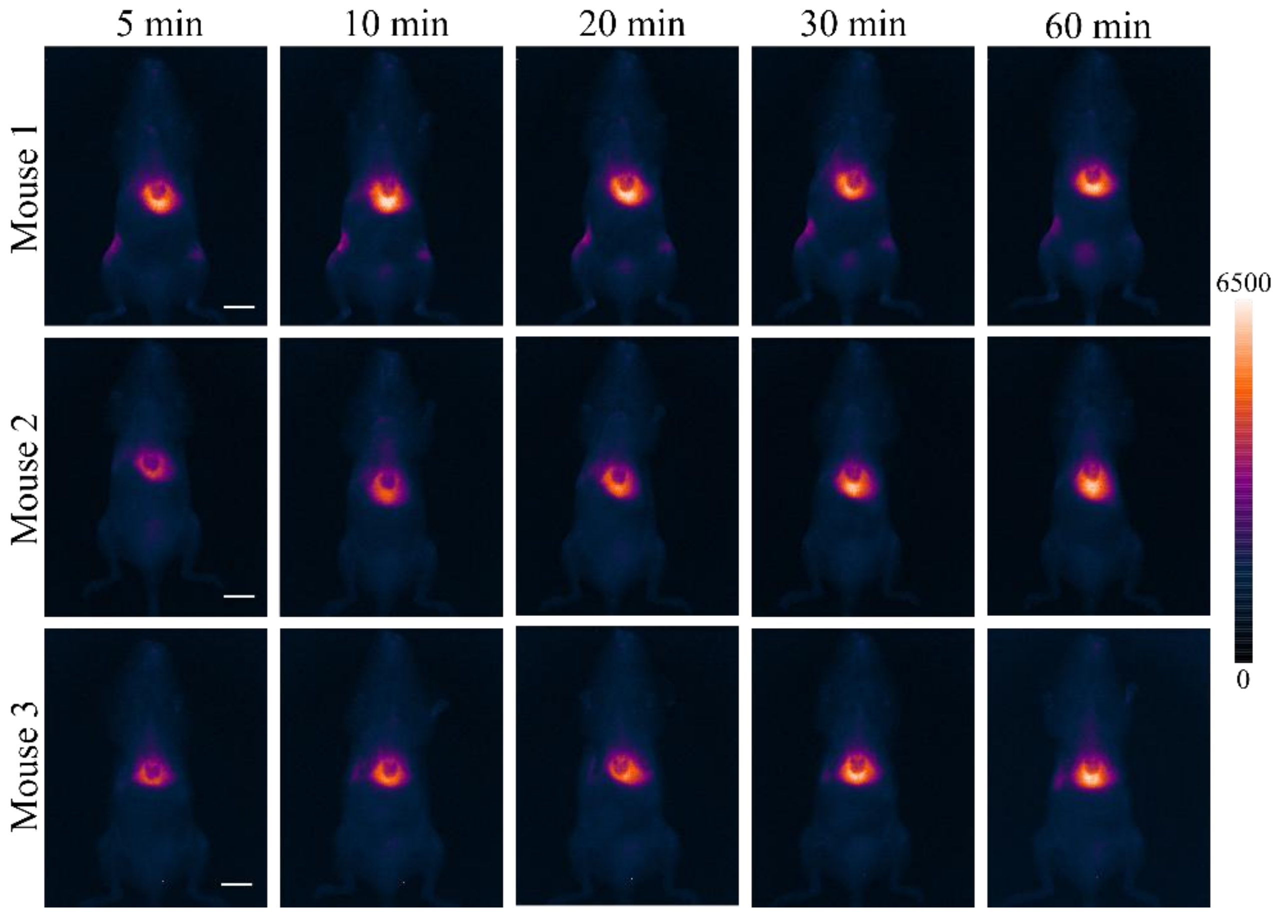

Figure S20. Time-dependent NIR-II imaging of acute liver injury mice (acetaminophen dose: 400 mg/kg) following intravenous injection of **FMR-1105-PEG** (5 mg/kg, 200 μL) (n = 3). Scale bars: 1cm.

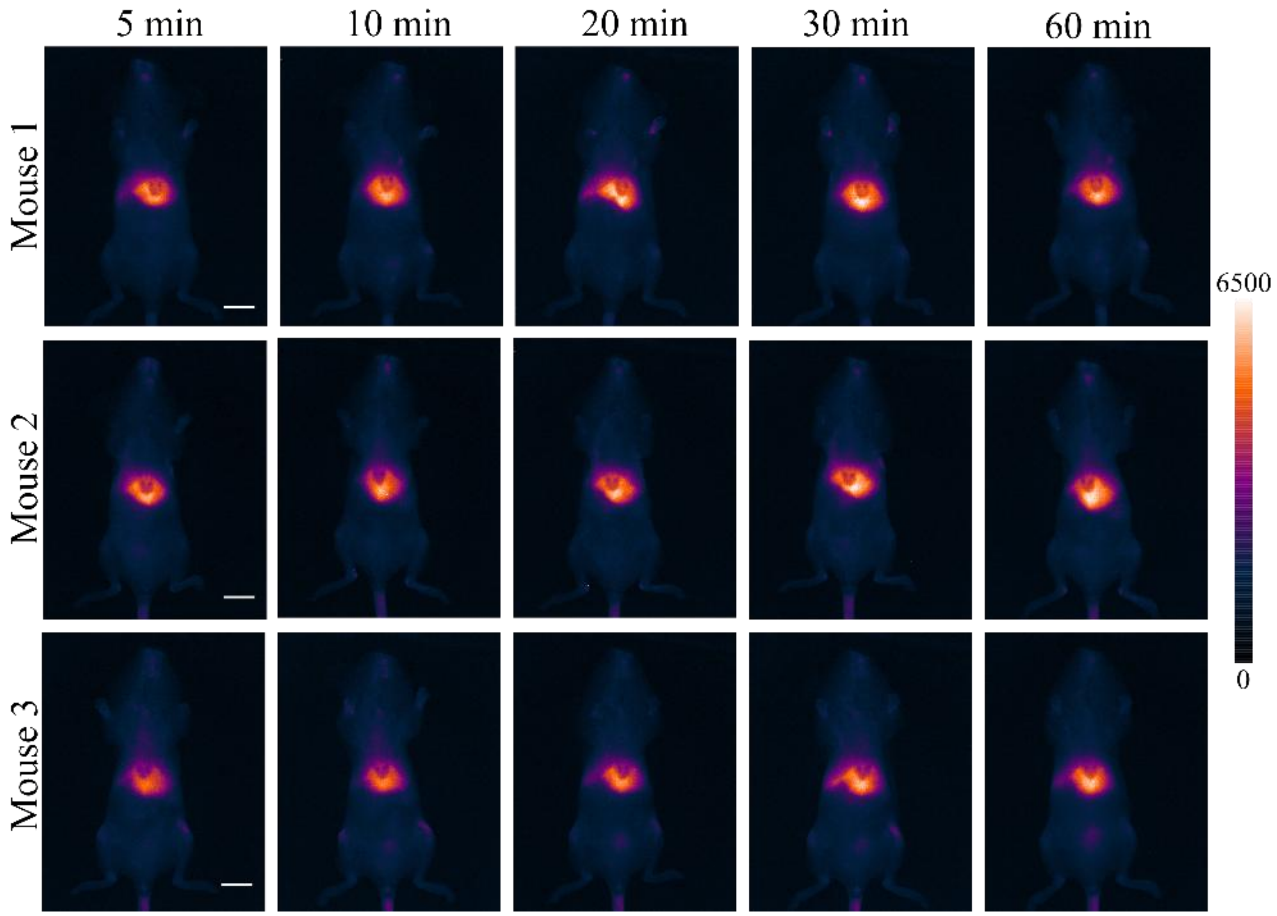

Figure S21. Time-dependent NIR-II imaging of acute liver injury mice (acetaminophen dose: 200 mg/kg) following intravenous injection of **FMR-1105-PEG** (5 mg/kg, 200 μL) (n = 3). Scale bars: 1cm.

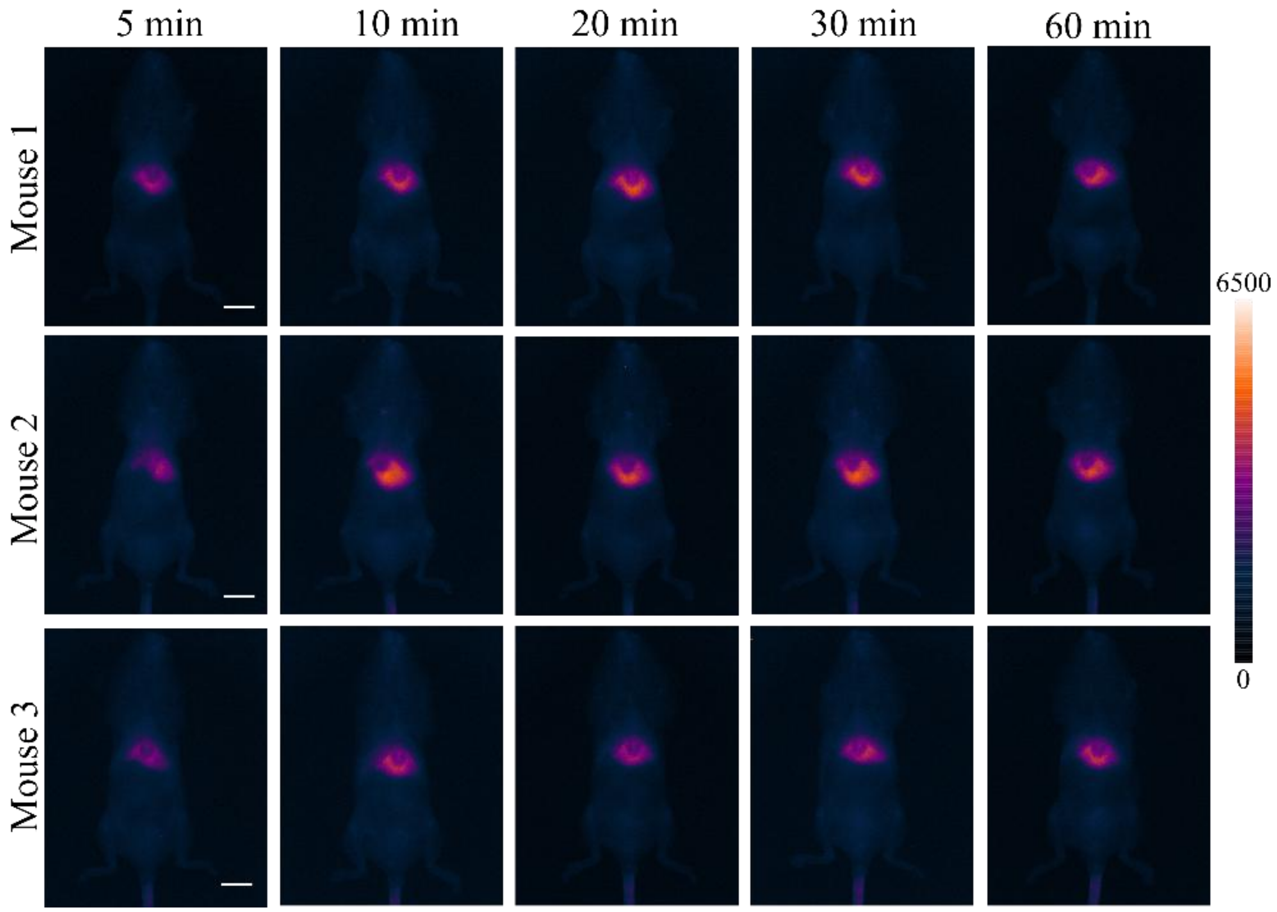

Figure S22. Time-dependent NIR-II imaging of control mice (no intraperitoneal acetaminophen) following intravenous injection of **FMR-1105-PEG** (5 mg/kg, 200 μL) (n = 3). Scale bars: 1cm.

NIR-II Imaging of 4T1 Tumor-bearing Mice.

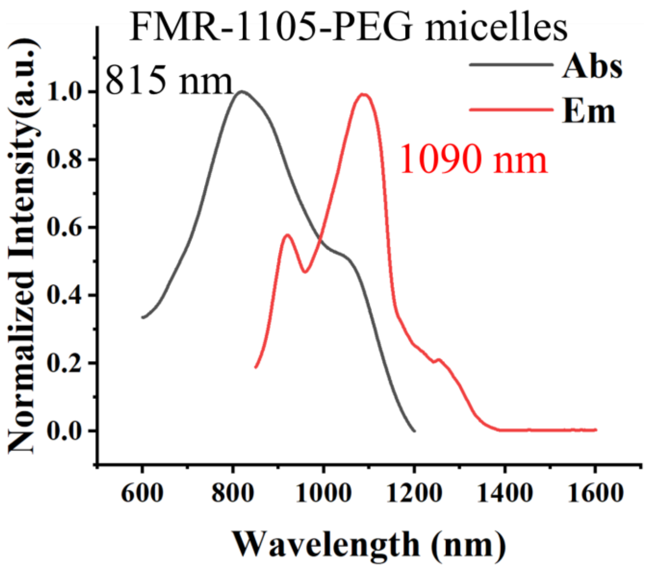

Figure S23. Absorption and emission spectra of **FMR-1105-PEG** micelles were recorded in PBS at pH 7.4, with a concentration of 10 μM

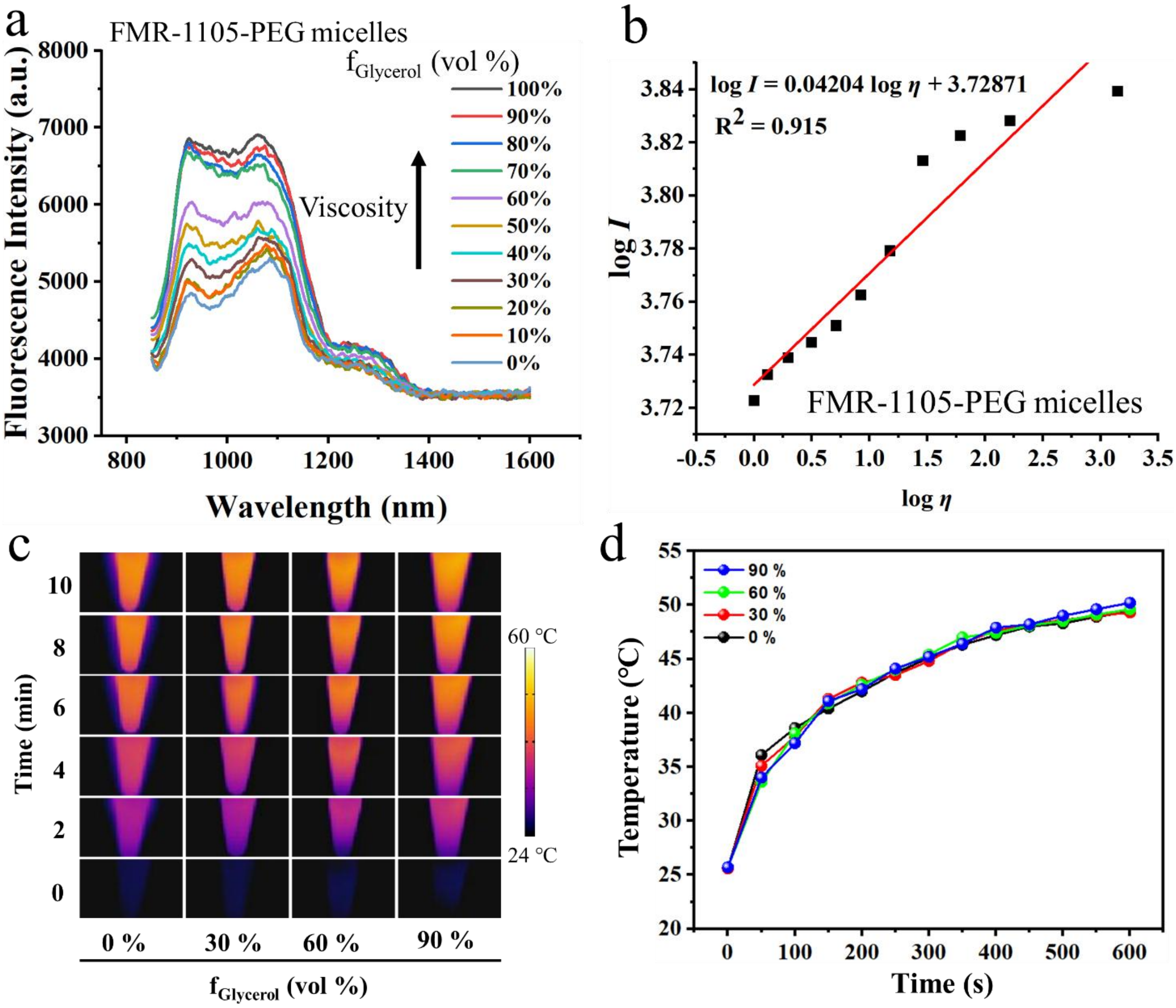

Figure S24. The viscosity of the system was adjusted by mixing water and glycerol at different volume ratios, and the effects of viscosity on the fluorescence and photothermal performance of **FMR-1105-PEG** micelles were recorded. (a) Fluorescence emission spectra of **FMR-1105-PEG** micelles recorded in glycerol/water mixtures with glycerol volume fractions of 0 – 100 vol %. (b) Viscosity calibration curve obtained from a log–log plot of fluorescence intensity (I) versus viscosity (η). (c) Infrared thermal images of **FMR-1105-PEG** micelle solutions dispersed in glycerol/water mixtures with glycerol volume fractions of 0%, 30%, 60%, and 90% under 1064 nm laser irradiation (1 W/cm²) for 10 min. (d) Corresponding temperature-time heating profiles.

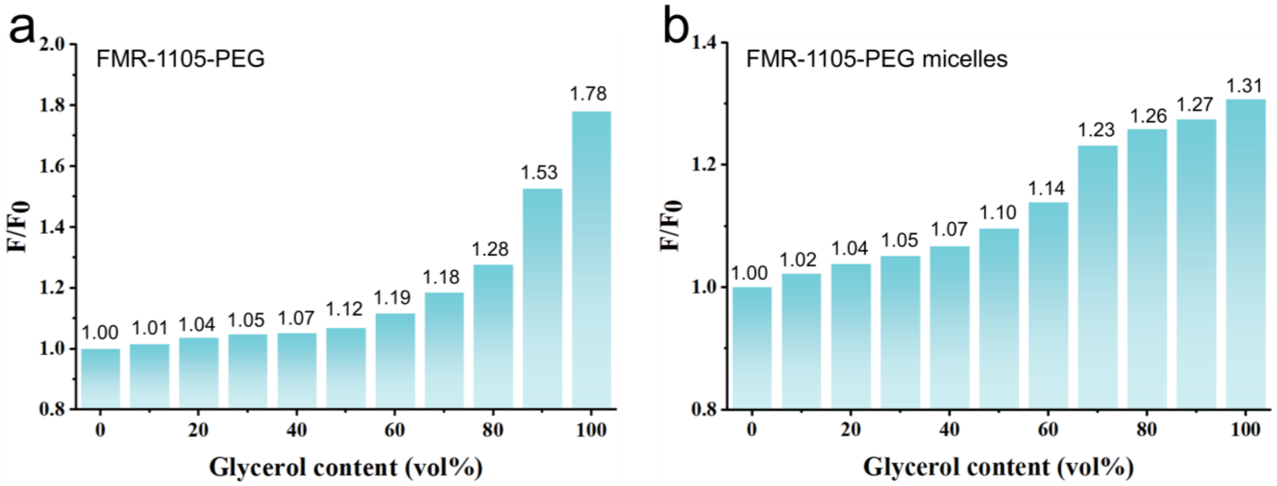

Figure S25. Relative fluorescence intensity ratio (F/F_0) of **FMR-1105-PEG** (a) and **FMR-1105-PEG** micelles (b) as a function of glycerol content (0-100 vol%). F_0 and F represent the fluorescence intensities in pure water (0 vol% glycerol) and the respective water/glycerol mixtures, respectively. The specific F/F_0 values are explicitly labeled above each column to quantify the fold-increase in intensity.

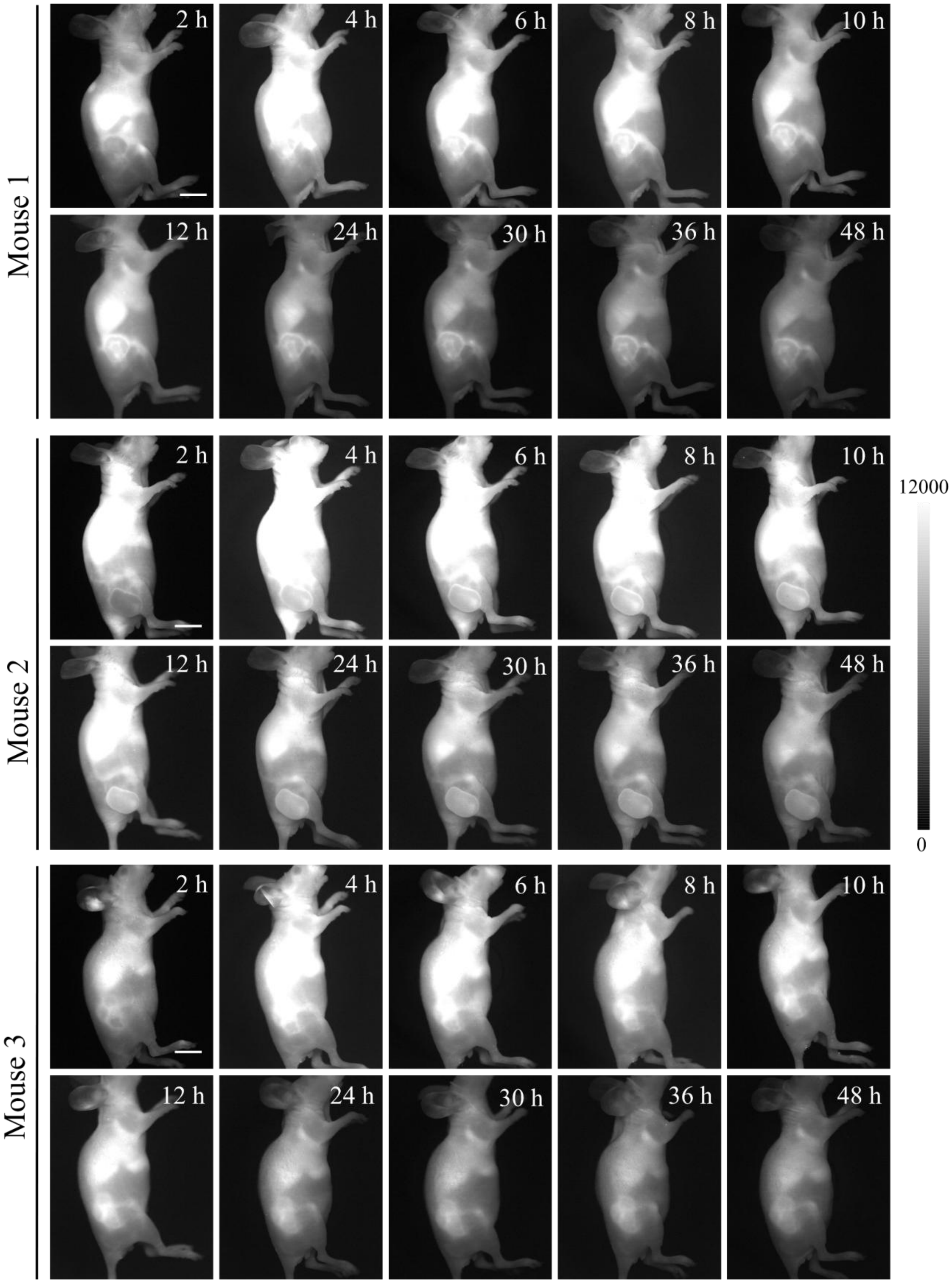

Figure S26. Time-dependent NIR-II imaging of 4T1 tumor-bearing mice after injection of **FMR-1105-PEG** micelles (25 mg/kg, 100 μL, calculated from the dye concentration) at multiple time points. Scale bar: 1 cm.

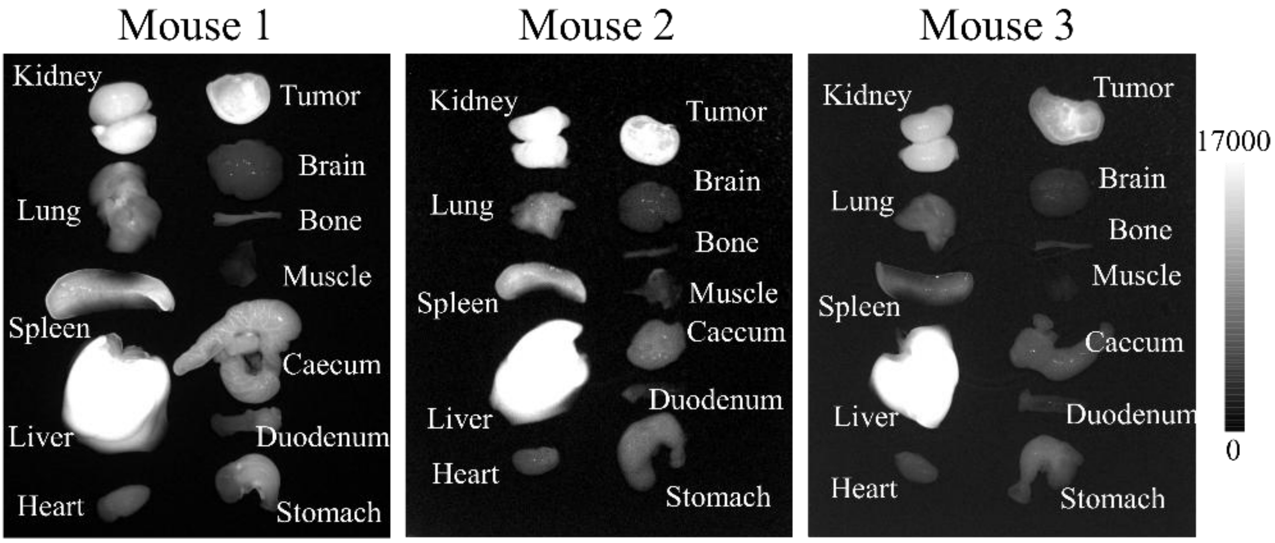

Figure S27. *Ex vivo* biodistribution of 4T1 tumor-bearing mice after injection of **FMR-1105-PEG** micelles at 48 h post-injection.

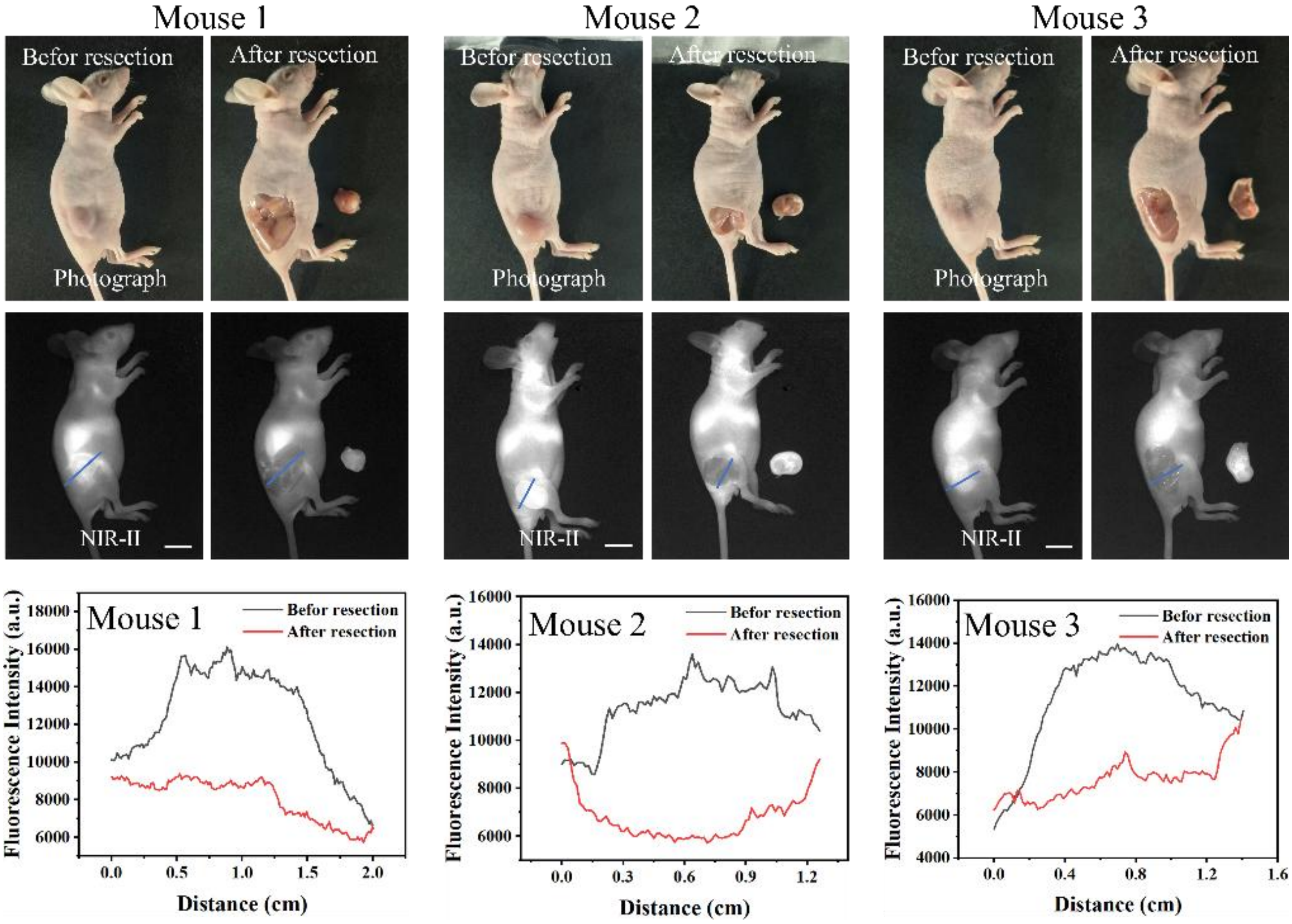

Figure S28. Pre- and post-resection imaging of the 4T1 tumor model using NIR-II fluorescence, showing clear tumor delineation before resection and fluorescence reduction after tumor removal. Scale bar: 1 cm.

Photothermal Performance and Photothermal Conversion Efficiency Calculations.

To evaluate the photothermal performance of **FMR-1105-PEG** micelles, 1 mL of **FMR-1105-PEG** micelle solution (300 μg/mL) was irradiated with a 1064 nm laser (1.0 W/cm^2), and the temperature was monitored until a steady-state maximum was reached. Temperature profiles were recorded using an infrared thermal camera. Subsequently, temperature changes of **FMR-1105-PEG** micelle solutions in PBS at different concentrations (0, 100, 200, 300, 500, and 1000 μg/mL) were measured under 1064 nm laser irradiation (1.0 W/cm^2, 10 min). In addition, temperature changes of **FMR-1105-PEG** micelle solutions (300, 500, and 1000 μg/mL) were recorded under 1064 nm laser irradiation at different power densities (0.50, 0.75, 1.00, and 1.50 W/cm^2; 10 min). To assess photothermal stability, 1 mL of **FMR-1105-PEG** micelles in PBS (500 μg/mL) was subjected to 1064 nm laser irradiation (1.0 W/cm^2) for five successive heating–cooling cycles. Furthermore, to mimic tissue attenuation, the temperature rise after 10 min irradiation with 1064 nm and 808 nm lasers was measured with the sample tubes covered by 6 mm-thick chicken breast tissue, and the corresponding thermal images were acquired. The photothermal conversion efficiency (PCE) was calculated using Roper's method according to the following equation:

$$\eta=\frac{\mathrm{h}s(T_{max}-T_{surr})-Q_{dis}}{I(1-10^{-A1064})}$$

where "η" is the photothermal conversion efficiency; "h" is the heat transfer coefficient; "s" is the surface area of the container; "T_{max} " is the maximum steady-state temperature under laser irradiation; "T_{surr}" is the ambient temperature; "Q_{dis}" represents the heat dissipation arising from laser absorption by the solvent and container; "I" is the incident laser power; and "A_{1064}" is the absorbance of the sample at 1064 nm.

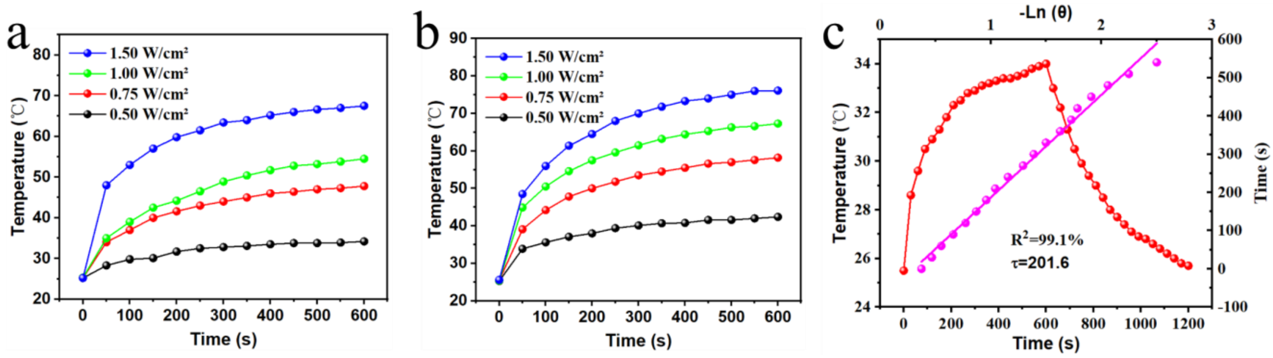

Figure S29. Photothermal heating profiles of **FMR-1105-PEG** micelle solutions under 1064 nm laser irradiation and determination of the system time constant. (a) Temperature–time curves of 1 mL **FMR-1105-PEG** micelle aqueous solution (500 μg/mL) irradiated at different power densities (0.50, 0.75, 1.00, and 1.50 W/cm^2) for 10 min. (b) Temperature–time curves of 1 mL **FMR-1105-PEG** micelle aqueous solution (1000 μg/mL) under the same conditions. (c) Heating/Cooling curve of 1 mL PBS and the corresponding linear fit of time versus -ln(θ) used to obtain the system time constant (τ).

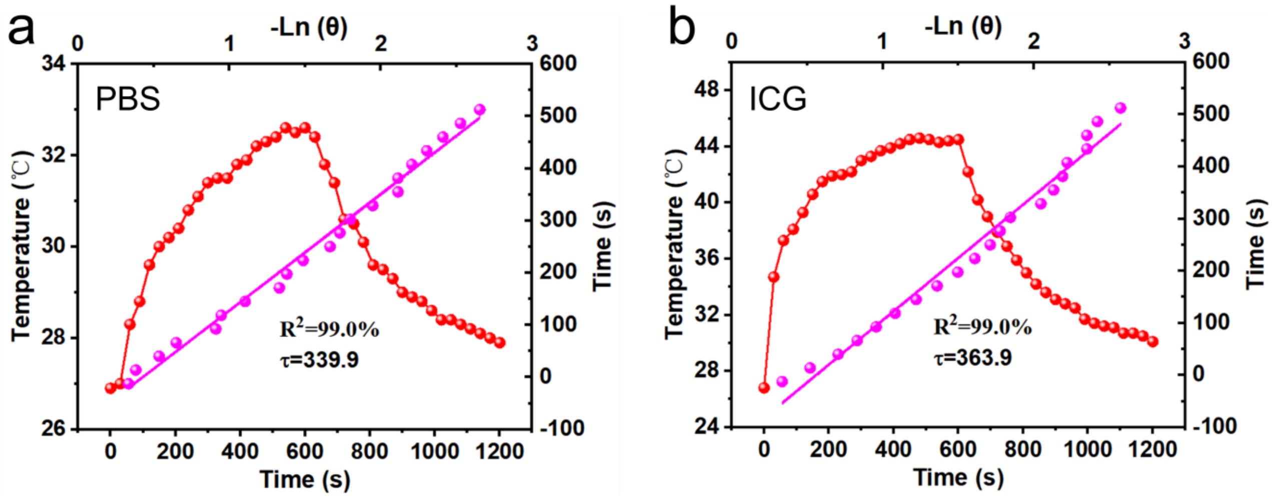

Figure S30. Photothermal heating and cooling profiles of PBS and **ICG** (100 μM) under 808 nm laser irradiation. The temperature change over time during and after laser irradiation for PBS (a) and **ICG** (b), respectively. The corresponding cooling time versus -ln(θ) linear fitting used to calculate the photothermal time constant (τ).

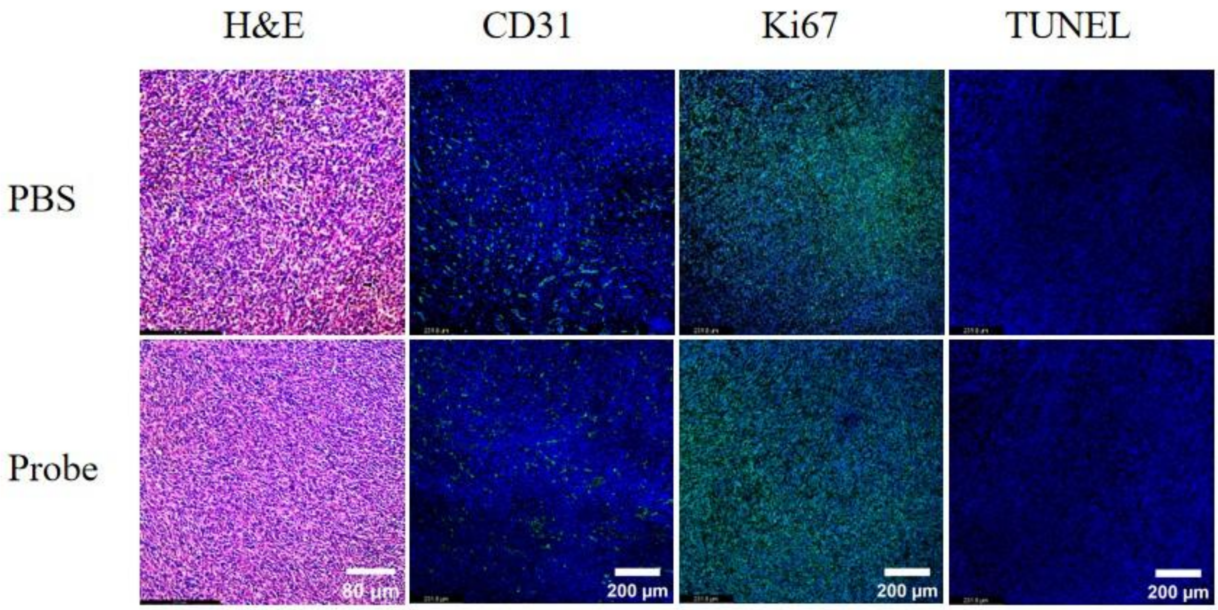

Figure S31. Histological and immunostaining analyses of tumor tissues after tail-vein injection of PBS or **FMR-1105-PEG** micelles. From left to right: H&E, CD31, Ki67, and TUNEL staining. Scale bars: 80 μm for H&E and 200 μm for the others.

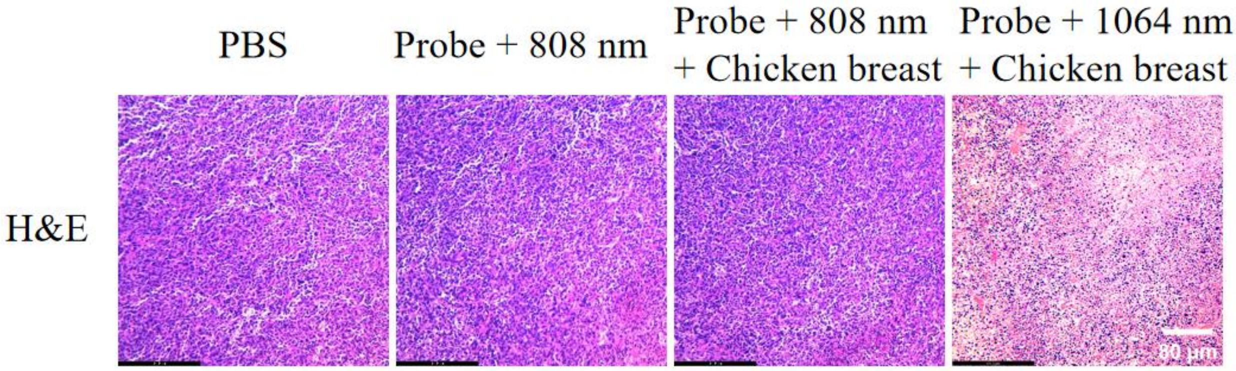

Figure S32. H&E staining images of tumor tissues after different treatments. Scale bar: 80 μm.

Biological Safety Evaluation.

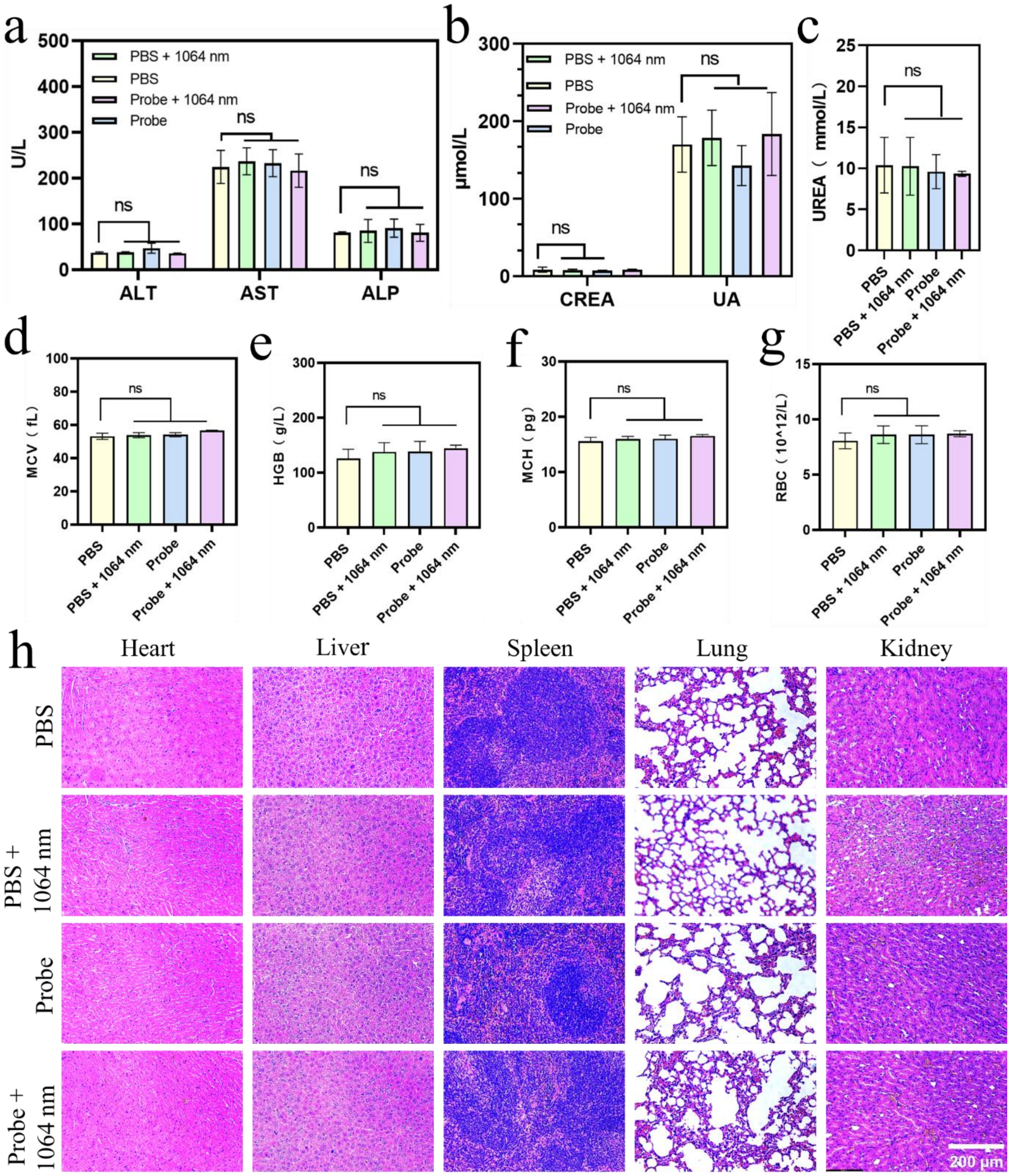

Figure S33. *In vivo* biosafety evaluation of Probe-mediated photothermal treatment. (a) Serum biochemistry markers for liver function (ALT, AST, ALP). (b,c) Renal function–related indices (CREA, UA, UREA). (d-g) Hematological parameters (WBC, HGB, MCH, RBC). (h) H&E staining of major organs (heart, liver, spleen, lung, and kidney) collected from mice in different groups (PBS, PBS + 1064 nm, Probe, and Probe + 1064 nm). ns, not significant. Scale bar: 200 μm.

NMR and MS Spectra.

^{1}H NMR spectrum of **Compound 2**.

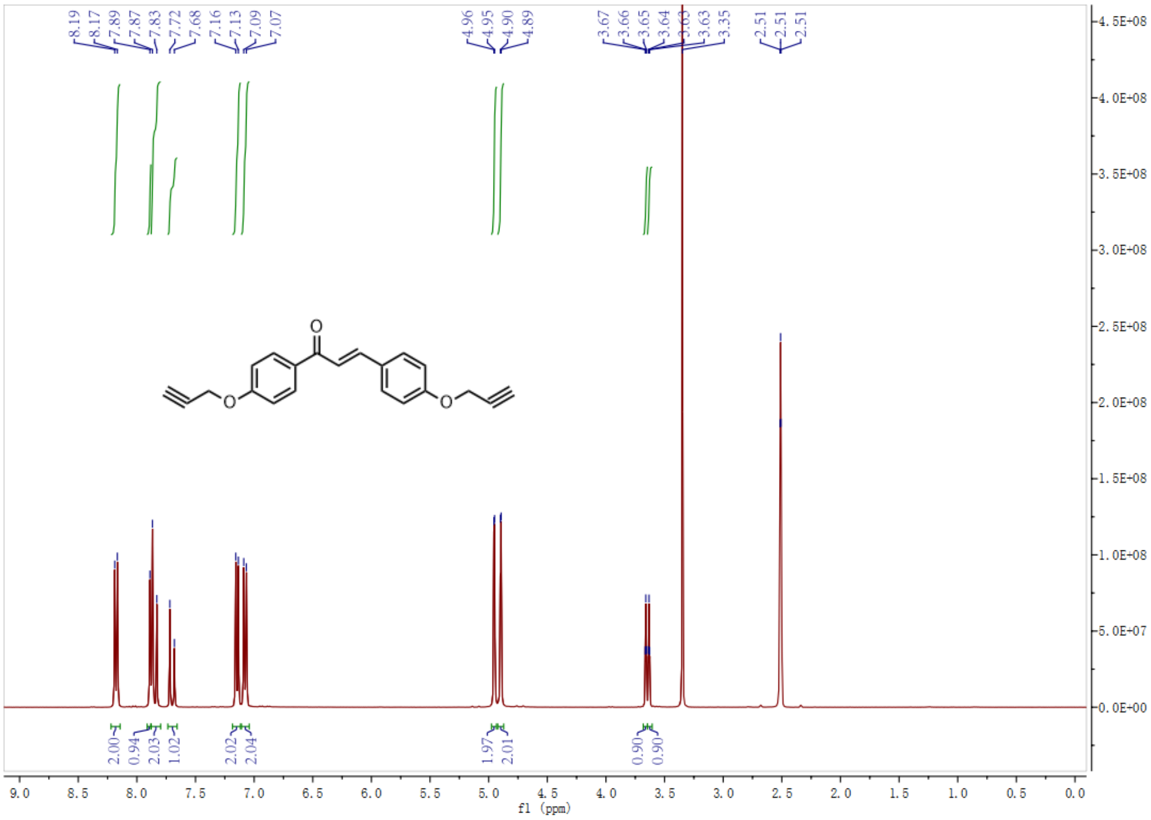

HRMS spectrum of **Compound 2**.

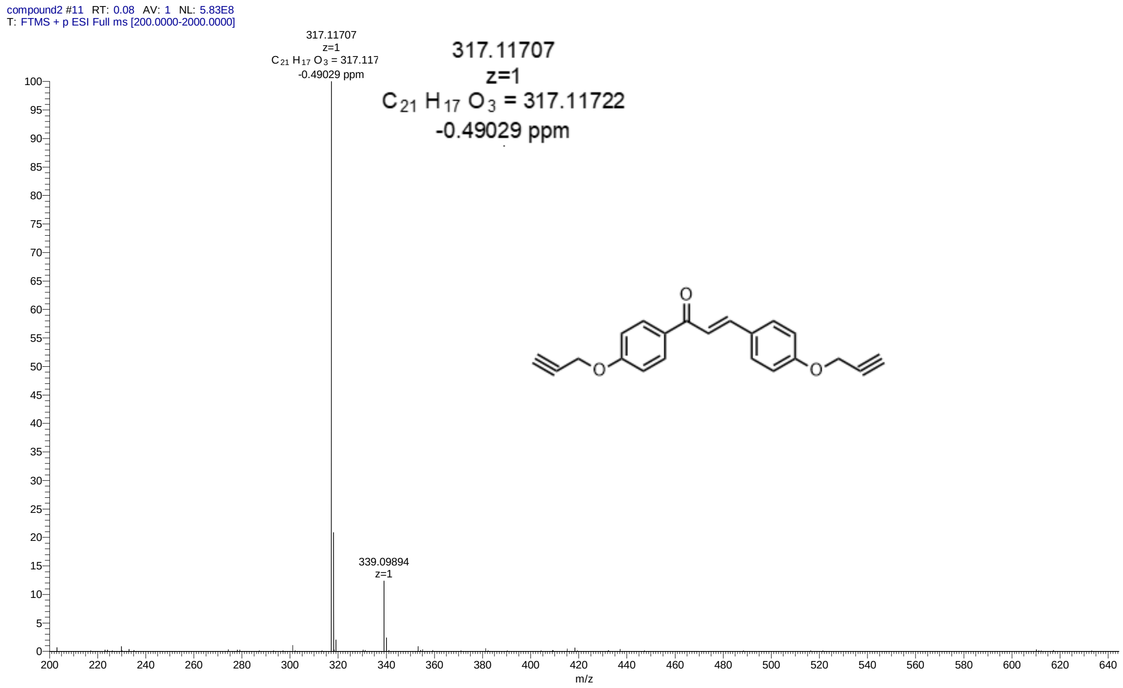

^{1}H NMR spectrum of **Compound 3**.

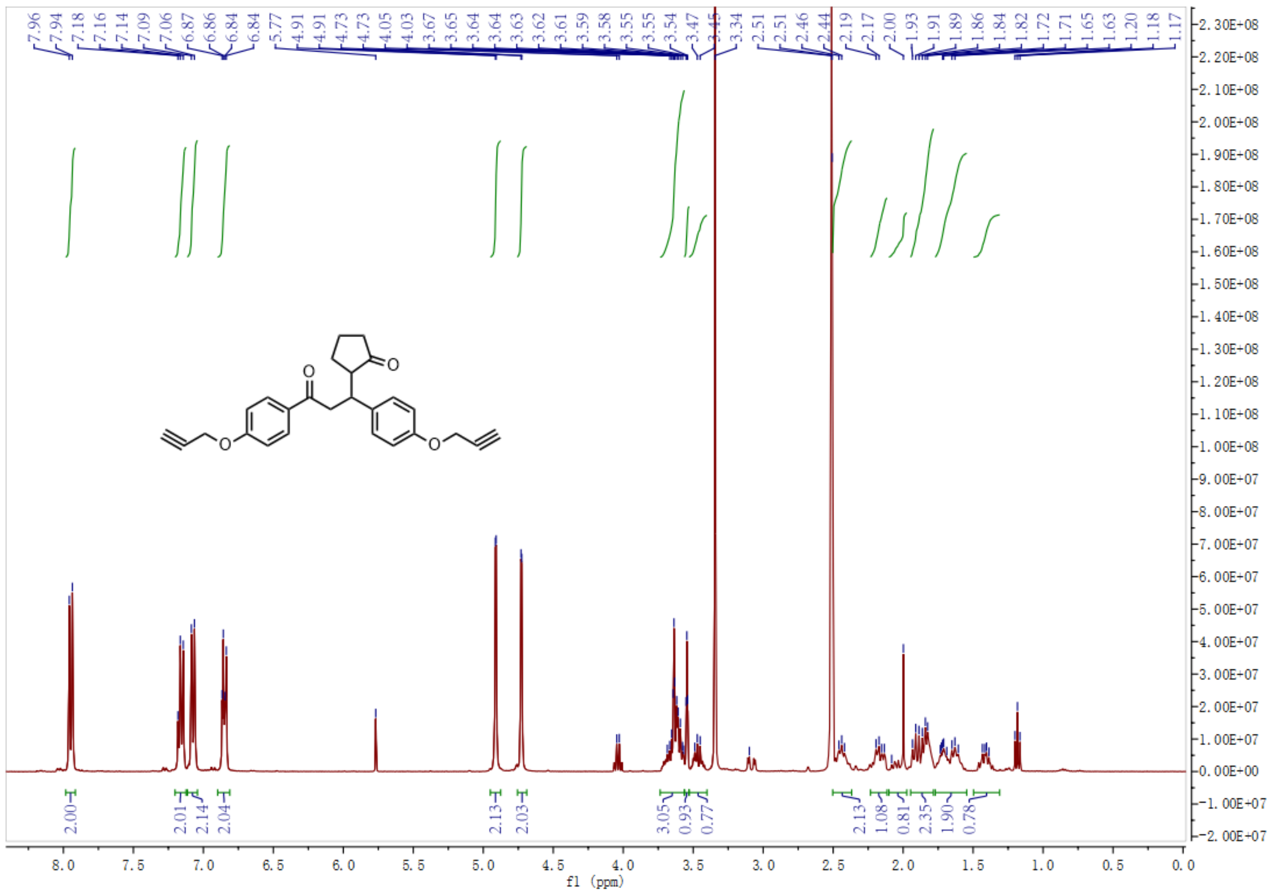

HRMS spectrum of **Compound 3**.

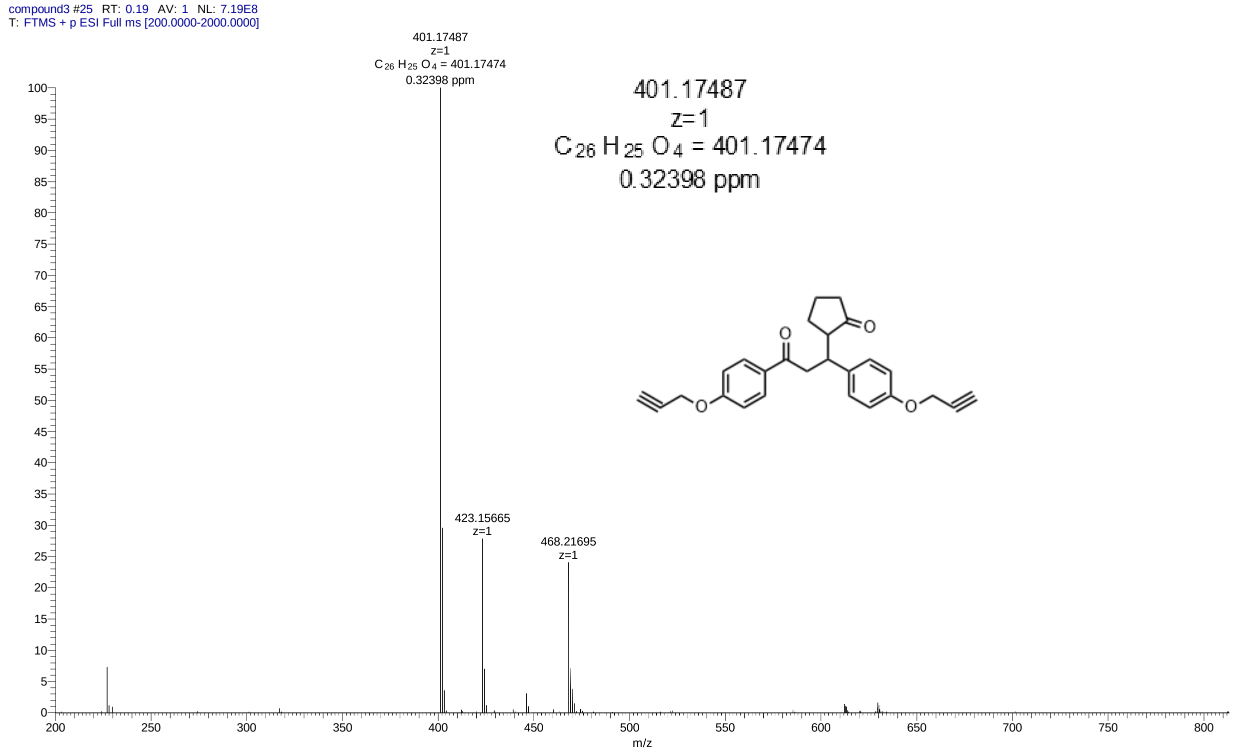

^{1}H NMR spectrum of **FMR-745**.

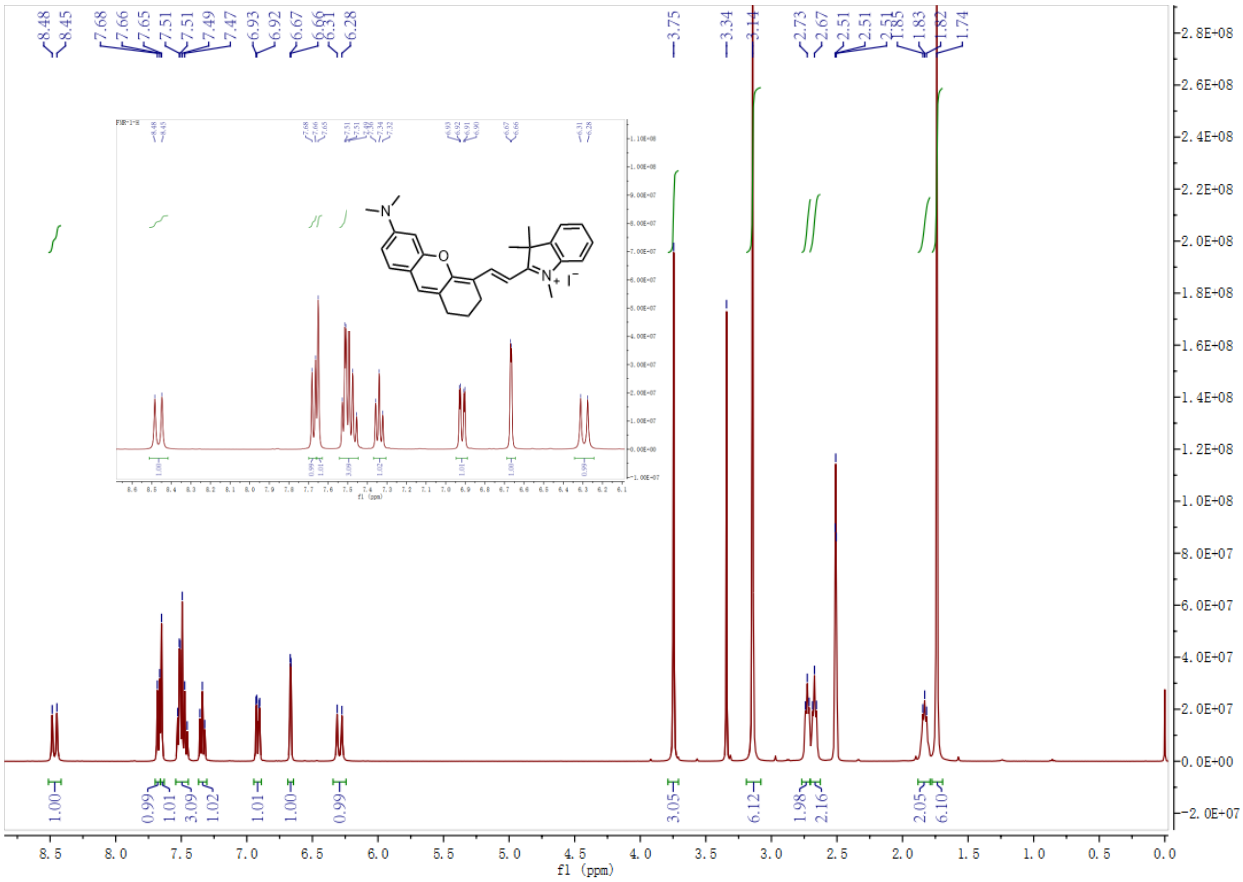

^{13}C NMR spectrum of **FMR-745**.

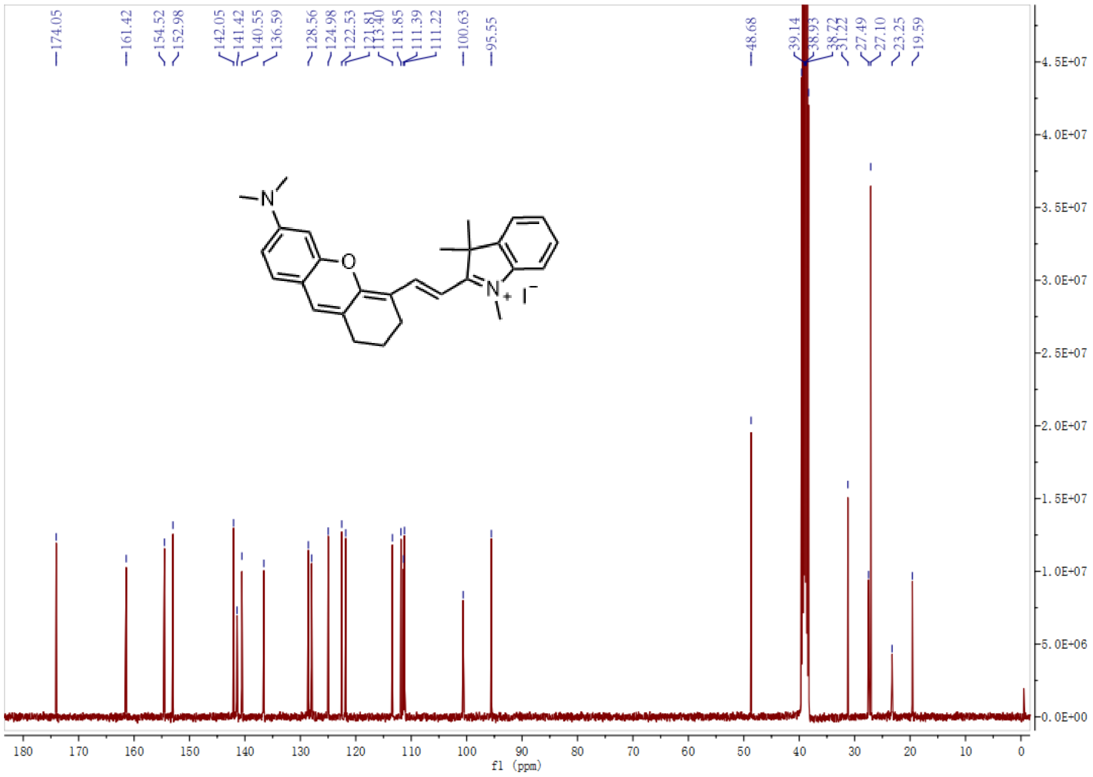

HRMS spectrum of **FMR-745**.

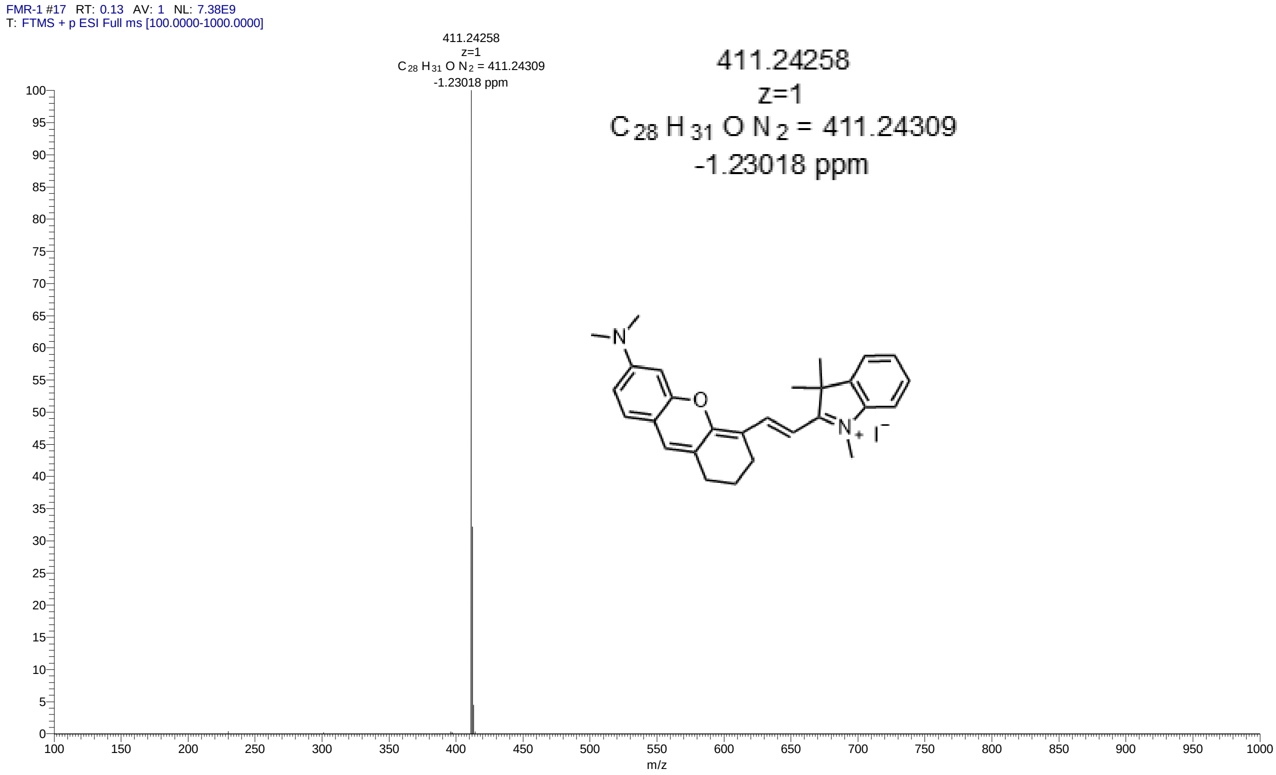

^{1}H NMR spectrum of **FMR-762**.

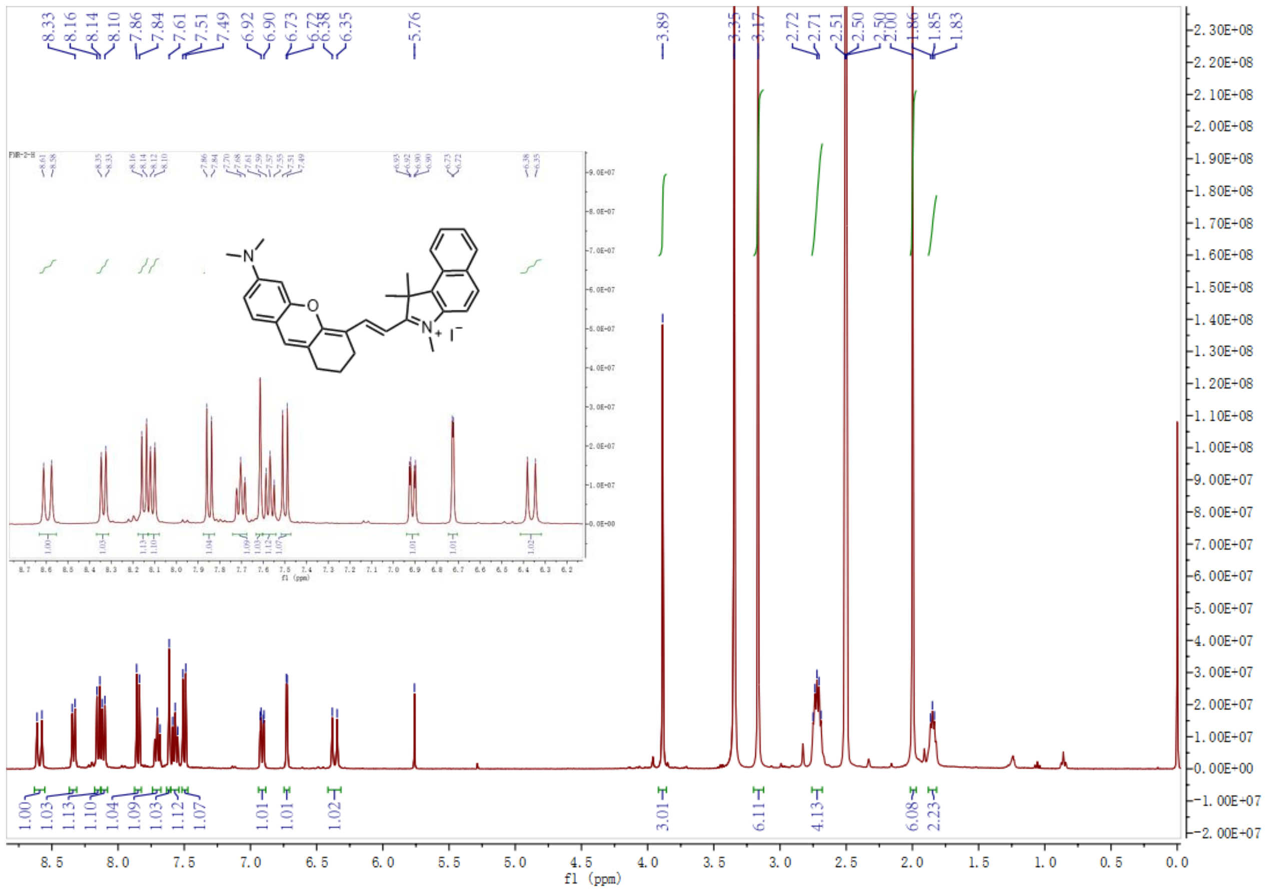

^{13}C NMR spectrum of **FMR-762**.

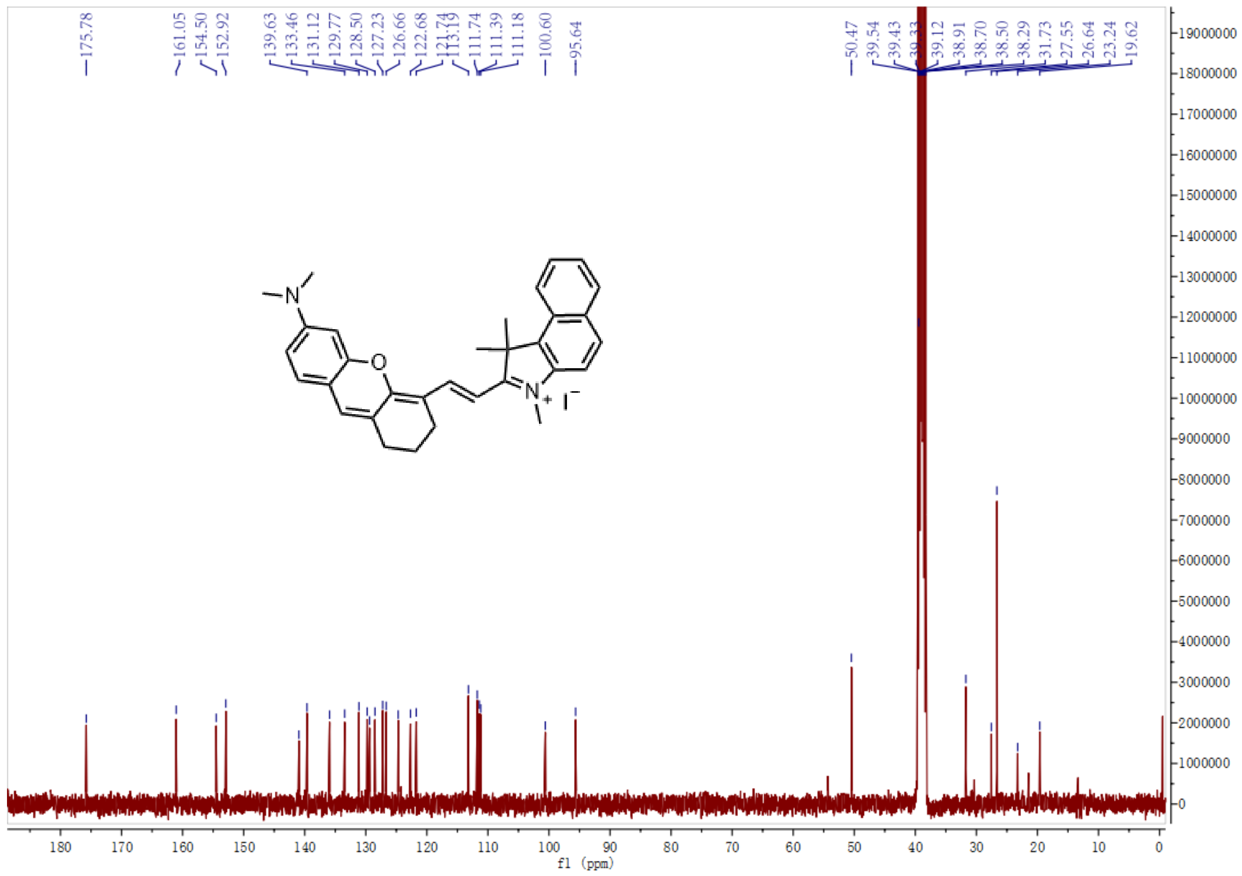

HRMS spectrum of **FMR-762**.

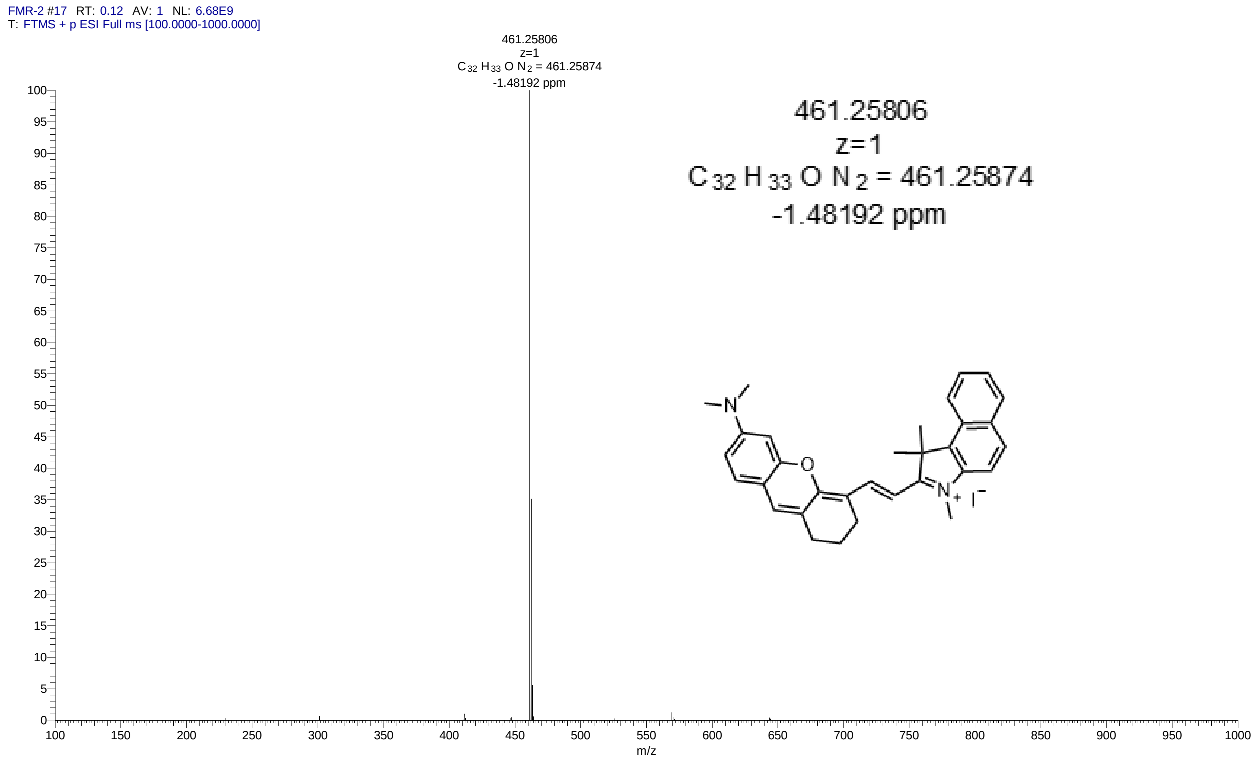

^{1}H NMR spectrum of **FMR-864**.

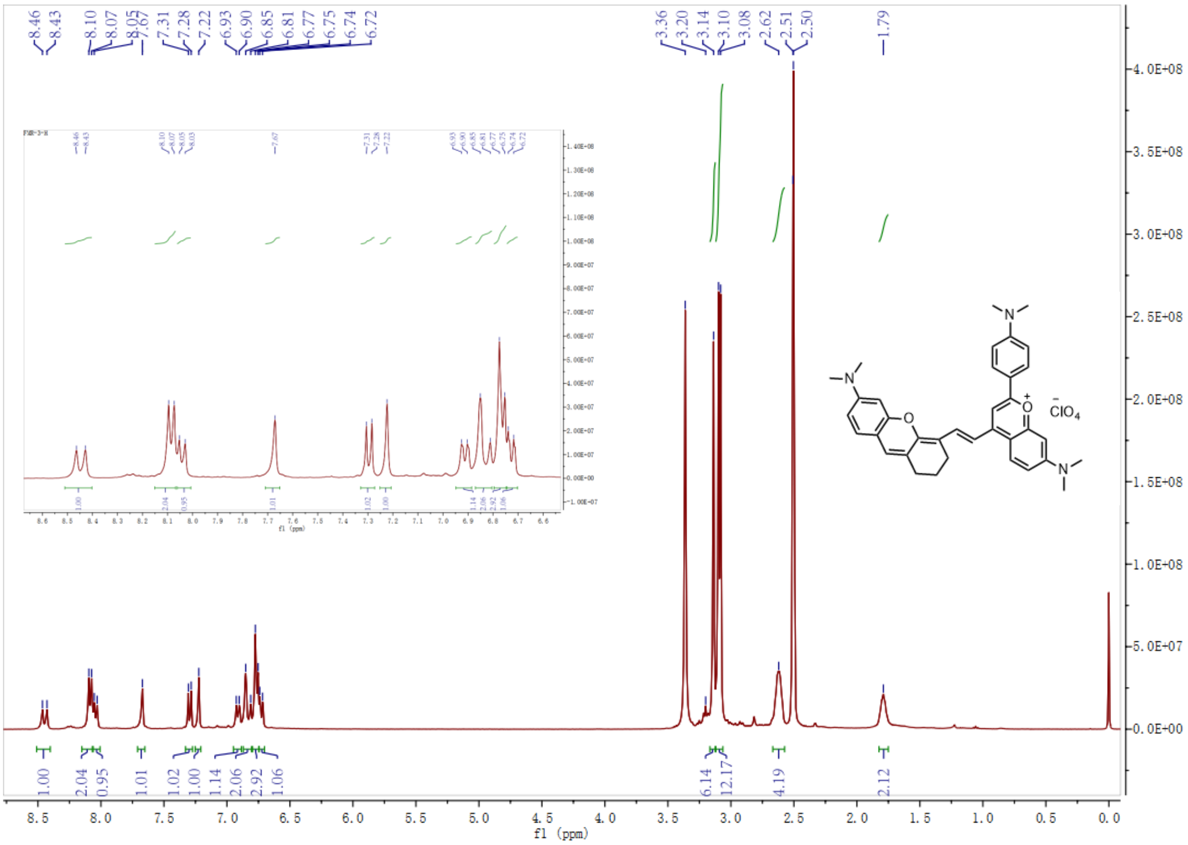

^{13}C NMR spectrum of **FMR-864**.

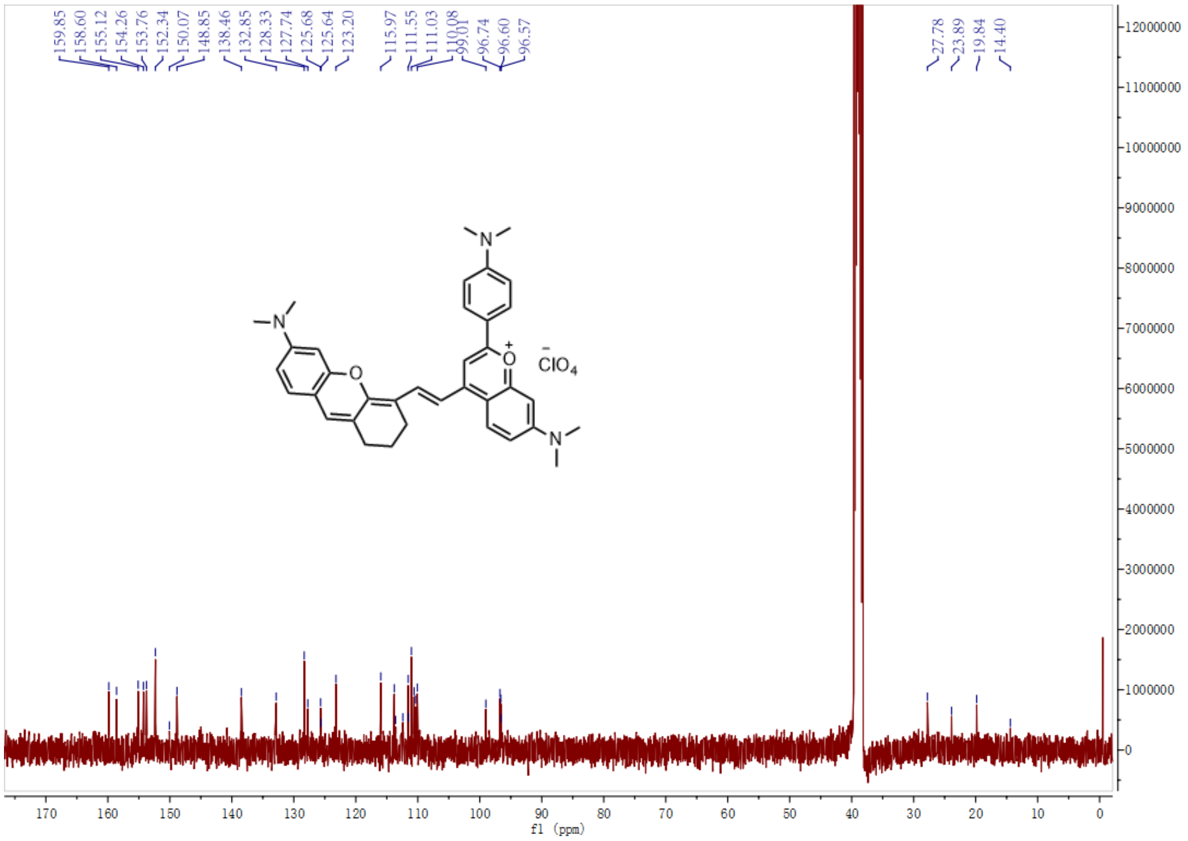

HRMS spectrum of **FMR-864**.

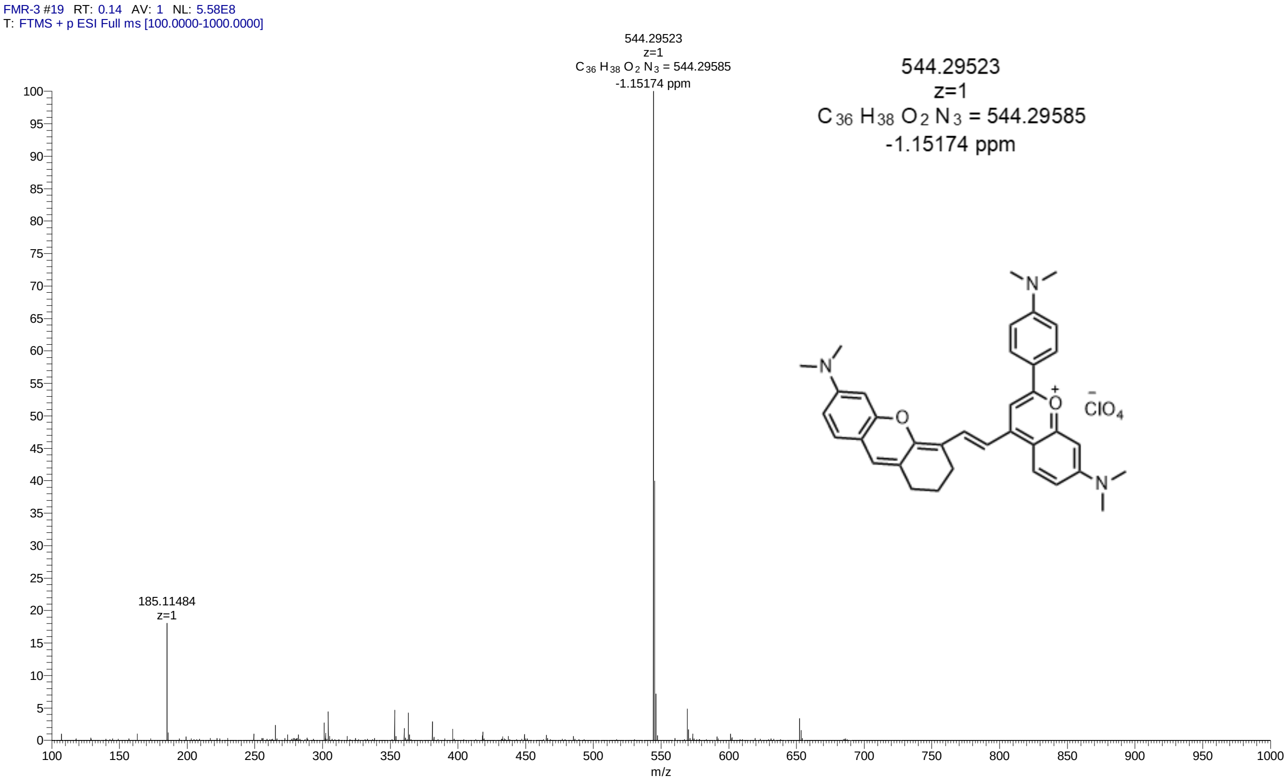

^{1}H NMR spectrum of **FMR-1015**.

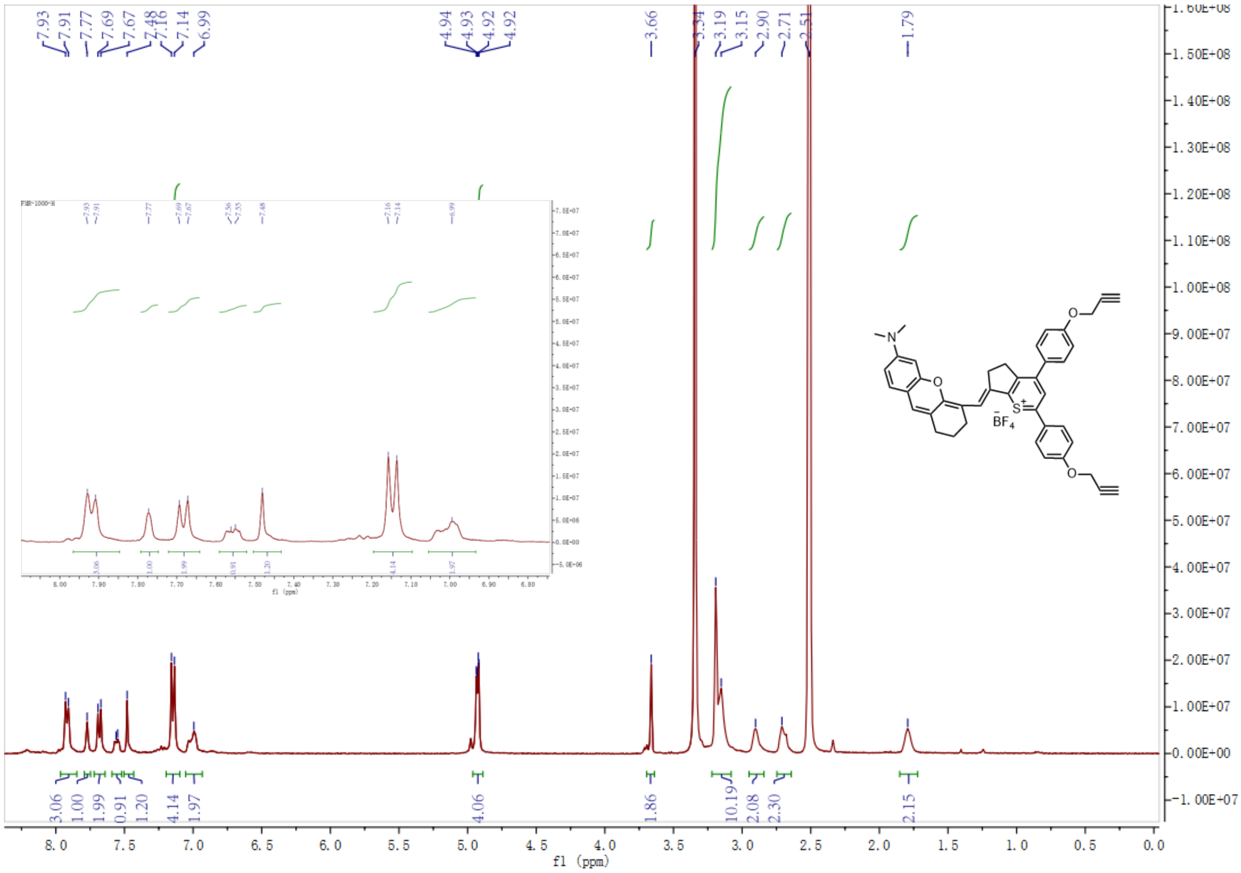

^{13}C NMR spectrum of **FMR-1015**.

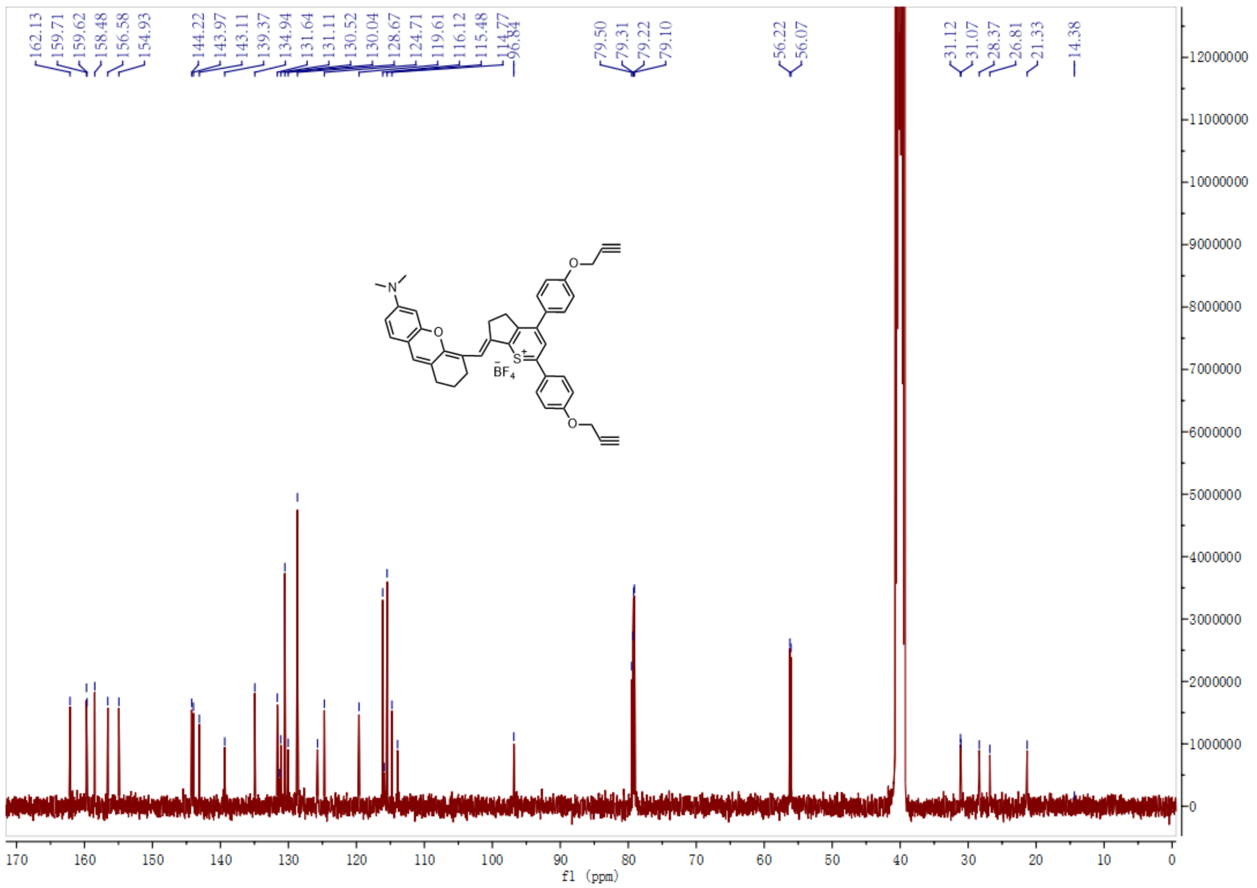

HRMS spectrum of **FMR-1015**.

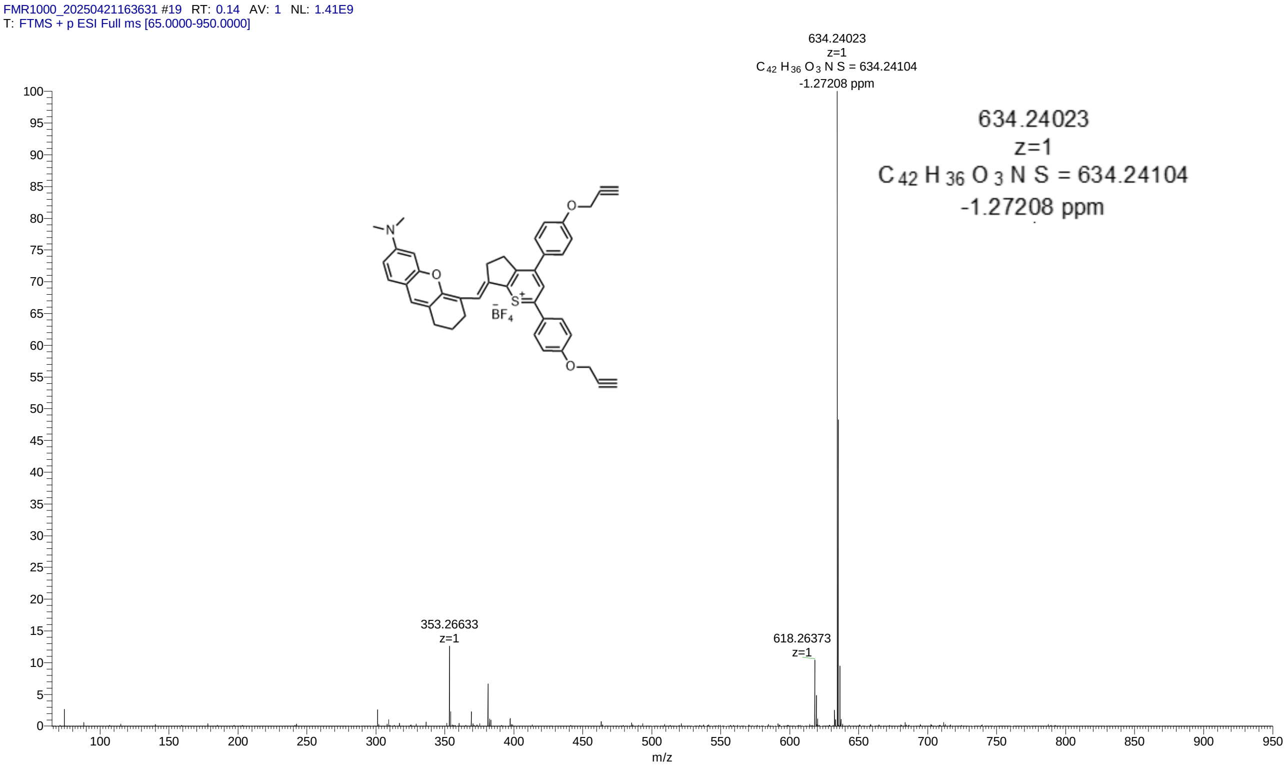

^{1}H NMR spectrum of **FMR-1105**.

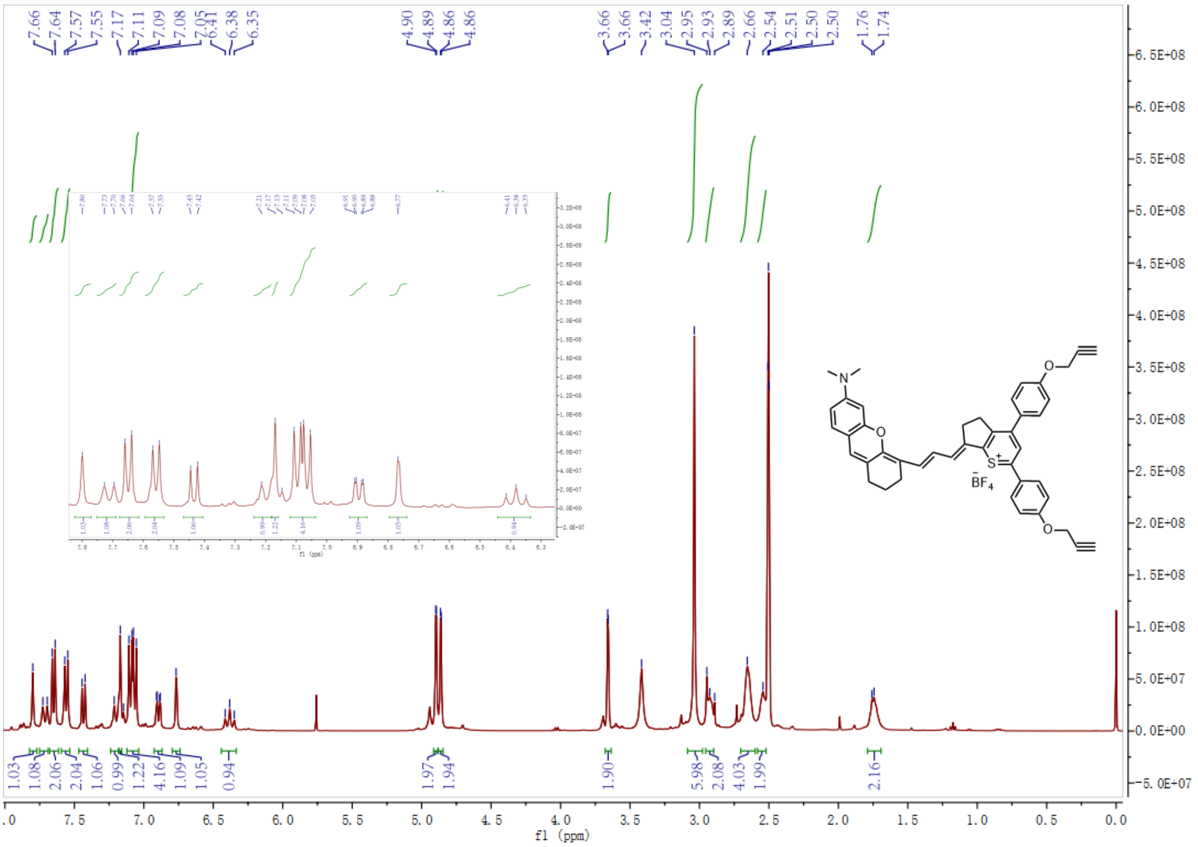

^{13}C NMR spectrum of **FMR-1105**.

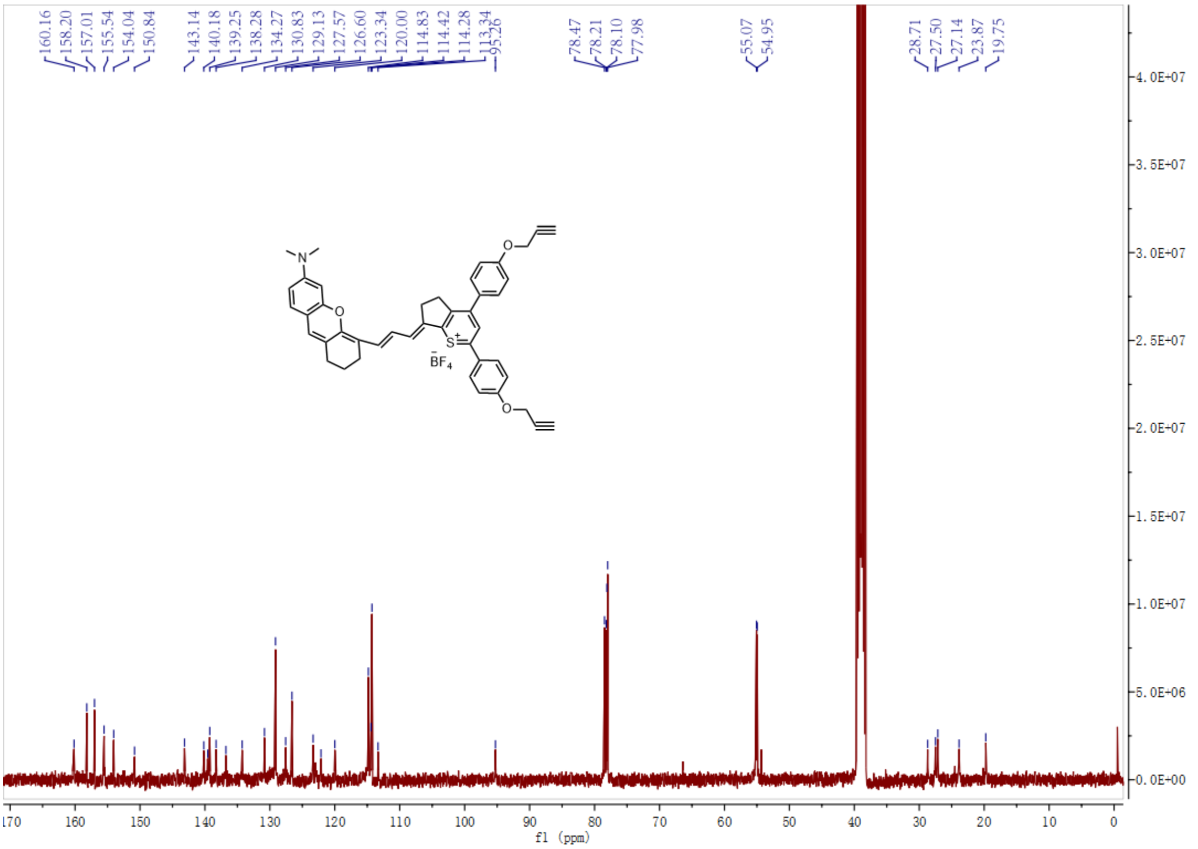

HRMS spectrum of **FMR-1105**.

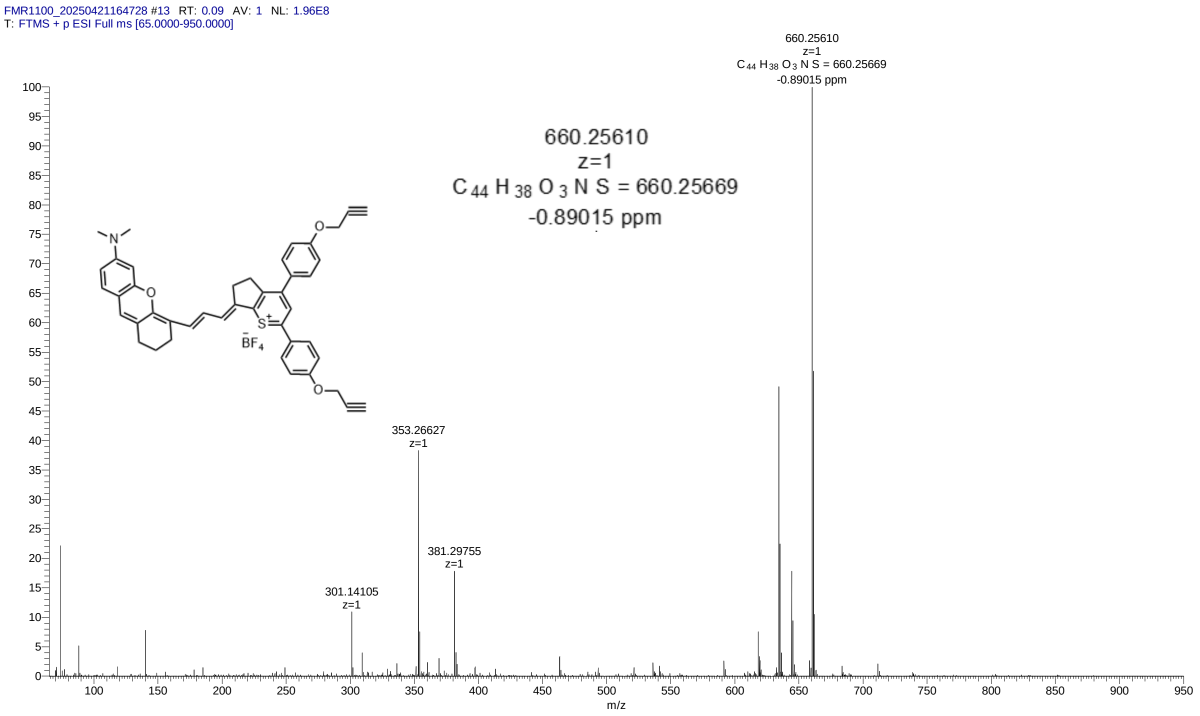

MALDI-TOF-MS spectrum of **FMR-1105-PEG**.

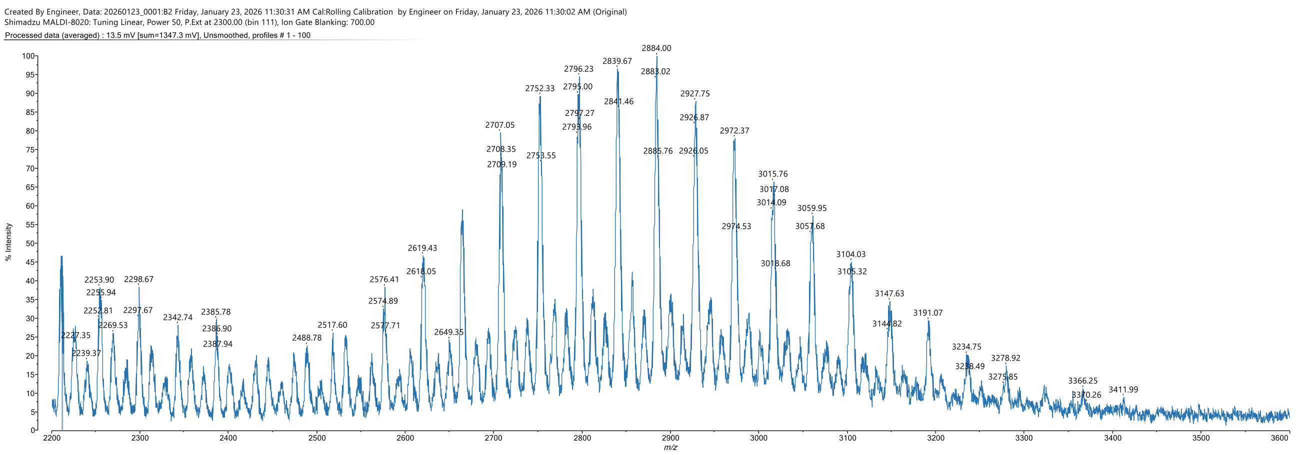

References

(1) Guo, J. M.; Zhu, Y. L.; Qu, Y. Q.; Zhang, L. F.; Fang, M. X.; Xu, Z. H.; Wang, T. B.; Qin, Y. F.; Xu, Y. H.; Li, Y. Y.; et al. Structure Tailoring of Hemicyanine Dyes for Shortwave Infrared Imaging. *J. Med. Chem.* **2024**. DOI: 10.1021/acs.jmedchem.4c01662.
